# Integrative analysis of the role of *BOLA2B* in human pan-cancer

**DOI:** 10.3389/fgene.2023.1077126

**Published:** 2023-02-27

**Authors:** Mingxing Liang, Yinjiao Fei, Yalin Wang, Wenquan Chen, Zhen Liu, Di Xu, Hongyu Shen, Honglei Zhou, Jinhai Tang

**Affiliations:** ^1^ Department of General Surgery, The First Affiliated Hospital of Nanjing Medical University, Nanjing, China; ^2^ School of Clinical Medicine, Xuzhou Medical University, Xuzhou, China

**Keywords:** BOLA2B, pan-cancer, immune infiltration, mutation, proliferation

## Abstract

**Objective:** *BOLA2B* is a recently discovered protein-coding gene. Here, pan-cancer analysis was conducted to determine the expression patterns of *BOLA2B* and its impact on immune response, gene mutation, and possible molecular biological mechanisms in different tumors, together with investigating its potential usefulness for cancer prognosis.

**Methods:** Data on *BOLA2B* expression and mutations were downloaded from TCGA and GTEx databases. Clinical survival data from TCGA were used to analyze the prognostic value of *BOLA2B*. TIMER and ESTIMATE algorithms were used to assess correlations between *BOLA2B* and tumor-infiltrating immune cells, immune cytokines, and immune scores.

**Results:** BOLA2B was found to be highly expressed at both mRNA and protein levels in multiple tumors, where it was associated with worse overall survival (OS), disease-specific survival (DSS), and progression-free interval (PFI) in all cancers apart from ovarian cancer. *BOLA2B* was also found to be positively correlated with copy number variation (CNV), and mutations in *TP53, TTN*, and *MUC16* were found to influence *BOLA2B* expression. Post-transcriptional modifications, including m5C, m1A, and m6A, were observed to regulate *BOLA2B* expression in all cancers. Functional analysis showed that *BOLA2B* was enriched in pathways associated with iron–sulfur cluster formation, mTOR-mediated autophagy, and cell cycle inhibition. Decreased *BOLA2B* expression induced the proliferation of breast cancer cells and G2/M cell cycle arrest.

**Conclusion:** *BOLA2B* was found to be highly expressed in malignant tumors and could be used as a biomarker of poor prognosis in multiple cancers. Further investigation into *BOLA2B*’s role and molecular functions in cancer would provide new insights for cancer diagnosis and treatment.

## 1 Introduction

Cancer has long been a major disease affecting human health. The Human Genome Project, begun in 1990 and completed in 2003, has helped researchers explore the internal causes and development of tumors from the genetic level, thus greatly advancing both cancer diagnosis and treatment. Recently, the Telomere-to-Telomere (T2T) consortium completed a new human genome project, the T2T-CHM13. This provided a more comprehensive genome, identifying and mapping approximately 200 million base pairs, including 99 protein-coding genes and nearly 1857 non-coding genes.

*BOLA2B*, one of the newly mapped 99 protein-coding genes, belongs to the BolA protein family that is strongly conserved in both prokaryotes and eukaryotes. There are four BolA proteins, namely, BolA1, BolA2, BolA3, and BolA4 (Couturier et al., 2014). BolA1 is expressed in most organisms apart from archaea and cyanobacteria (Guinote et al., 2014), while BolA2 and BolA3 are only present in eukaryotes. BolA4 is mainly expressed in prokaryotes and photosynthetic eukaryotes (Couturier et al., 2014). Structurally, the four BolA proteins contain four α-helices and three β-sheets, with a helix-turn-helix motif that is involved in nucleic acid binding (Roret et al., 2014). In cells, BolA2 is expressed in both the nuclear and cytoplasmic compartments, while the other three BolA family members are found in the chloroplast and mitochondria (Talib and Outten, 2021).

In terms of BolA protein functions and regulatory mechanisms, previous studies have reported that the BolA family is associated with cell morphology and cell division. BolA family members appear to be important in the maintenance of normal cell shape. The upregulated expression of BolA proteins in *E. coli* was found to induce the expression of proteins involved in cell wall synthesis, such as PBP5, PBP6, and AmpC (Santos et al., 2002). BolA proteins are also associated with the regulation of cell wall biosynthetic enzymes and interact with the glutaredoxin (GRX) reductase (Huynen et al., 2005). Studies have also shown the association of BolA with iron regulation, where it functions as an essential protein cofactor to control the balance of redox reactions within cells (Rey et al., 2019). Mammals contain four major classes of iron-containing proteins, namely, iron-containing hemeproteins, iron–sulfur (Fe–S) enzymes, iron storage and transport proteins, and other iron-containing or activating enzymes. Of these, Fe–S clusters are the oldest protein cofactors in Fe–S enzymes, responsible for the maintenance of electron transport and homeostasis (Frey et al., 2016). In normal physiological conditions, GRX3 forms complexes with 2Fe–S homodimers, while under oxidative stress, BolA2 interacts with GRX3 to form a [2Fe–S] heterocomplex.

BolA2B has been reported to be involved in the initiation and development of ovarian and liver cancer through its interaction with several metal ion-binding genes, such as *GLRX3*, *GLRX5*, and *WRNIP1*, to activate oxidative phosphorylation, glutathione metabolism, and the proteasomal pathway, leading to cancer progression (Luo et al., 2019; Zhu and Xiao, 2021). Iron dysregulation is known to be related to cancer development. In the Hep3b cell line, knockdown of *BOLA2B* reduced the level of iron staining, indicating that BolA2 plays a key role in iron homeostasis in cancer cells (Luo et al., 2019). In addition to mediating iron homeostasis, BOLA2 was shown to interact with p62 to activate mTORC1 in transplanted hepatocellular carcinoma (HCC) tumor cells and to stimulate c-MYC oncogenic activity in liver cancer, suggesting that BolA2B may act as a transcription factor regulating additional genetic targets (Hunecke et al., 2012; Luo et al., 2019).

Here, we attempted to elucidate a comprehensive view of the role of *BOLA2B* in pan-cancer in terms of expression patterns, RNA modification, mutant profiles, and its relationships with immune-related factors and cancer prognosis. *BOLA2B* is a gene that has only recently been discovered in the T2T-CHM13 human genome project, and thus, little is known about it. We hypothesized that it can exert direct functions in cells. Our findings suggested that *BOLA2B* plays important roles in multiple tumors and represents a promising therapeutic target for cancer treatment.

## 2 Materials and methods

### 2.1 Primary data collection and processing

The mRNA sequencing data from 19, 131 samples were downloaded from TCGA, TARGET, and GTEx projects hosted at the University of California at Santa Cruz (UCSC) Cancer Genome Browser (https://xenabrowser.net/datapages/?cohort=TCGA%20TARGET%20GTEx). Additional data were obtained from the literature (Malta et al., 2018). The data were cleaned, leading to the retention of 38 cancer types. Data on clinical information, copy number variation (CNV), tumor mutation burden (TMB), and single-nucleotide polymorphisms (SNPs) were downloaded from the UCSC database at the same time.

The transcriptome data were converted into log2 (TPM+1) to assess differences between normal and tumor tissues. For protein expression, we downloaded mass spectrometry data from the Clinical Proteomic Tumor Analysis Consortium (CPTAC) database for further analysis (Chandrashekar et al., 2017; Chandrashekar et al., 2022). Z-values represented standard deviations from the median across the given cancer types.

The codes used in this study are available in the GitHub repository (https://github.com/Honglei-Zhou/BOLA2B.git). The genes involved in our study were listed as Appendix A1 at the end of article.

### 2.2 Survival prognosis and clinical manifestation analysis

Cox proportional hazard ratio regression analysis was performed with the ‘survival’ package in R to evaluate the dependency of the survival data such as overall survival (OS), disease-specific survival (DSS), and progression-free interval (PFI) on gene expression. The data were divided into groups according to the median level of *BOLA2B* expression; this was applied to all cancer types. Log-rank tests were used to determine the statistical significance of the hazard ratios. Forest plots and Kaplan–Meier curves were drawn with the R packages “ggplot” and “survminer,” respectively.

### 2.3 Analysis of RNA modifications

Data on mRNA modifications were collected and analyzed. Writer, eraser, and reader genes that were highly associated with the m1A, m5C, and m6A modifications were identified. Pearson’s correlation coefficients were used to determine the associations between the identified genes and *BOLA2B* expression in various cancers.

### 2.4 Immune infiltration enrichment analysis

The *BOLA2B* gene expression profiles were extracted separately for each tumor type and were then mapped to the gene symbol, and the infiltration scores of B cells, CD4 T cells, CD8 T cells, neutrophils, macrophages, and dendritic cells (DCs) were determined using the “IOBR” package in R.

The expression profiles of immune-related genes were divided into inhibitory and stimulatory categories and were analyzed using the same process. The corr. test function in the ‘psych’ R package was used to calculate Pearson’s correlation coefficients. The ESTIMATE score for each tumor was calculated using the R package “ESTIMATE.”

The TMB for each tumor was calculated using the “TMB” function in “MFT” tools. The TMB and gene expression data of the samples were integrated into a single matrix, and a log2 (x+0.001) transformation was applied to the expression data.

### 2.5 Pan-cancer gene mutation profiles

The *BOLA2B* CNV data were downloaded from the Genomic Data Commons (GDC) Data Portal. The data were processed using GISTIC software. Sample barcodes were used to combine the clinical survival data with the CNV data. The ‘survival’ package in R was used to fit data on survival time and survival status with the CNV data, and log-rank tests were used to examine differences in survival rates.

Exon sequencing data for nearly 30 tumors are available in TCGA database. Missense_Mutation, Frame_Shift_Del, Non-sense_Mutation, In_Frame_Ins, Splice_Site, In_Frame_Del, and Frame_Shift_Ins were considered as deleterious mutations in this analysis.

Pearson’s correlation coefficients were used to assess interactions between microsatellite instability (MSI) and *BOLA2B* gene expression. RNA-based stemness scores (RNAss) indicated that the RNA expression-based score drives the main stemness (Malta et al., 2018).

### 2.6 Gene enrichment and biological pathway analysis

The Search Tool for the Retrieval of Interacting Gene (STRING) database (https://string-db.org/) is a web application that analyzes potential protein–protein interaction networks using multiple algorithms. We used the single protein name ‘BolA2B’ and the organism ‘*Homo sapiens*’ to obtain a map of predicted interacting proteins. To obtain more reliable results, we set specific parameters with a minimum required interaction score set as ‘medium confidence (0.4)’ and the maximum number of first-shell interactors to ‘not more than 50 interactors’. Functional enrichment of the genes was analyzed using Gene Ontology (GO), including information on biological process, molecular function, and cellular components, and Kyoto Encyclopedia of Genes and Genomes (KEGG).

### 2.7 Plasmid transfection and antibodies

BOLA2B shRNA1 and shRNA2 were amplified by PCR and subcloned into the pLKO.1 vector. The sequence of BOLA2B shRNA1 was GGC​ACG​TGA​GCG​ACA​GAA​ATG​TTC​AAG​AGA​CAT​TTC​TGT​CGC​TCA​CGT​GCC, and the sequence of BOLA2B shRNA2 was GCG​AGA​AGC​TGC​AGC​GGG​ACC​TTC​AAG​AGA​GGT​CCC​GCT​GCA​GCT​TCT​CGC. The Ctrl group used the same plasmid vector as the experimental group, except for the shRNA sequence. Lentivirus vectors were used to transfect MDA-MB-231 and SKBR3 cells for 24 h, followed by selection with 2 μg/ml puromycin to obtain cells with stable expression.

### 2.8 Cell culture and treatment

The human breast cancer cell lines MDA-MB-231 and SKBR3 were cultured in Dulbecco’s modified Eagle medium (DMEM) supplemented with 10% fetal bovine serum (FBS) and 1% penicillin/streptomycin at 37°C with 5% CO_2_. For the measurement of cell viability, cells of different groups were seeded in 96-well plates at the same density of 5 × 10^3^/mL, and the Cell Counting Kit-8 (Beyotime, #C0038) solution was added at the specific time points. The cells were then incubated for 2 h at 37°C, and the absorbance was read at 450 nm. Cell proliferation was measured using the EdU Cell Proliferation Assay Kit (RiboBio, #C10310-1), according to the manufacturer’s instructions. Nuclei were counterstained with 1 μg/ml of DAPI (Beyotime, #C1002). The proportion of cells stained with EdU was examined by fluorescence microscopy (Carl Zeiss AG, Axio Vert. A1).

Cell cycle changes were monitored by flow cytometry. Cells were harvested and fixed with 70% cold ethanol at -20°C overnight. The DNA was stained with propidium iodide (PI), according to the manufacturer’s instruction (Multi Sciences. #CCS01).

Specimens of different cancers were collected from the First Affiliated Hospital of Nanjing Medical University. The project was approved by the Ethics Committee of Jiangsu Province People’s Hospital, with the code number 2020-SR-477. Immunohistochemistry (IHC) was performed using kits from MXB Biotechnologies, #KIT-9710 and #DAB-2031, in accordance with the manufacturer’s instructions. IHC scores for BolA2B staining were determined by light microscopy and calculated as the staining intensity score multiplied by the staining percentage score. The staining intensities were classified as follows: no staining, 0; weak staining, 1; moderate staining, 2; strong staining, 3; and very strong staining, 4. The staining percentages were classified as 0%–10%, 0; 10%–25%, 1; 25%–50%, 2; 50%–75%, 3; and 75%–100%, 4.

### 2.9 Western Blot

Targeted cell proteins were extracted with the RIPA buffer. Proteins of different molecular weights were separated by SDS-PAGE. After electransferring onto the PVDF membrane, 5% skim milk was used to block extra places for 2 h. Then, the membrane was incubated with targeted primary antibodies at 4°C overnight. The antibodies used in the study were against BolA2B (Proteintech, # 26080-1-AP), tubulin (Proteintech, #11224-1-AP), CDK6 (Cell Signaling Technology [CST], #13331), cdc6 (CST, #3387), cyclin B1 (CST, #12231), cyclin D1 (CST, #55506), cyclin E1 (CST, #20808), p21 (CST, #2947), p-AKT (CST, #4060), AKT (CST, #9272), p-mTOR (CST, #5536), mTOR (CST, #2983), p-ERK (CST, #4370), and ERK (CST, #4695). The next day, the membrane was incubated with secondary antibodies (CST, #7074P2). The bands could be observed by Immobilon™ Western Chemiluminescent HRP Substrate (Millipore, United States)

### 2.10 Statistical analysis

Continuous data were analyzed by t-tests, with paired t-tests used for comparing differences between groups. Categorical data were analyzed using χ2 tests. Pearson’s correlation analysis was used for comparing associations between groups of continuous data. Two-sided *p*-values less than 0.05 were considered statistically significant, and asterisks were used in some figures to indicate the level of significance: *= (*p* < 0.05), **= (*p* < 0.01), and *** = (*p* < 0.001).

## 3 Results

### 3.1 *BOLA2B* expression in different cancers

**FIGURE 1:**
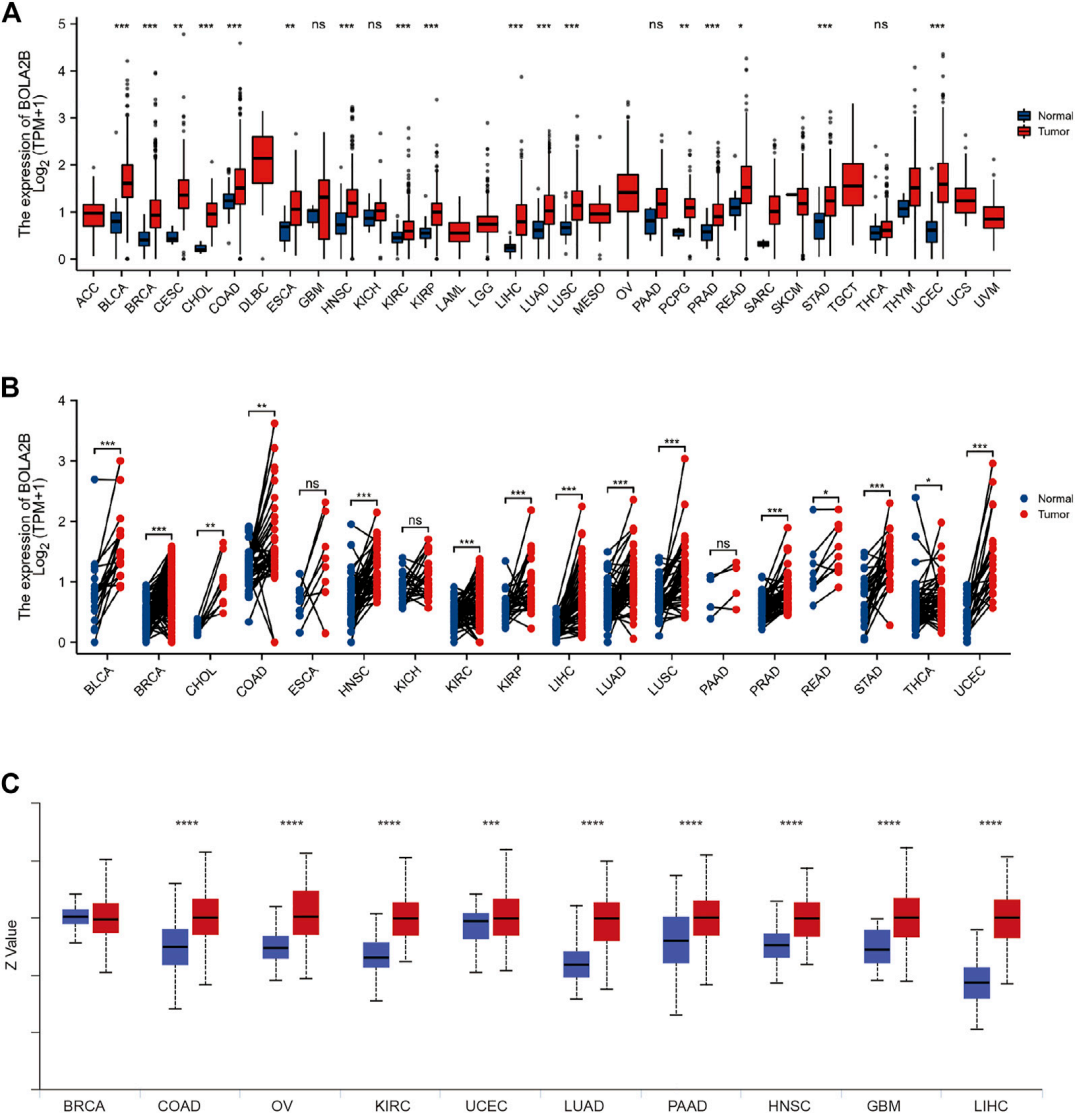
High expression of BOLA2B in cancers. **(A)** The mRNA expression of BOLA2B across 33 cancer types from TCGA data. **(B)** The mRNA expression difference from paired cancer and para-cancer in 18 cancer types. **(C)** The protein expression of BOLA2B from the CPTAC dataset. Red color represents the tumor sample, and blue color refers to normal samples. *p < 0.05, **p < 0.01, and ***p < 0.001.

We analyzed *BOLA2B* mRNA expression between tumor and normal tissues in 33 cancer types. As shown in Figure 1A, *BOLA2B* was upregulated in BLCA, BRCA, CESC, CHOL, COAD, ESCA, HNSC, KIRC, KIRP, LIHC, LUAD, LUSC, PCPG, PRAD, READ, STAD, and UCEC tumor samples compared to normal tissues. Paired comparisons also verified that *BOLA2B* was a cancer-related factor (Figure 1B). BLCA, BRCA, CHOL, COAD, HNSC, KIRC, KIRP, LIHC, LUAD, LUSC, PRAD, READ, STAD, THCA, and UCEC tumor samples all showed higher expression compared to their paired adjacent normal tissues. We then analyzed the BolA2B protein levels in different cancers. Analysis of data from the CPTAC database showed that COAD, OV, KIRC, UCEC, LUAD, PAAD, HNSC, GBM, and LIHC tumor tissues all expressed increased levels of BolA2B; the exception was BRCA, as shown in Figure 1C. These results indicated the association of increased *BOLA2B* expression with multiple tumor types.

### 3.2 Low expression of BOLA2B predicted better prognosis in multiple cancers

**FIGURE 2:**
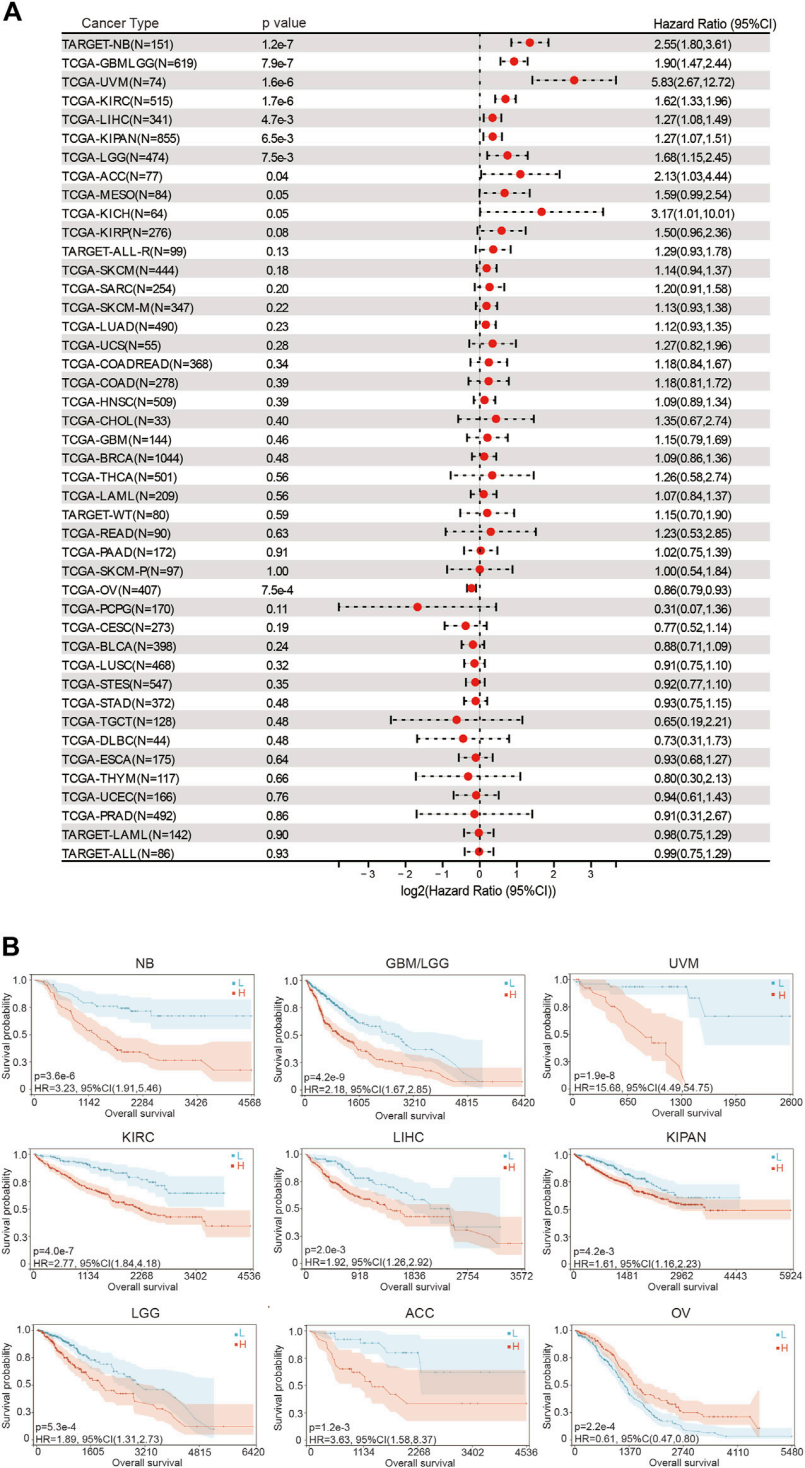
Association of BOLA2B with poor overall survival prognosis in cancers. **(A)** Hazard ratio of BOLA2B expression in different cancers from the TCGA dataset. **(B)**Representative images of BOLA2B influence on prognosis of NB, GBMLGG, UVM, KIRC, LIHC, KIPAN, LGG, ACC, and OV. The x-axis represents the number of days.

We then evaluated the prognostic significance of *BOLA2B* in pan-cancer analysis. In the overall survival (OS) assessment, the forest plot showed that the expression was significantly correlated with NB (HR = 2.55, 95%CI: 1.80–3.61), GBMLGG (HR = 1.90, 95%CI: 1.47–2.44), UVM (HR = 5.83, 95%CI: 2.67–12.72), KIRC (HR = 1.62, 95%CI: 1.33–1.96), LIHC (HR = 1.27, 95%CI: 1.08–1.49), KIPAN (HR = 1.27, 95%CI: 1.07–1.51), LGG (HR = 1.68, 95%CI: 1.15–2.45), ACC (HR = 2.13, 95%CI: 1.03–4.44), and OV (HR = 0.86, 95%CI: 0.79–0.93) (Figure 2A). Kaplan–Meier curves were used to evaluate differences in OS between the high- and low-*BOLA2B* subgroups in different types of cancer. As shown in Figure 2B, high expression of *BOLA2B* predicted better OS in OV patients, while in other cancers, such as NB, GBM, UVM, KIRC, LIHC, KIPAN, LGG, and ACC, patients with low BOLA2B expression showed better OS rates.

Similar results were obtained in the disease-specific survival (DSS) analysis. The hazard ratios of *BOLA2B* were significant for GBMLGG (HR = 1.87, 95%CI: 1.43–2.44), LGG (HR = 1.63, 95%CI: 1.10–2.42), KIRP (HR = 2.36, 95%CI: 1.44–3.89), KIRAN (HR = 1.69, 95%CI: 1.38–2.08), KIRC (HR = 2.06, 95%CI: 1.64–2.58), THYM (HR = 3.88, 95%CI: 1.09–13.75), LIHC (HR = 1.26, 95%CI: 1.03–1.55), UVM (HR = 5.39, 95%CI: 2.42–11.99), and OV (HR = 0.85, 95%CI: 0.78–0.93), as shown in Supplementary Figure 1A. Kaplan–Meier plots showed the specific survival probability for every significant cancer influenced by *BOLA2B* expression (Supplementary Figure 1B).

Finally, analysis of the progression-free interval (PFI) indicated that high levels of *BOLA2B* mRNAs were also strongly associated with worse prognosis in GBMLGG (HR = 1.81, 95%CI: 1.42–2.32), LGG (HR = 1.41, 95%CI: 1.05–1.90), KIRP (HR = 2.06, 95%CI: 1.21–3.53), KIPAN (HR = 2.17, 95%CI: 1.49–3.16), KIRC (HR = 3.36, 95%CI: 2.12–5.32), LIHC (HR = 1.67, 95%CI: 1.22–2.30), SKCM-M (HR = 1.32, 95%CI: 1.02–1.71), UVM (HR = 4.55, 95%CI: 1.99–10.42), KICH (HR = 3.99, 95%CI: 1.17–13.64), UCS (HR = 3.04, 95%CI: 1.45–6.36), and BLCA (HR = 0.56, 95%CI: 0.38–0.82) (Supplementary Figure 2A). Kaplan–Meier curves confirmed these results (Supplementary Figure 2B). These findings demonstrated the risks associated with abnormally increased *BOLA2B* expression and suggest that *BOLA2B* could be used as a potential biomarker to predict patient survival in multiple cancers.

### 3.3 mRNA modification landscapes of *BOLA2B*

**FIGURE 3:**
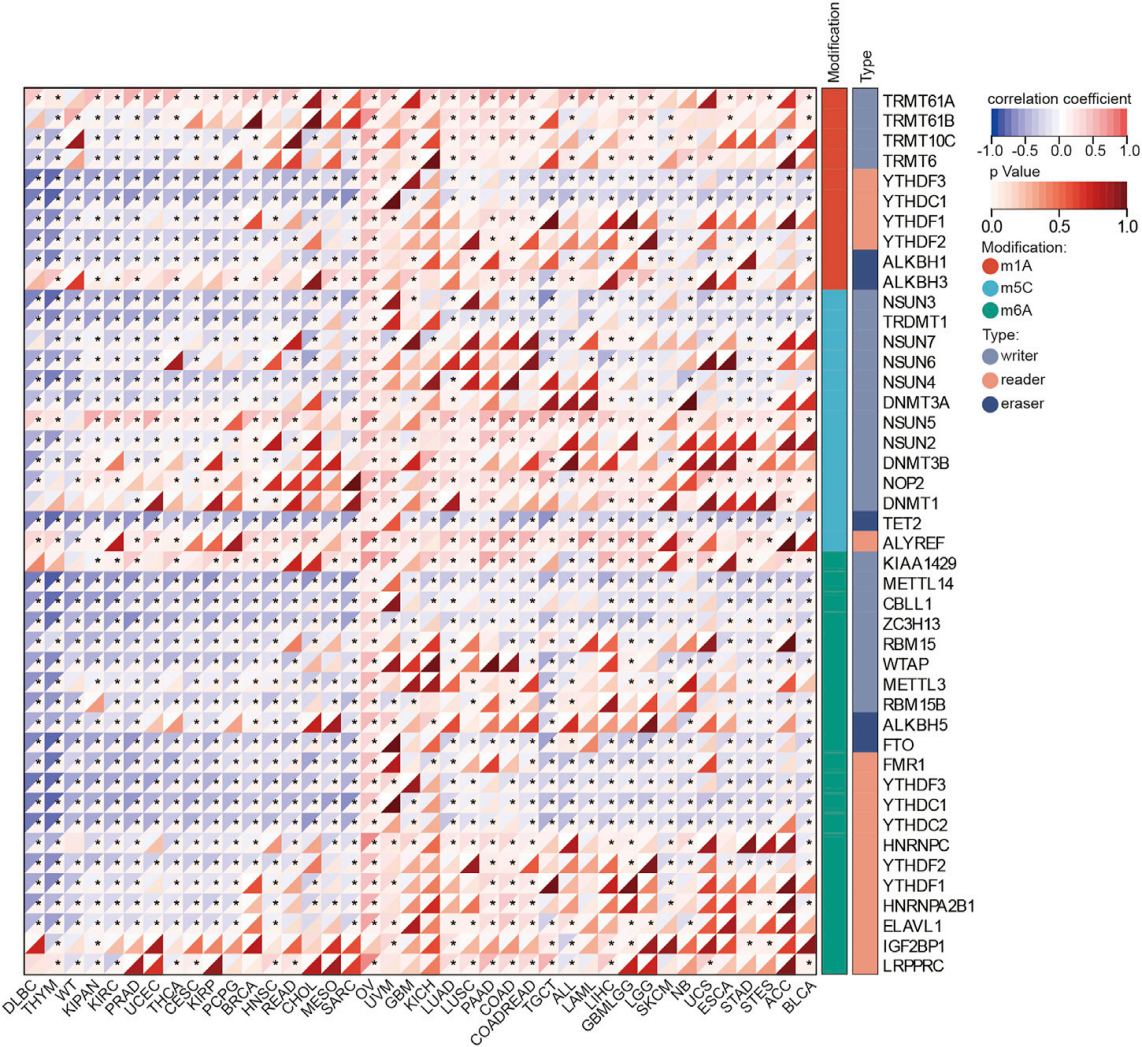
Heatmaps of BOLA2B correlation with mRNA modification. The color depth in the heatmap represented correlation coefficients of BOLA2B and mRNA modification markers. *p < 0.05, **p < 0.01, and ***p < 0.001.

mRNA modifications such as m1A, m5C, and m6A, are known to regulate protein translation. We analyzed the correlations between *BOLA2B* mRNA expression and the expression of related genes. As shown in Figure 3, correlations between genes involved in m1A, m5C, and m6A modifications and *BOLA2B* mRNA expression were present in numerous cancers.

BOLA2B m1A modifications (Figure 3) in DLBC, THYM, WT, KIPAN, PRAD, UCEC, THCA, CESC, KIRP, PCPG, BRCA, HNSC, READ, CHOL, MESO, and SARC were found to be negatively correlated with a reader gene, while positive correlations were seen with writer genes in cancers such as LUAD, LUSC, PAAD, COAD, COADREAD, TGCT, ALL, LAML, LIHC, GBMLGG, LGG, SKCM, NB, UCS, ESCA, STAD, STES, ACC, and BLCA. In terms of m5C modifications, the results showed that all the investigated cancers except OV were negatively associated with the eraser gene TET2. All cancers were highly positively associated with the writer gene NSUN5 and reader gene ALYREF synchronization. Regarding m6A, the writer gene KIAA1429 was positively correlated with *BOLA2B* across all cancers, while other m6A writer genes showed negative correlations with *BOLA2B* in most cancers. Of these cancers, OV was unique as it was positively associated with m1A, m6A, and m5C, regardless of their modifier regulators. These findings suggest that there are three different methods of post-transcriptional regulation of *BOLA2B* in different tumors.

### 3.4 Correlation between *BOLA2B* expression and the immune response

**FIGURE 4:**
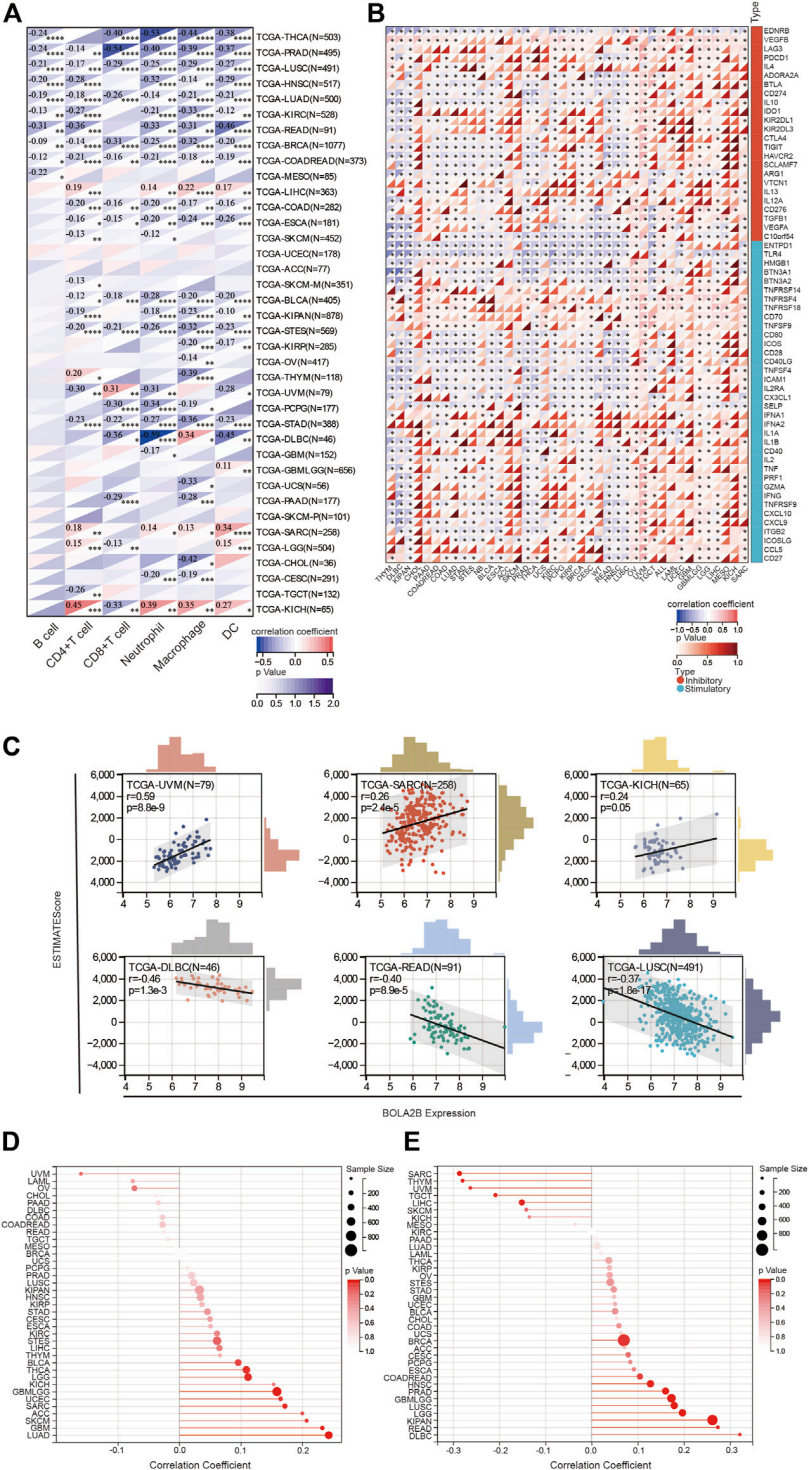
Correlation of BOLA2B expression with immune response. **(A)** Correlation of BOLA2B with key immune cells in TCGA. **(B)** Heatmaps of the relationship of stimulatory and inhibitory immune cytokines with BOLA2B across cancers. **(C)** Representative ESTIMATE evaluation of BOLA2B expression in the top three cancers. **(D)** The lollipop plot of BOLA2B association with tumor purity in cancers. **(E)** The lollipop plot of BOLA2B association with tumor mutational burden (TMB) in cancers.

Next, we analyzed interactions between *BOLA2B* expression and immune cell infiltration. As shown in Figure 4A, *BOLA2B* expression was negatively correlated with immune cell infiltration in most tumors. However, in LIHC, *BOLA2B* expression was positively associated with CD4^+^ T cells, neutrophils, macrophages, and DCs, all of which are involved in humoral immunity. Similar results were observed in KICH and SARC tumors. In UVM, *BOLA2B* expression showed a positive correlation with CD8^+^ T cells but was negatively correlated with other immune cells such as CD4^+^ T cells, neutrophils, macrophages, and DCs, suggesting that *BOLA2B* may affect cellular immunity in UVM.

Analysis of the association between *BOLA2B* and immune-related proteins and cytokines indicated that *BOLA2B* was negatively correlated with several immune-associated proteins such as EDNRB, ENTPD1, and TLR4 in most tumors, as shown in Figure 4B. We also found that OV, UVM, and SARC had specific differences in terms of immunity; also, in OV, *BOLA2B* was highly positively correlated with the regulation of immune cytokines.

In addition, we evaluated the effects of *BOLA2B* on the immune response in 33 cancers using the ESTIMATE algorithm. The ESTIMATE score consists of the purity score within the tumor and the immune score of the tumor. As shown in Figure 4C, the ESTIMATE score of UVM tumor was strongly associated with *BOLA2B* expression, in contrast to other tumors, such as DLBC, READ, and LUSC, where negative correlations were observed. Further analysis revealed that of all cancers, UVM showed the highest correlation with *BOLA2B* expression, in both the immune and stromal scores (Supplementary Figures 3A, B). DLBC, on the other hand, showed the most negative correlation with *BOLA2B* in the ESTIMATE score, which was largely due to the immune score (Supplementary Figures 3A, B). For both READ and LUSC tumors, *BOLA2B* expression influenced both immune and stromal aspects (Supplementary Figures 3A, B).

It is well known that tumor tissues consist of both tumor and non-tumor cells, including immune cells, stromal cells, and mesenchymal cells. Tumor purity is significantly associated with the clinical characteristics, genomic expression, and biological properties of cancers. We analyzed the effects of *BOLA2B* expression on tumor purity. As shown in the lollipop plot in Figure 4D, the tumor purity of LUAD, GBM, and SKCM cancers was strongly associated with *BOLA2B*. In addition, the interactions between the TMB and *BOLA2B* expression were investigated. As seen in Figure 4E, the lollipop plot shows that *BOLA2B* expression in DLBC, READ, and KIPAN was positively correlated with TMB but was negatively correlated in SARC, THYM, and UVM.

### 3.5 BOLA2B mutation profiles and their impact on cancers

**FIGURE 5:**
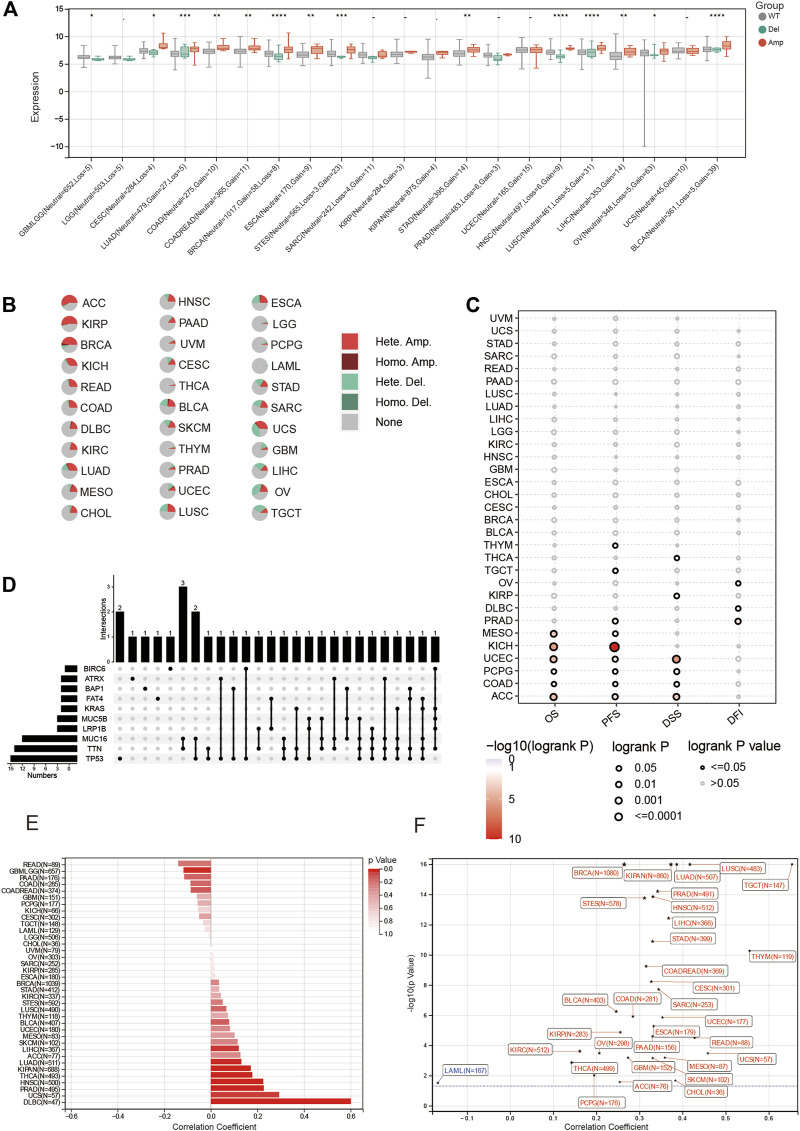
Mutation portray of BOLA2B across cancers. **(A)** Differences of BOLA2B mutation type including WT, deletion, and amplification in different cancers. **(B)** Pie chart of BOLA2B amplification in cancers. **(C)** Copy number variation (CNV) of BOLA2B indication on the Prognosis Index. **(D)** Upset plot of possible genes mutation affected BOLA2B expression. **(E)** Bar chart of microsatellite instability (MSI) relationship with BOLA2B. **(F)** Tumor stemness of various cancers influenced by BOLA2B expression.

Mutations in genes often affect tumor occurrence and progression. Here, we explored several types of mutations associated with *BOLA2B* and their influence on various types of tumors. As shown in Figure 5A, the CNVs of *BOLA2B* revealed the presence of copy number amplifications of *BOLA2B* in several tumors, including CESC, LUAD, COAD, COADREAD, BRCA, ESCA, STES, SARC, STAD, HNSC, LUSC, LIHC, OV, and BLCA. These led to the increased expression of *BOLA2B* relative to WT and Del groups. The percentages of each type of *BOLA2B* CNVs in the 33 cancers were then plotted and illustrated in a pie chart. This showed that heterozygous copy number amplification was most prevalent in ACC, KIRP, and BRCA, while there were fewer CNVs in THCA, THYM, LGG, LAML, and PCPG (Figure 5B). We also found that survival prognosis, including OS, PFS, and DSS, was affected by CNVs in ACC, COAD, PCPG, and UCEC (Figure 5C). The relationship between single-nucleotide variants (SNVs) and *BOLA2B* expression was also examined in the different cancers. This showed that mutations in *TP53, TTN*, and *MUC16* played key roles in influencing the expression of *BOLA2B*, and simultaneous mutations in TTN and MUC16 were presented in three different cancers (Figure 5D). This suggests that further exploration of the relationship between *BOLA2B* and mutations in these genes is warranted.

In addition, we explored the relationship between microsatellite instability (MSI) and *BOLA2B*. The occurrence of MSI in tumor tissues is due to a functional breakdown in DNA mismatch repair. The highest correlation between MSI and *BOLA2B* was observed in DLBC (Figure 5E). Finally, we depicted the relationship between tumor stemness and *BOLA2B*. As shown in Figure 5F, the scatter plot indicated that the stemness of TGCT was significantly positively correlated with *BOLA2B*, while that of LAML showed the most negative association with *BOLA2B*. The stemness of most other tumors was positively correlated with *BOLA2B*.

### 3.6 Gene functional enrichment analysis

**FIGURE 6:**
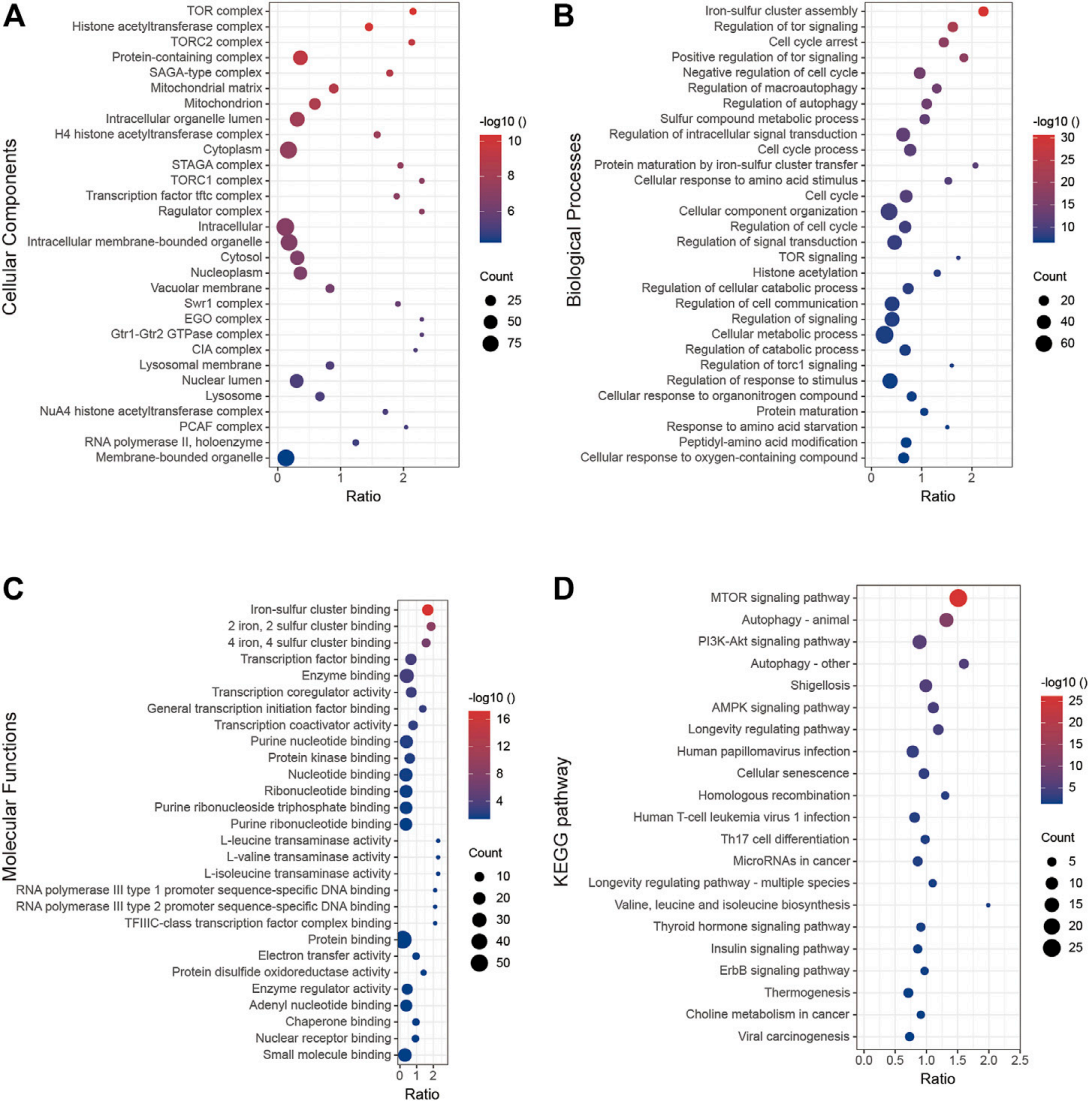
GO and KEGG analysis of BOLA2B. **(A)** BOLA2B possible distribution of cellular components. **(B)** Enrichment of biological processes that BOLA2B might be involved in. **(C)** Predicted molecular function of BOLA2B in cancers. **(D)** KEGG pathway analysis with BOLA2B expression.

In this section, we analyzed the function of *BOLA2B* from a molecular biology perspective. The GO cellular component analysis showed that *BOLA2B* was associated with the TOR complex and histone acetylation (Figure 6A). In the biological process category, *BOLA2B* was associated with the aggregation of iron–sulfur clusters and cell-cycle arrest (Figure 6B), while in the molecular function category, *BOLA2B* was mainly involved in the formation of iron–sulfur clusters of both 2-valent and 4-valent iron and could function as a transcription factor (Figure 6C). KEGG pathway analysis showed that *BOLA2B* was mainly involved in the mTOR, autophagy, and PI3K-AKT pathways (Figure 6D).

### 3.7 The effect of *BOLA2B* on breast cancer cell proliferation

**FIGURE 7:**
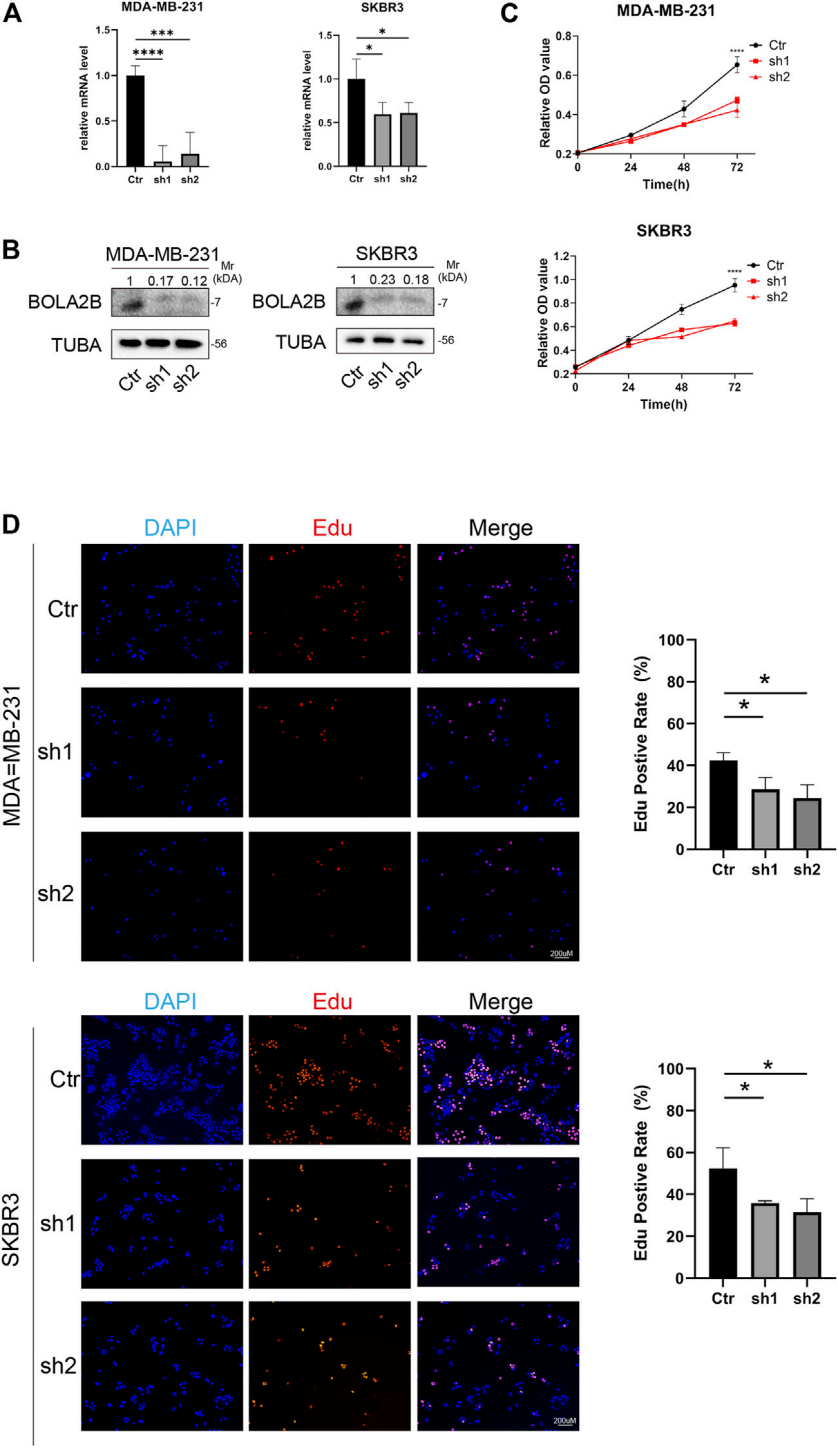
BOLA2B knockdown reduced breast cancer cell proliferation. **(A)** Relative mRNA level of BOLA2B knockdown in MDA-MB-231 and SKBR3. **(B)** Western blot of BOLA2B knockdown in MDA-MB-231 and SKBR3. **(C)** CCK-8 assay about the BOLA2B-shRNA group downregulated MDA-MB-231, and SKBR3 proliferation compared to the control group. **(D)** The EdU proliferation assay was performed, and the ratio of EdU-positive cell underwent statistical analysis. *p < 0.05, **p < 0.01, and ***p < 0.001.

To study the function of *BOLA2B* in cancer, the gene was knocked down in the MDA-MB-231 and SKBR3 breast cancer cell lines using shRNA. As shown in Figure 7A, shRNA1 and shRNA2 both downregulated the BolA2B protein level in MDA-MB-231 and SKBR3 cells. The CCK-8 assay showed that *BOLA2B* knockdown significantly reduced the proliferative capacity of breast cancer cells, as shown in Figure 7B. EdU assays were used to confirm the effects of *BOLA2B* on cell proliferation. As shown in Figure 7C, the percentage of proliferating cells in the *BOLA2B*-knockdown group was lower than that in the control group. These results demonstrated the involvement of *BOLA2B* in the proliferation of breast cancer cells.

### 3.8 Relationship between *BOLA2B* and the cell cycle

**FIGURE 8:**
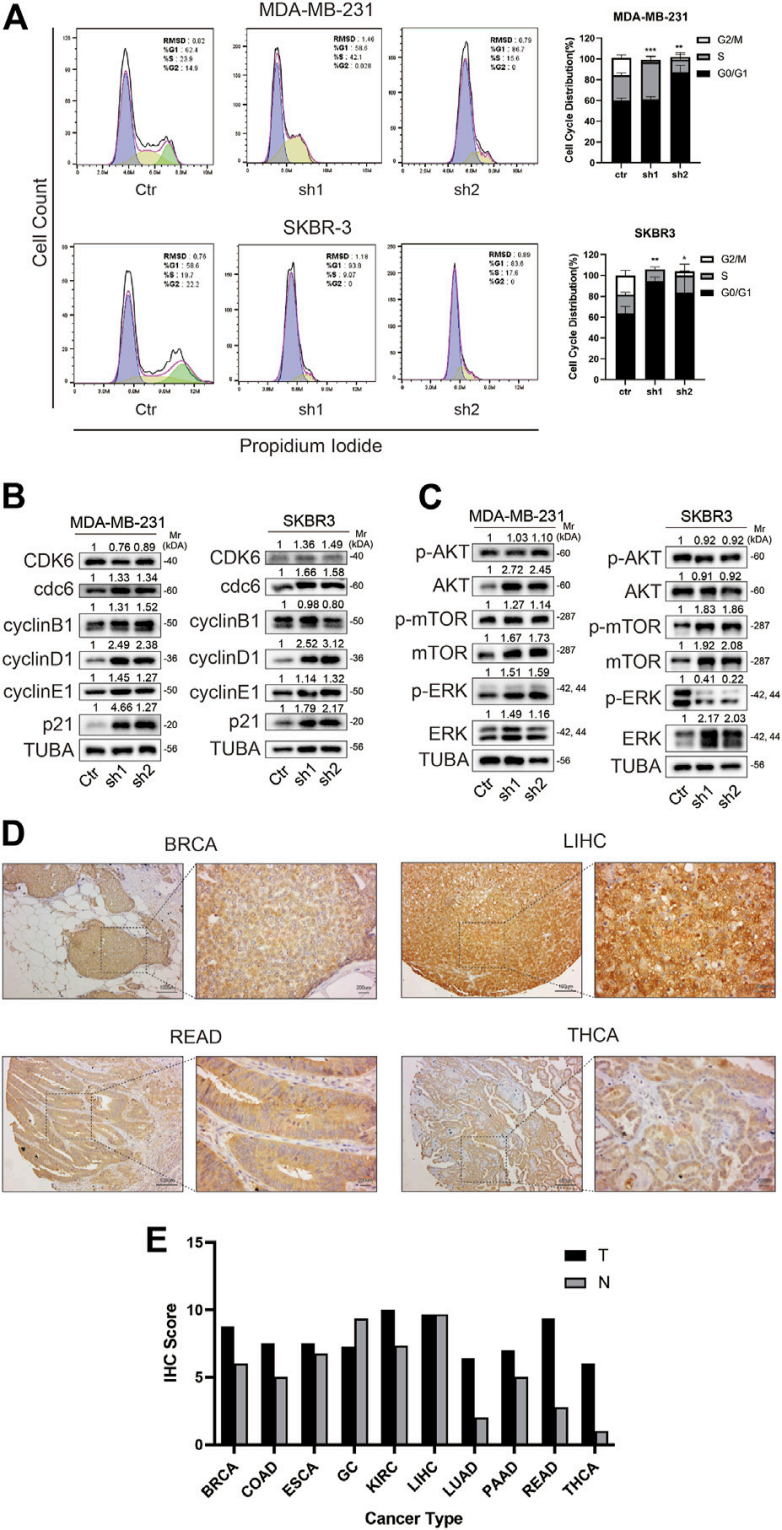
BOLA2B knockdown caused cell cycle arrest and downstream pathway. **(A)** Flow cytometry of cell cycle and statistical histogram of BOLA2B knockdown in MDA-MB-231 and SKBR3. **(B)** Western blot of related cell cycle marker in MDA-MB-231 and SKBR3. **(C)** Western blot of downstream pathway markers in MDA-MB-231 and SKBR3. **(D)** Representative images of IHC staining of BOLA2B in pan-cancer tissues. **(E)** Statistical histogram of IHC scores in different cancer types. *p < 0.05, **p < 0.01, and ***p < 0.001.

Since knockdown of *BOLA2B* reduced cancer cell proliferation, we assumed that it would affect the cell cycle. Flow cytometry showed that BOLA2B knockdown in MDA-MB-231 and SKBR3 cells resulted in G2/M arrest (Figure 8A). Western blotting showed a significant upregulation of cyclin D1 and p21, compared with the control group. The levels of Cdc6 and cyclin E1 were slightly increased, while there was little influence on those of CDK6 and cyclin B1 (Figure 8B).

Previous studies have suggested that *BOLA2B* may be associated with the mTOR and PI3K-AKT pathways. We used western blotting to verify bioinformatics analysis. The results showed that in breast cancer, the signaling activation pattern may be dependent on the cell type. Both AKT and mTOR were upregulated in the *BOLA2B*-knockdown MDA-MB-231 cells, although there was a little change in their phosphorylation status. In SKBR3 cells, on the other hand, the mTOR pathway was significantly activated, while the MAPK pathway was inactivated. No change was seen in the AKT pathway (Figure 8C). We speculate that this phenomenon could be explained by different BRCA subtypes represented by MDA-MB-231 and SKBR3 cells.

Last, we performed the IHC staining of BolA2B in pan-cancer tissue samples. Representative images of THCA, LIHC, READ, and BRCA samples are shown in Figure 8D. Essentially, it was observed that *BOLA2B* was highly expressed in tumor tissues, except for gastric cancer and LIHC. This suggests that *BOLA2B* may have different functions in different cancer types.

## 4 Discussion

*BOLA2B*, a member of the *BOLA* gene family, has been far less studied than many other genes. In this study, it was found that *BOLA2B* was highly expressed in a variety of human cancers, and its expression was correlated with immune infiltration (Zhang et al., 2021), (Wang et al., 2022), (Li et al., 2022). This high expression may be due to elevated CNVs. Nuttle et al. reported that the duplication of *BOLA2B* on chromosome 16p11.2 was a significant marker, differentiating humans from chimpanzees. Its copy number was shown to be correlated with both its RNA and protein expression. In addition, we investigated the role of *BOLA2B* in terms of epigenetic regulation. However, even though methylation has been found to be important in the development of some cancers (Su et al., 2017), *BOLA2B* methylation had little effect on the expression of the gene. As regards to immune infiltration, *BOLA2B* expression tended to be negatively correlated with both immune cells and immune-related molecules, including cytokines, except in some cancers. In addition, our results showed that *BOLA2B* expression did not correspond with “Immune Hot” or “Immune Cold” cancer.

It is documented that BolA1 is responsible for maintaining the spherical shape of *Escherichia coli* cells and is overexpressed under conditions of oxidative stress (Willems et al., 2013). Thus, BolA1 is an aerobic protein that protects the mitochondria through the depletion of GSH (Willems et al., 2013). Bioinformatic analysis revealed that the BolA protein family is closely connected with CGFS Grxs (Huynen et al., 2005). BolA proteins can form Fe–S-bridged complexes by exchanging with one Grx protein and GSH from Grx homodimers (Li and Outten, 2012; Rey et al., 2019). Although the BolA-Grx heterocomplex was less stable compared with the Grx Fe–S cluster, it could regulate iron homeostasis *in vivo*. In humans, BolA3 mutations result in multiple mitochondrial dysfunction syndromes associated with hyperglycinemia. Patients with BolA3 mutations were susceptible to various diseases and disorders, such as cardiomyopathy, abnormally high glycine levels in the serum, seizures, spasticity, and hypotonia (Cameron et al., 2011; Haack et al., 2013; Nikam et al., 2018; Talib and Outten, 2021). The BolA2 protein affects the iron-sensitive transcription factors AFT1 and AFT2 together with GRX3 and GRX4 (Talib and Outten, 2021). Specifically, BolA2 forms heterodimers with GRX3 and GRX4 through Fe–S bridges, while AFT2 monomers bind together to form dimers, thus reducing the affinity of AFT2 for binding to DNA and leading to failure of iron uptake (Poor et al., 2014; Li and Outten, 2019). In addition, BolA2 facilitated iron trafficking from cell cytosolic compartments to Fe–S cluster proteins. BolA2 functions together with Glrx3 as a [2Fe–2s] chaperone complex. Increasing iron concentrations result in an approximately 6–8-fold induction of the Glrx3-BolA2 complex, thus promoting the transfer of Fe–S clusters to apoproteins in human cells (Luo et al., 2019). Interestingly, Fe–S clusters formed by BolA2 were independent of Ciapin1, which could also bind with Glrx3 to assemble cytosolic Fe–S.

A recent study has shown that low CNVs of BolA2 might contribute to iron-deficiency anemia (Giannuzzi et al., 2019). BolA1, BolA2, and BolA3 are not classically secreted proteins (Zhou et al., 2008), and it is possible that the BolA family may have diverse functions. Several studies have shown that BolA proteins are associated with cell proliferation and cell cycle regulation (Kasai et al., 2004). In cancer, BolA2 and BolA3 were expressed at higher levels in ovarian cancer than in the normal adjacent tissue. The elevated expression of BolA1, BolA2, and BolA3 was significantly associated with the prognosis of ovarian cancer patients (Zhu and Xiao, 2021).

Some studies have proposed that BolA proteins function as transcriptional regulators (Santos et al., 2002; Dressaire et al., 2015). Mass spectrometry analysis has shown that there are four conserved phosphorylation sites in BolA proteins, namely, S26, S45, S95, and T81 (Vieira et al., 2004). S95 is located in the C-terminal domain, while others are located in the DNA-binding domain. Different types of phosphorylation or different combinations of phosphorylation would result in different effects. Phosphorylation at S26 and T81 would reduce the level of BolA proteins, while consistent phosphorylation at S45 and S95 would stabilize the protein, with dephosphorylation at these two sites, leading to a strong tendency for BolA to dimerize *in vitro* (Galego et al., 2021). BolA proteins are known to be involved in regulating cell morphology (Vieira et al., 2004; Freire et al., 2006). Impairing BolA phosphorylation at S26, S45, and T81 resulted in the development of rod-shaped *E coli* in contrast to the round WT cells. In *E. coli*, BolA was shown to bind directly to the mreB gene promoter region, thus controlling the shape of bacteria (Freire et al., 2009). MreB, a homolog of actin, is essential for cell extension and the maintenance of the rod shape (Wachi and Matsuhashi, 1989). BolA could also regulate biofilm formation in c-di-GMP. In BolA-deleted cells, the levels of c-di-GMP increased two-fold compared to the WT group (Moreira et al., 2017). c-di-GMP acts as a second messenger in bacteria and is responsible for cell motility, biofilm formation, cell differentiation, and cell cycle progression (Povolotsky and Hengge, 2012). In addition, BolA could reduce the expression of ydaM, a diguanylate cyclase involved in the synthesis of c-di-GMP. Overexpression of BolA induced the expression of yhjH, ydiV, yliE, and yahA, which are responsible for hydrolyzing c-di-GMP. c-di-GMP, in turn, could bind to the promoter region of BolA to regulate its transcription level (Moreira et al., 2017). Regarding cancer research, BolA2 was found highly expressed in hepatocellular carcinoma. Cox regression revealed that it was connected with tumor hemorrhage and worse HCC survival. These functions appeared to be performed through the activation of p62-Keap1 signaling (Luo et al., 2019).

In summary, the present study analyzed *BOLA2B* expression and its relationship with gene mutation, post-transcriptional modifications, and immune infiltration. It was also found that *BOLA2B* induced the G2/M cell-cycle arrest, thus reducing cancer cell proliferation. Our study provides new insights into this recently discovered gene and suggests that BolA2B may be a promising novel target for the treatment of various cancers.

## Data Availability

The original contributions presented in the study are included in the article/Supplementary Material; further inquiries can be directed to the corresponding authors.

## Supplementary material

SUPPLEMENTARY FIGURE S1 Effects of BOLA2B on disease-specific survival (DSS) analysis of different cancers. **(A)** Forest plot for hazard ratio and 95% confidence interval of BOLA2B on DSS across 38 cancers. **(B)** Representative images of BOLA2B influence on DSS prognosis of KIRC, GBMLGG, LGG, KIRP, KIRAN, THYM, LIHC, UVM, and OV.

SUPPLEMENTARY FIGURE S2 BOLA2B affected progression-free interval (PFI) analysis of different cancers. **(A)** Forest plot for hazard ratio and 95% confidence interval of BOLA2B on PFI across 38 cancers. **(B)** Representative images of BOLA2B influence on DSS prognosis of GBMLGG, LGG, KIRP, KIRAN, KIRC, LIHC, BLCA, SKCM-M, UVM, KICH, and OV.

SUPPLEMENTARY FIGURE S3 Immune score of BOLA2B in the top three cancers. **(A)** Relationship between ImmuneScore and BOLA2B expression in the top three cancers. **(B)** Relationship between StromalScore and BOLA2B expression in the top three cancers.

## Appendix A1:

**Table:**

Gene name
BOLA2B
GRX3
GRX4
TET2
NSUN5
ALYREF
KIAA1429
EDNRB
ENTPD1
TLR4
TP53
TTN
MUC16
AFT1
AFT2
mreB
ydaM
yhjH
ydiV
yliE
yahA

## Glossary

ACC: adrenocortical carcinoma
BRCA: breast-invasive carcinoma
CHOL: cholangiocarcinoma
COADREAD: colon adenocarcinoma/rectum adenocarcinoma esophageal carcinoma
ESCA: esophageal carcinoma
GBMLGG: glioma
KICH: kidney chromophobe
KIRC: kidney renal clear cell carcinoma
LAML: acute myeloid leukemia
LIHC: liver hepatocellular carcinoma
LUSC: lung squamous cell carcinoma
OV: ovarian serous cystadenocarcinoma
PCPG: pheochromocytoma and paraganglioma
READ: rectum adenocarcinoma
STAD: stomach adenocarcinoma
STES: stomach and esophageal carcinoma
THCA: thyroid carcinoma
UCEC: uterine corpus endometrial carcinoma
UVM: uveal melanoma
ALL: acute lymphoblastic leukemia
WT: high-risk Wilms tumor
DSS: disease-specific survival
TMB: tumor mutational burden
BLCA: bladder urothelial carcinoma
CESC: cervical squamous cell carcinoma and endocervical adenocarcinoma
COAD: colon adenocarcinoma
DLBC: lymphoid neoplasm diffuse large B-cell lymphoma
GBM: glioblastoma multiforme
HNSC: head and neck squamous cell carcinoma
KIPAN: pan-kidney cohort (KICH+KIRC+KIRP)
KIRP: kidney renal papillary cell carcinoma
LGG: Brain low-grade carcinoma
LUAD: lung adenocarcinoma
MESO: mesothelioma
PAAD: pancreatic adenocarcinoma
PRAD: prostate adenocarcinoma
SARC: sarcoma
SKCM: skin cutaneous carcinoma
TGCT: testicular germ cell tumors
THYM: thymoma
UCS: uterine carcinoma
OS: osteosarcoma
NB: neuroblastoma
OS: overall survival
PFI: progression-free interval
GC: gastric cancer
