# Supplementary figures and images for: Integrative analysis of the role of BOLA2B in human pan-cancer
Source: Front Genet. 2023 Feb 27;14:1077126. doi: 10.3389/fgene.2023.1077126 (PMC10008965; doi:10.3389/fgene.2023.1077126)

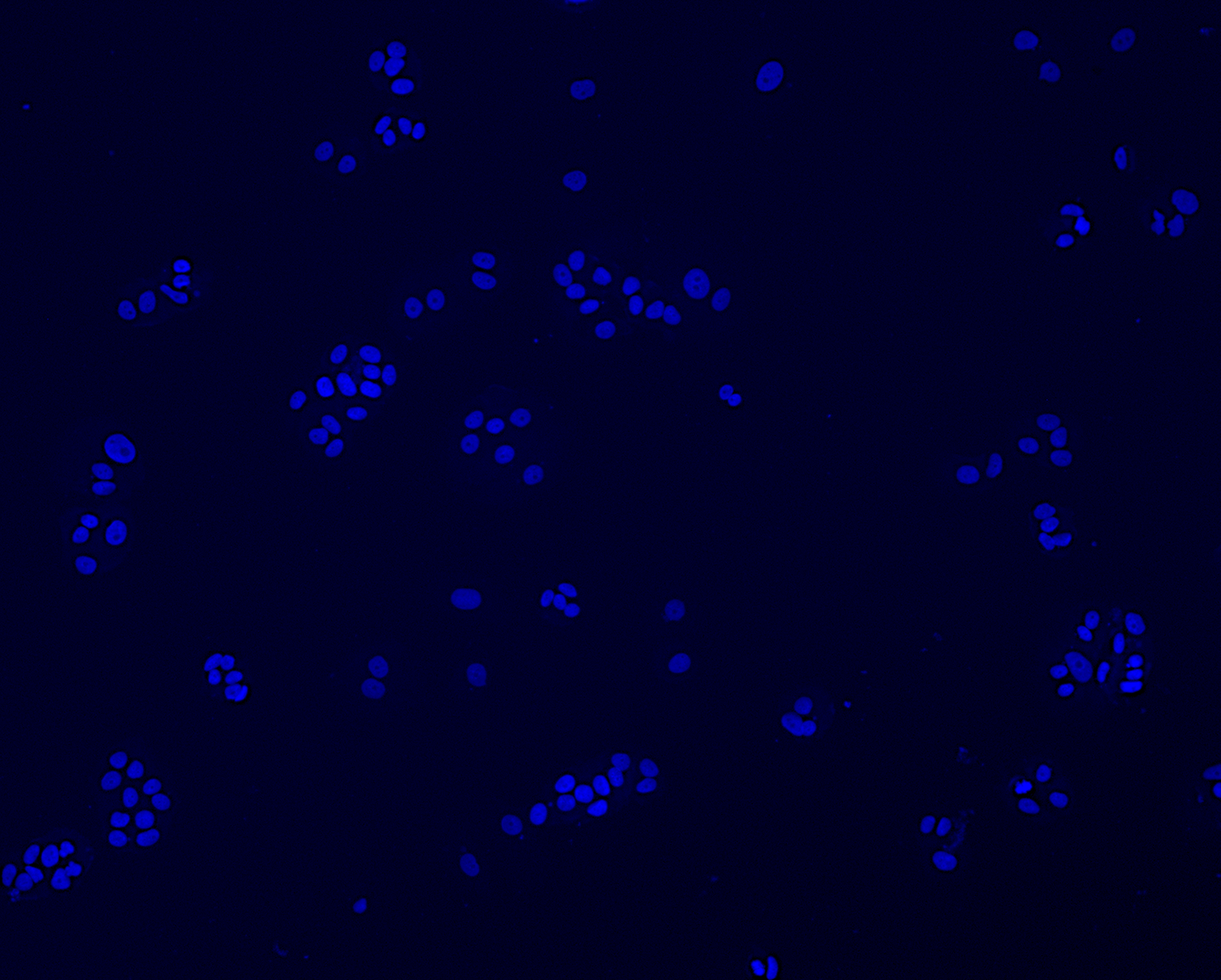

Supplement: Supplementary file 3 [file DataSheet8.ZIP › 拍摄-1288-图像导出-02.tif]

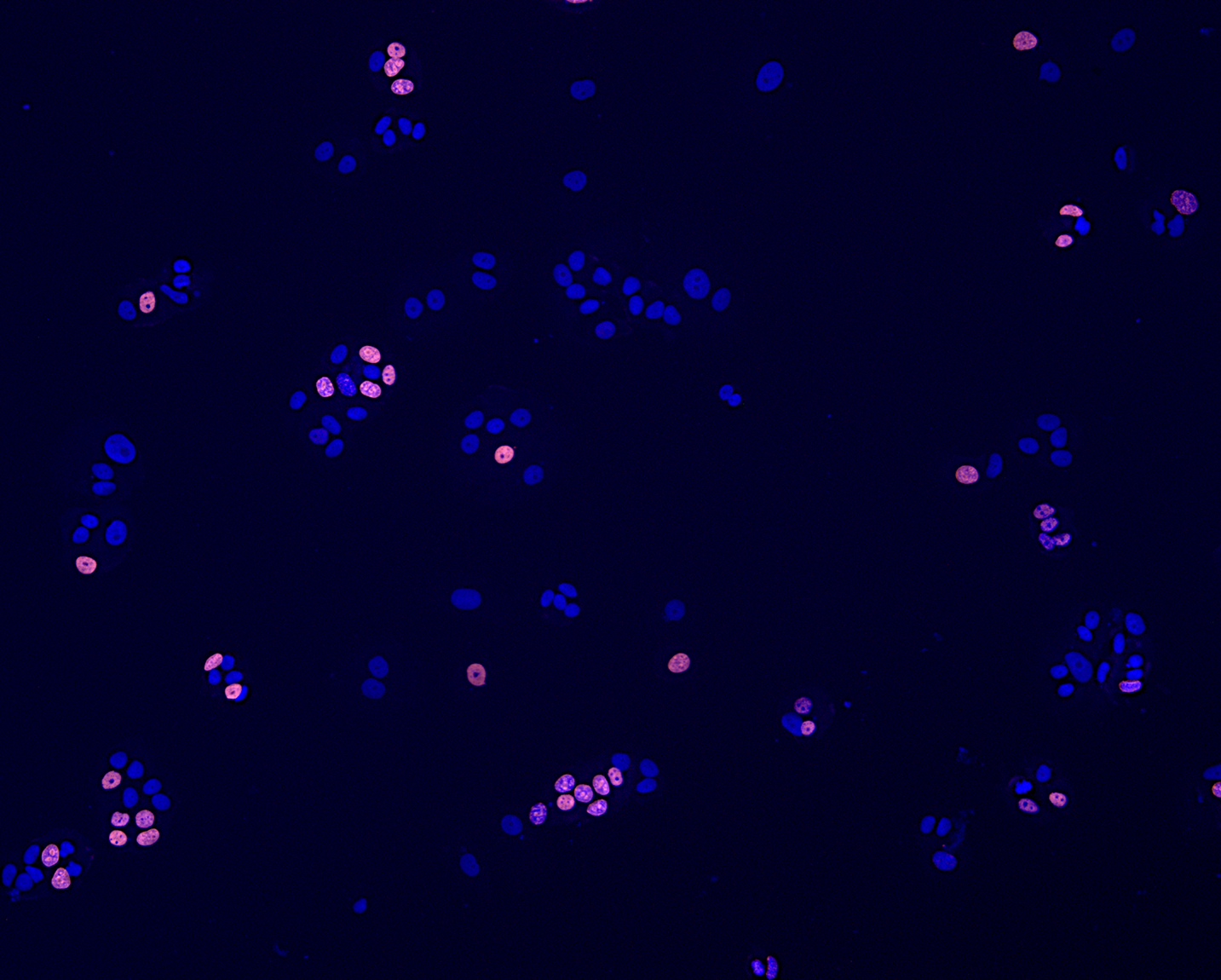

Supplement: Supplementary file 3 [file DataSheet8.ZIP › 拍摄-1288-添加通道-21-图像导出-03_c1+2.tif]

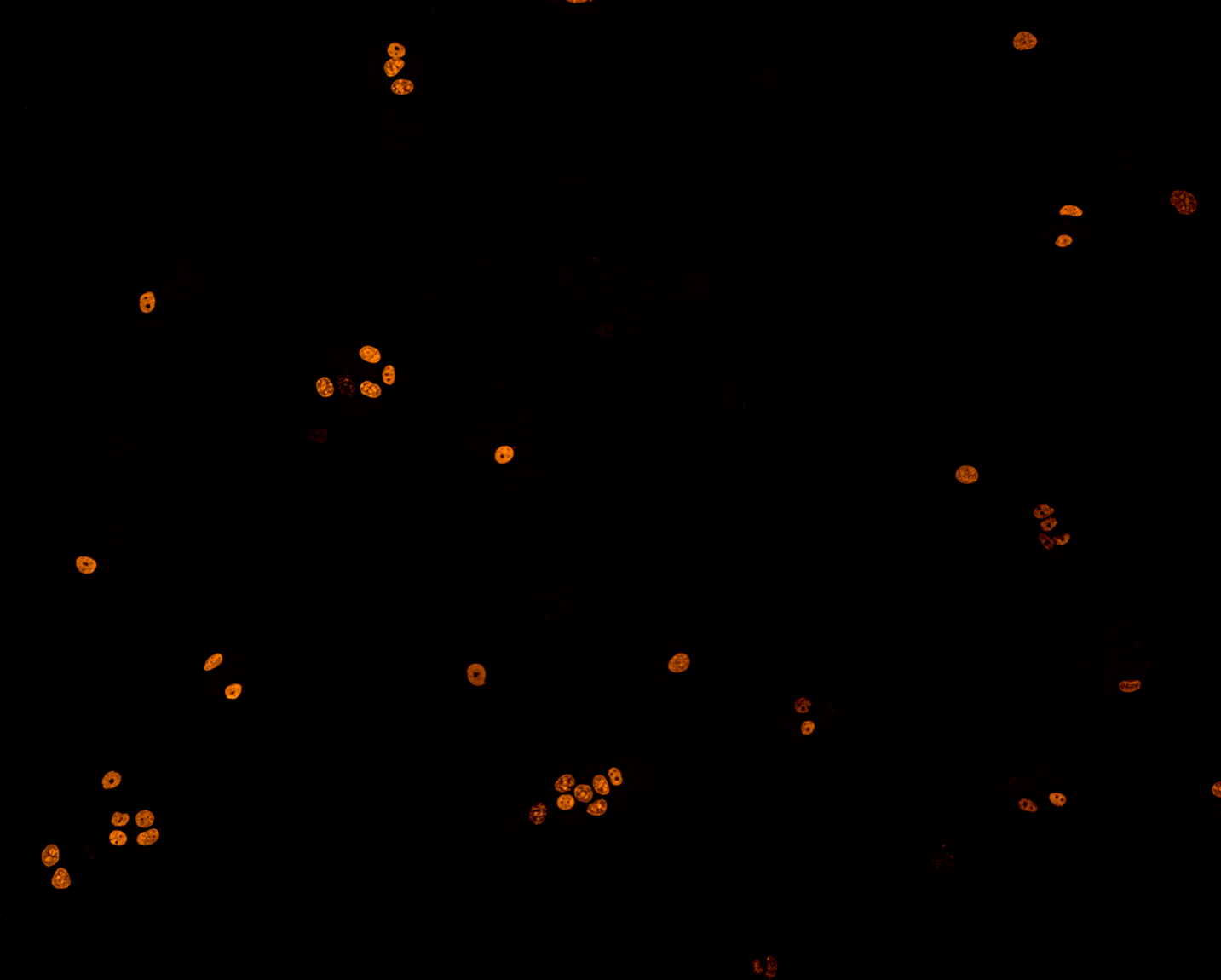

Supplement: Supplementary file 3 [file DataSheet8.ZIP › 拍摄-1289-图像导出-04.tif]

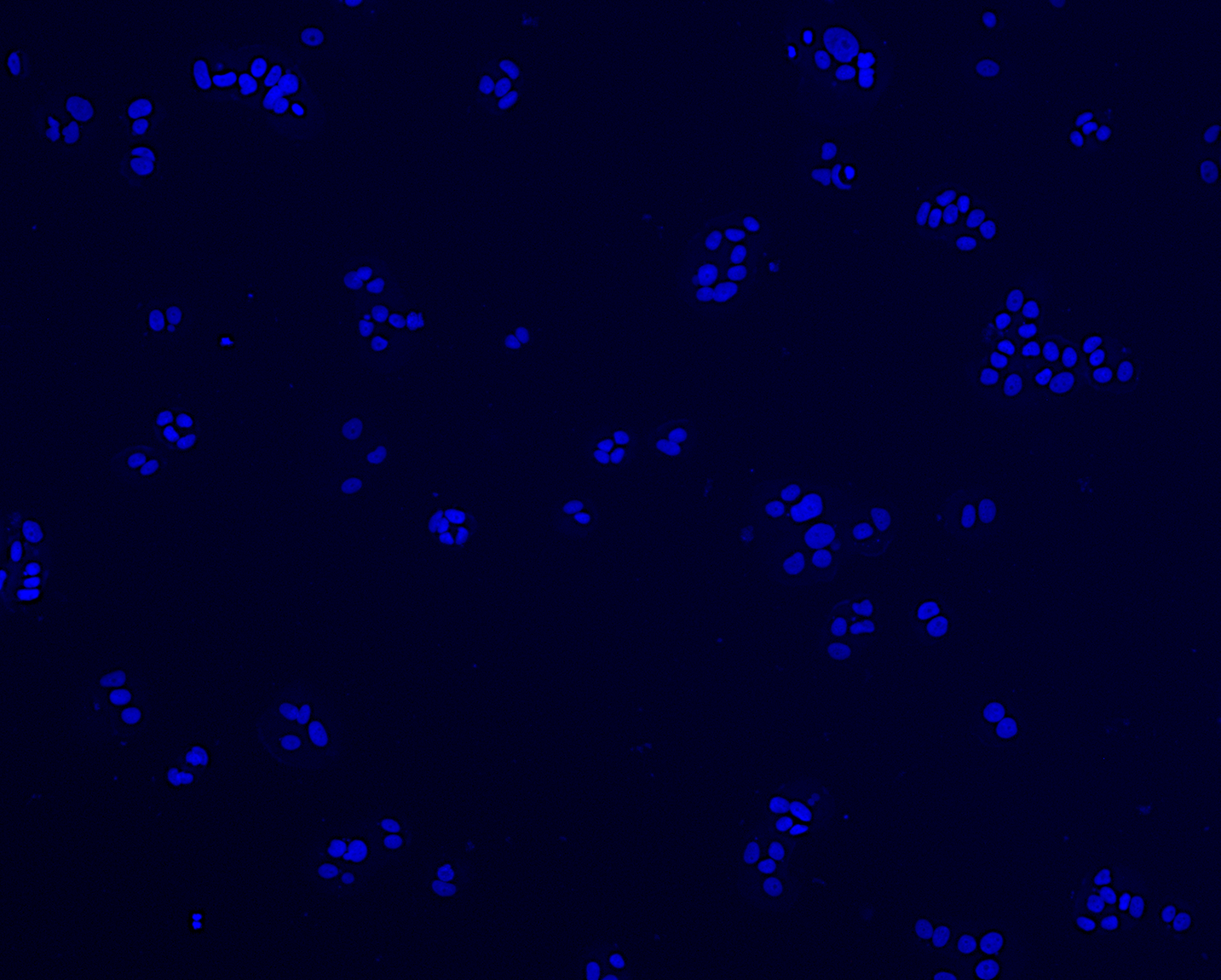

Supplement: Supplementary file 3 [file DataSheet8.ZIP › 拍摄-1290-图像导出-05.tif]

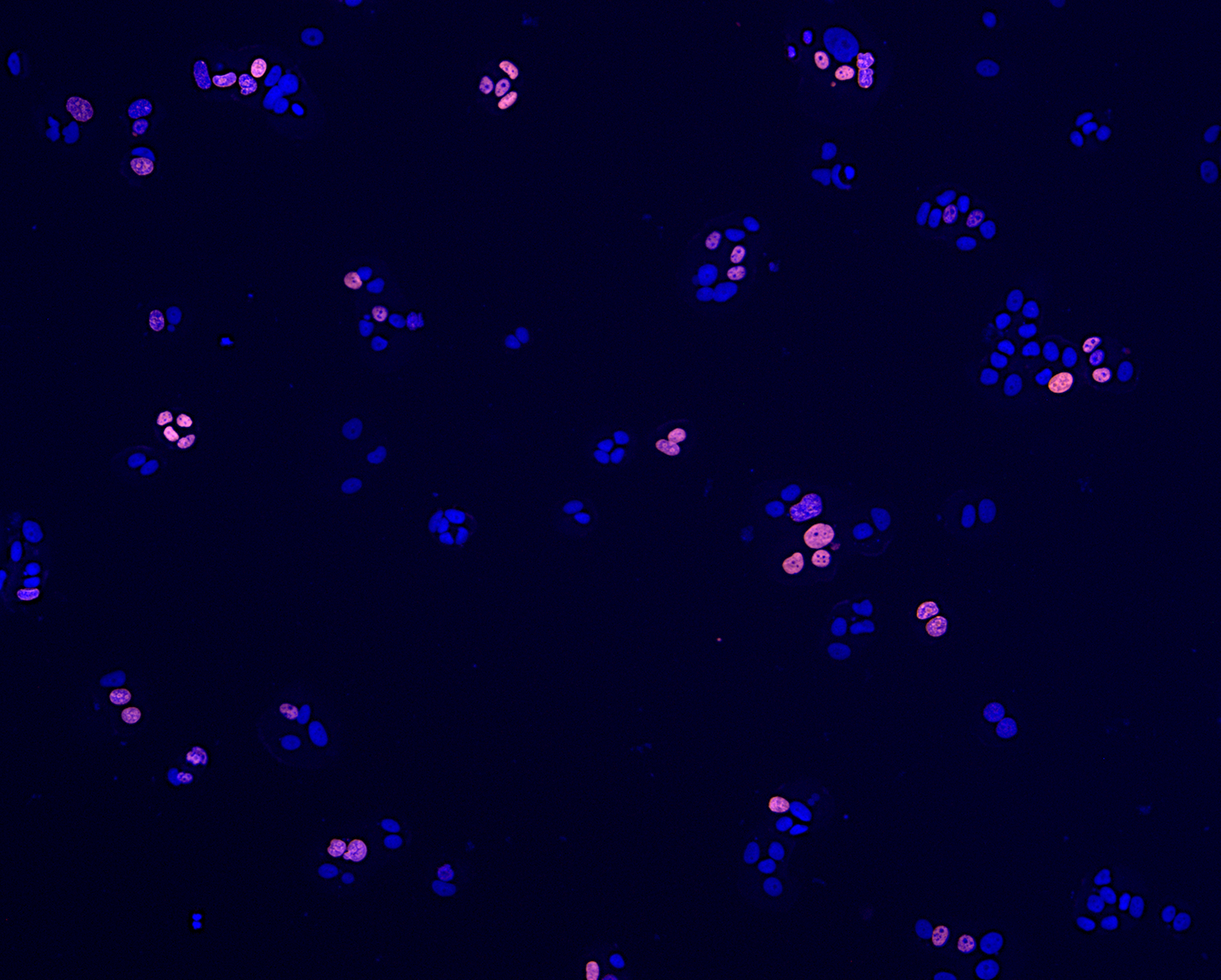

Supplement: Supplementary file 3 [file DataSheet8.ZIP › 拍摄-1290-添加通道-22-图像导出-06_c1+2.tif]

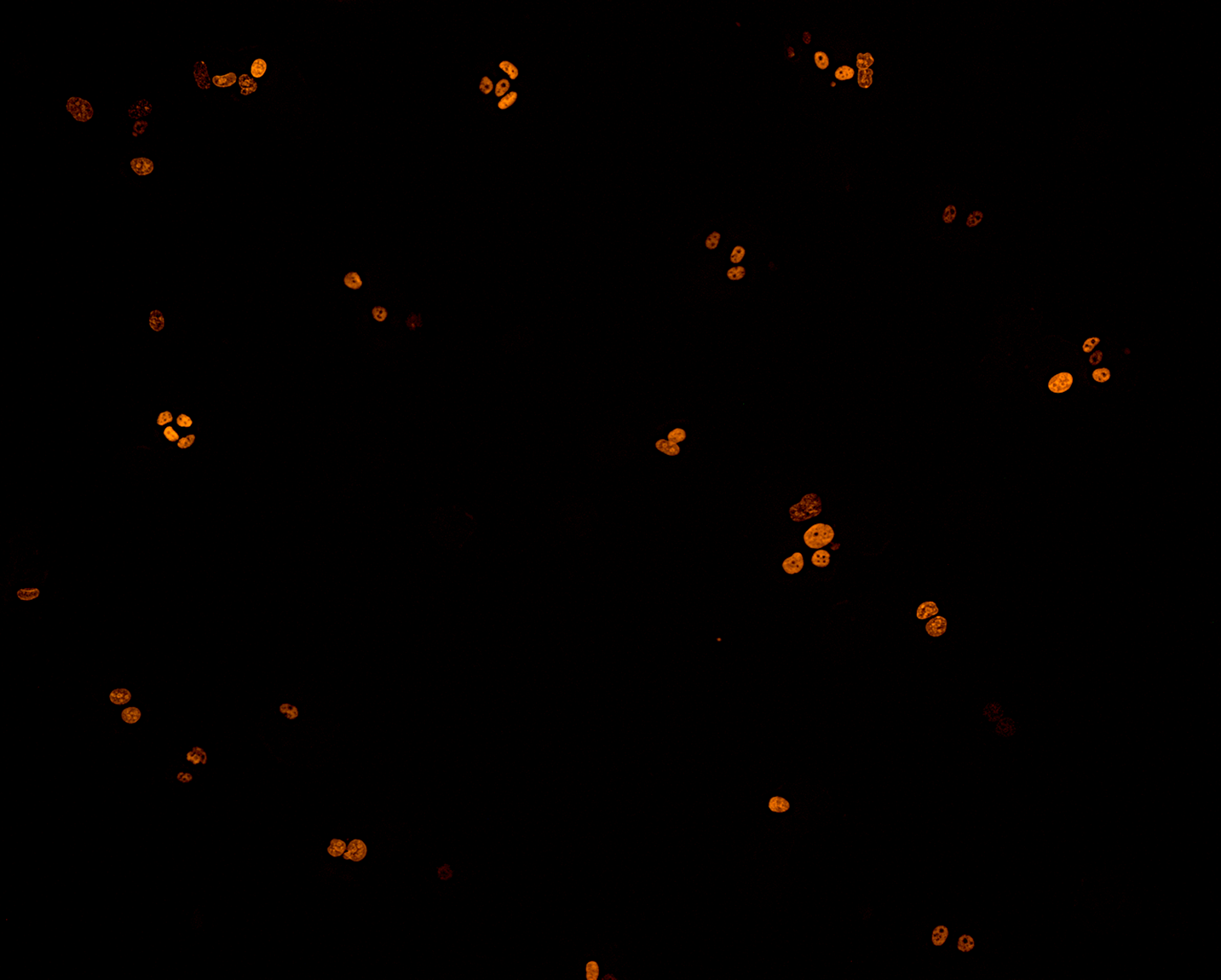

Supplement: Supplementary file 3 [file DataSheet8.ZIP › 拍摄-1291-图像导出-07.tif]

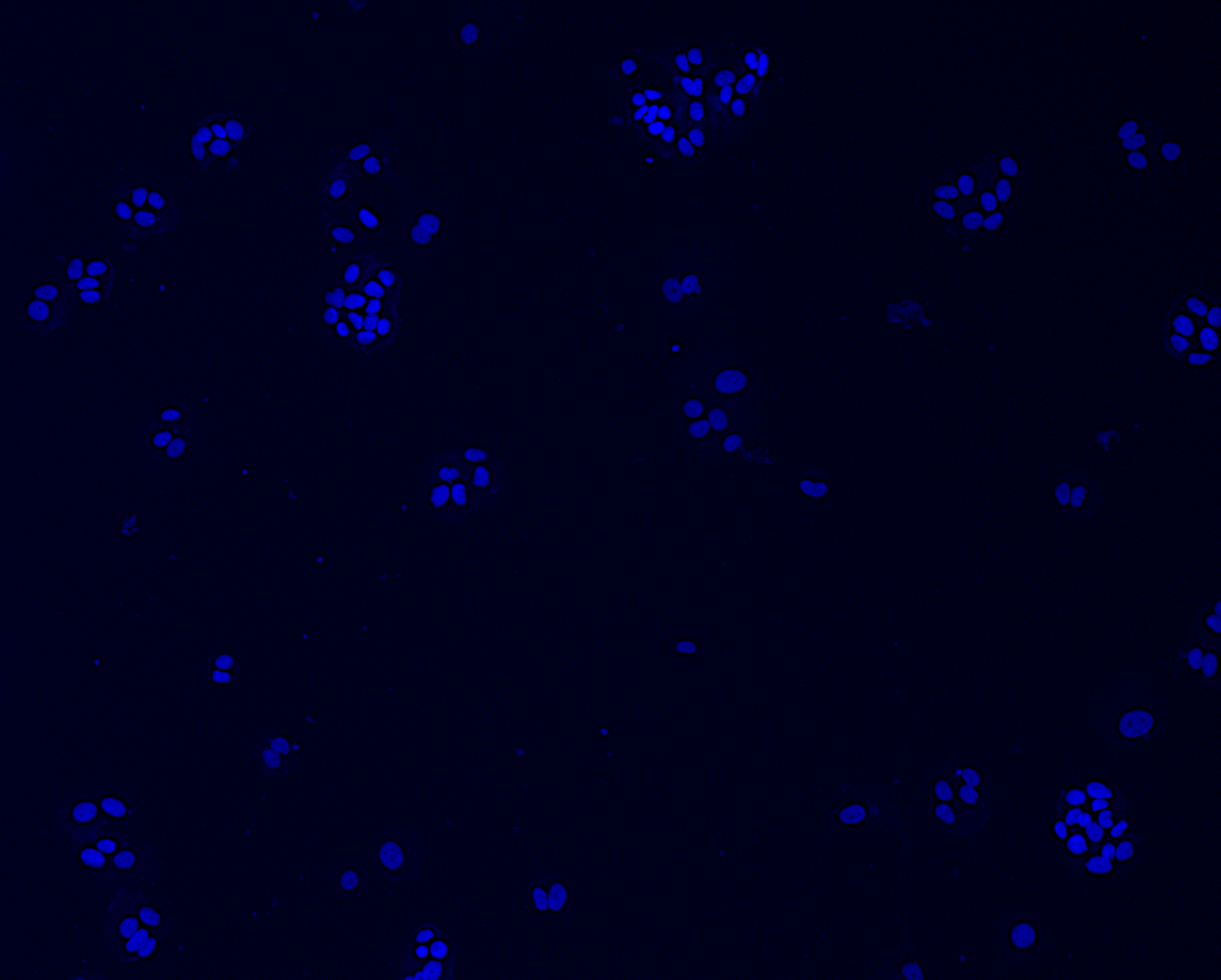

Supplement: Supplementary file 3 [file DataSheet8.ZIP › 拍摄-1292-图像导出-08.tif]

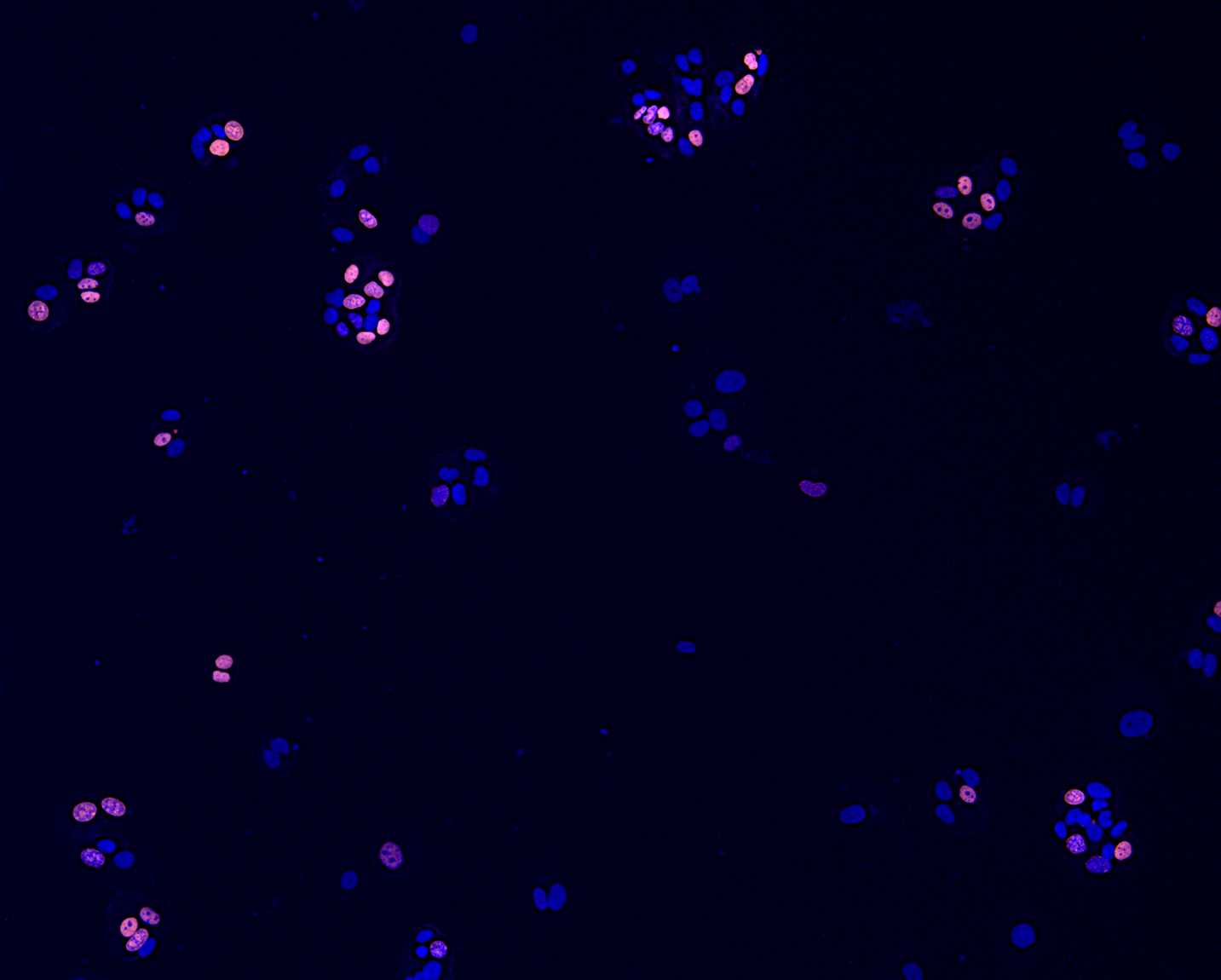

Supplement: Supplementary file 3 [file DataSheet8.ZIP › 拍摄-1292-添加通道-23-图像导出-09_c1+2.tif]

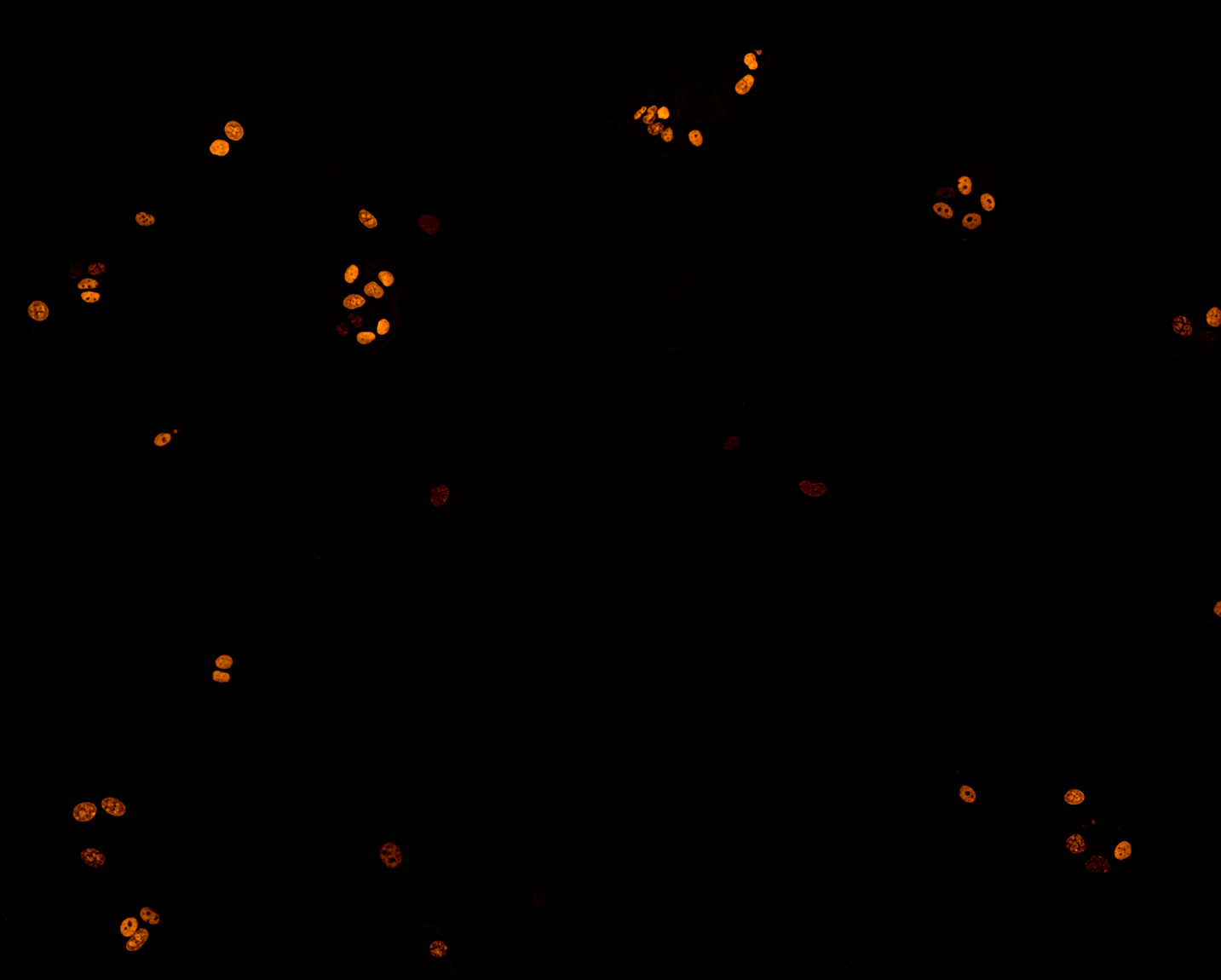

Supplement: Supplementary file 3 [file DataSheet8.ZIP › 拍摄-1293-图像导出-10.tif]

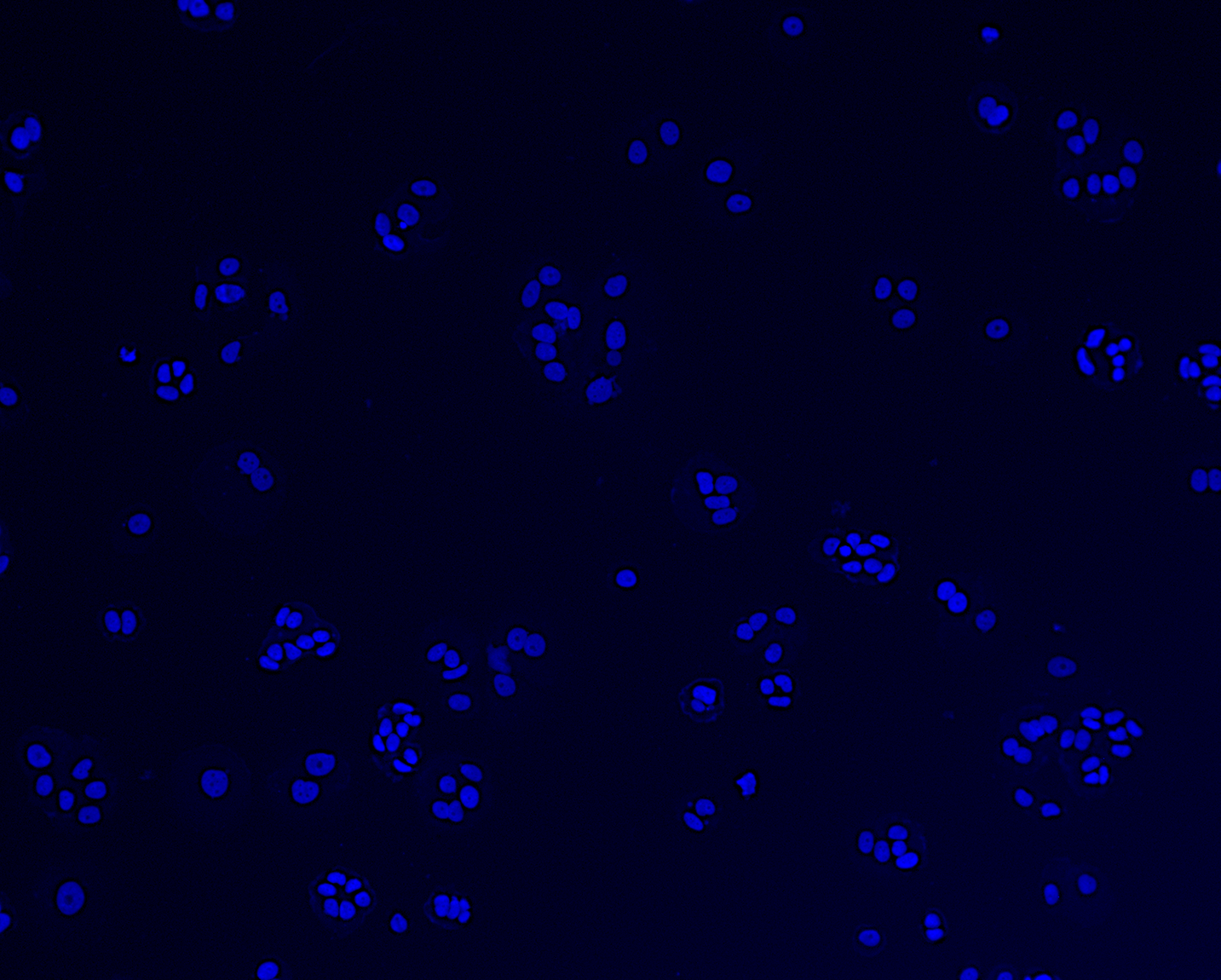

Supplement: Supplementary file 4 [file DataSheet9.ZIP › 拍摄-1310-图像导出-31.tif]

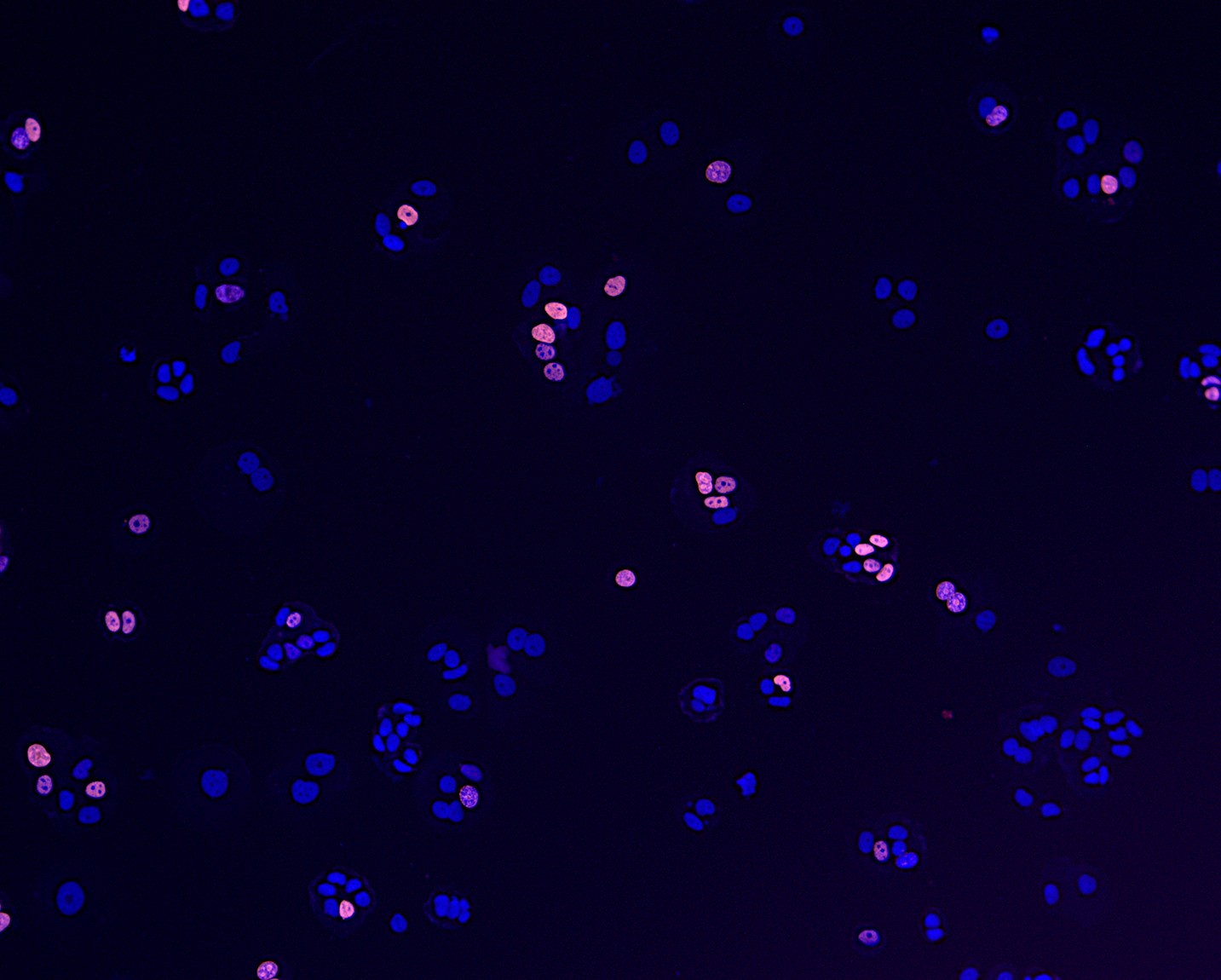

Supplement: Supplementary file 4 [file DataSheet9.ZIP › 拍摄-1310-添加通道-31-图像导出-32_c1+2.tif]

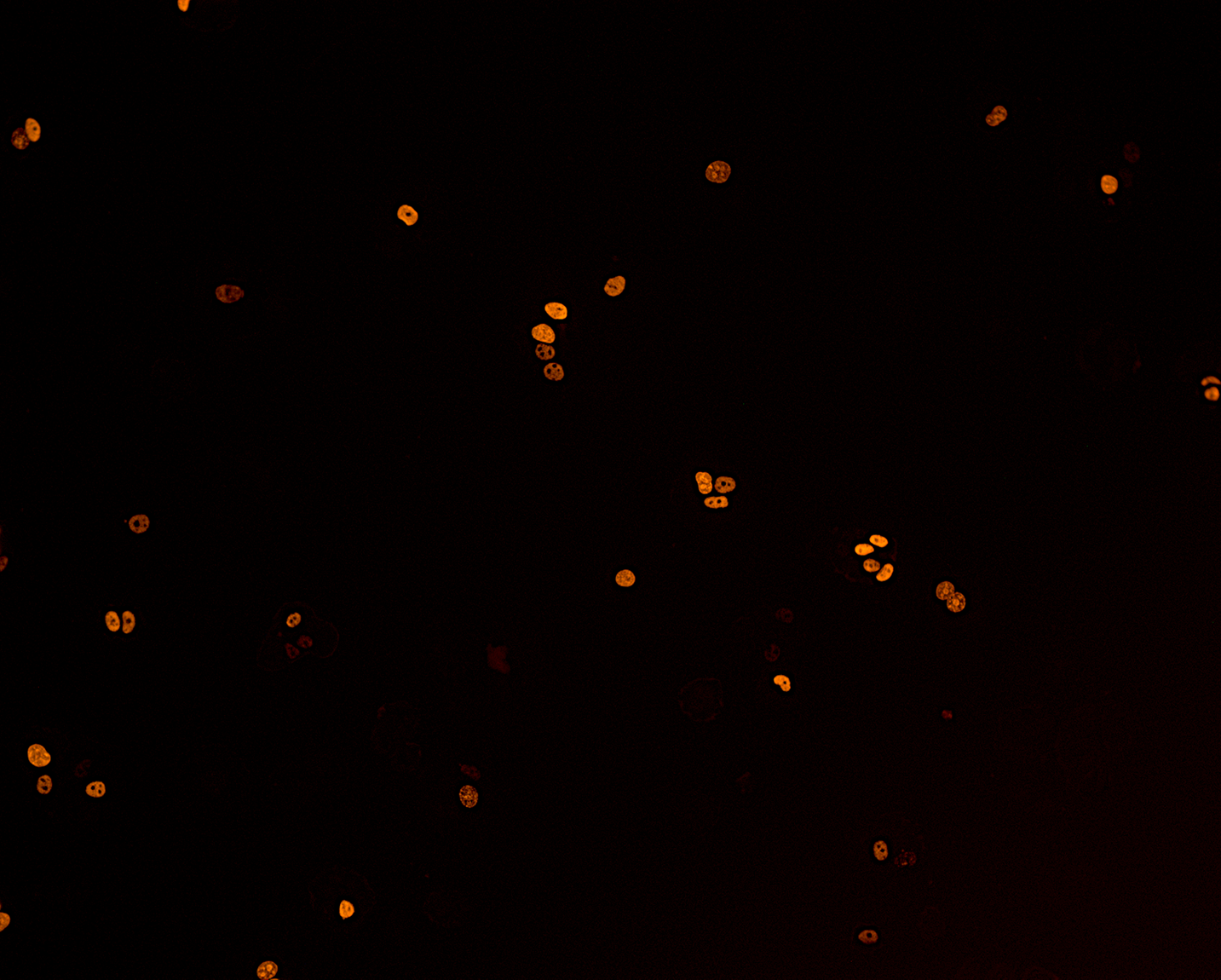

Supplement: Supplementary file 4 [file DataSheet9.ZIP › 拍摄-1311-图像导出-33.tif]

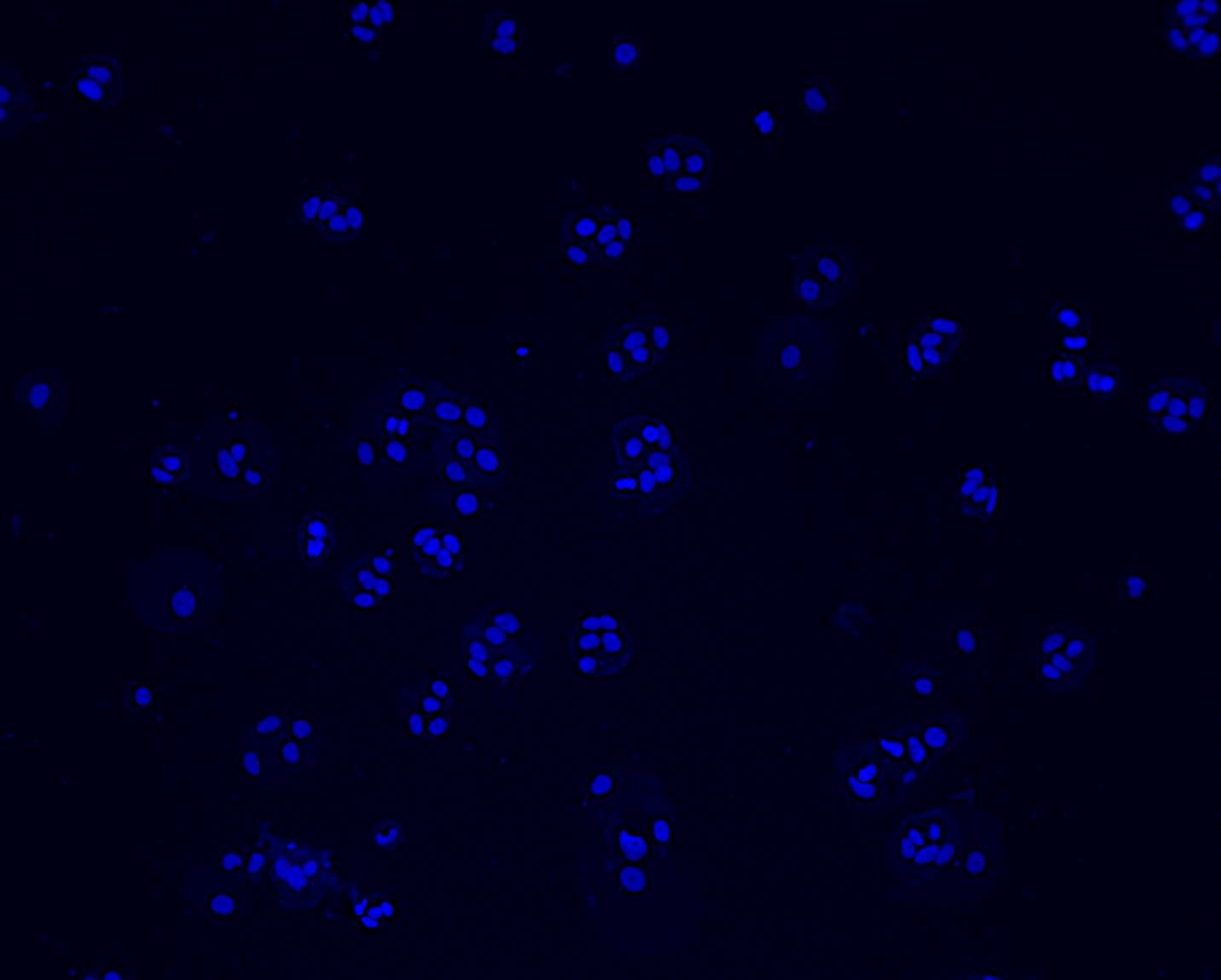

Supplement: Supplementary file 4 [file DataSheet9.ZIP › 拍摄-1312-图像导出-34.tif]

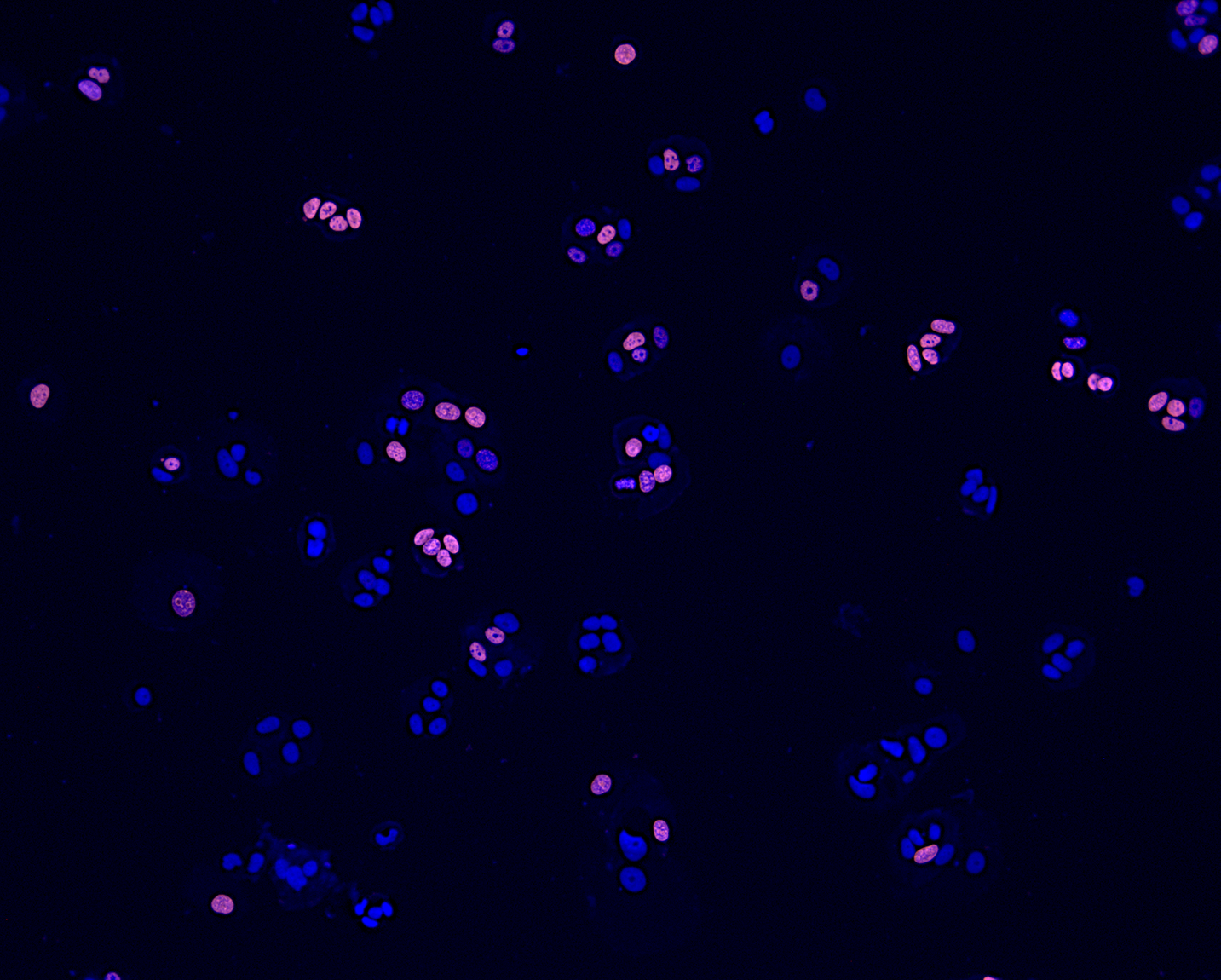

Supplement: Supplementary file 4 [file DataSheet9.ZIP › 拍摄-1312-添加通道-32-图像导出-35_c1+2.tif]

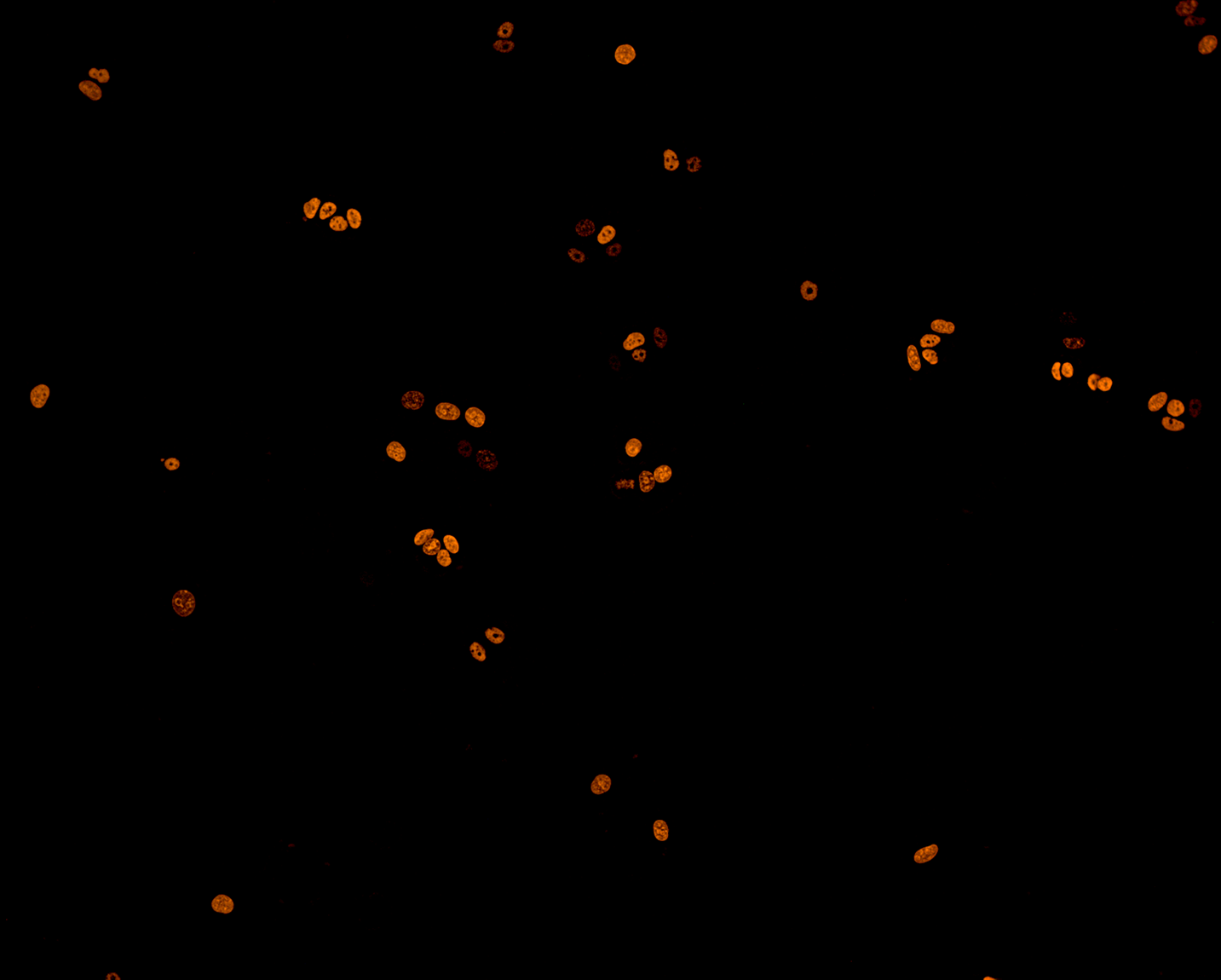

Supplement: Supplementary file 4 [file DataSheet9.ZIP › 拍摄-1313-图像导出-36.tif]

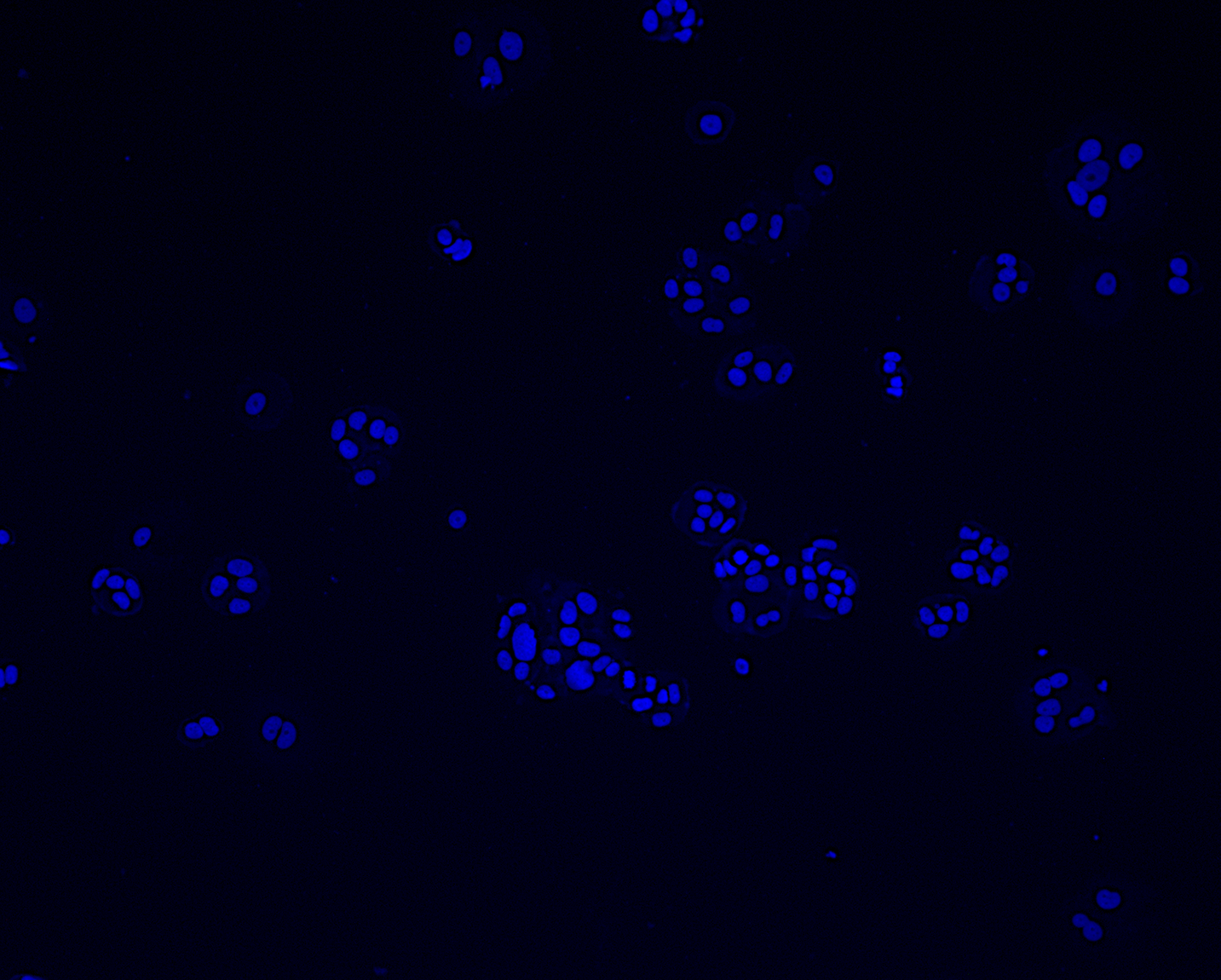

Supplement: Supplementary file 4 [file DataSheet9.ZIP › 拍摄-1314-图像导出-37.tif]

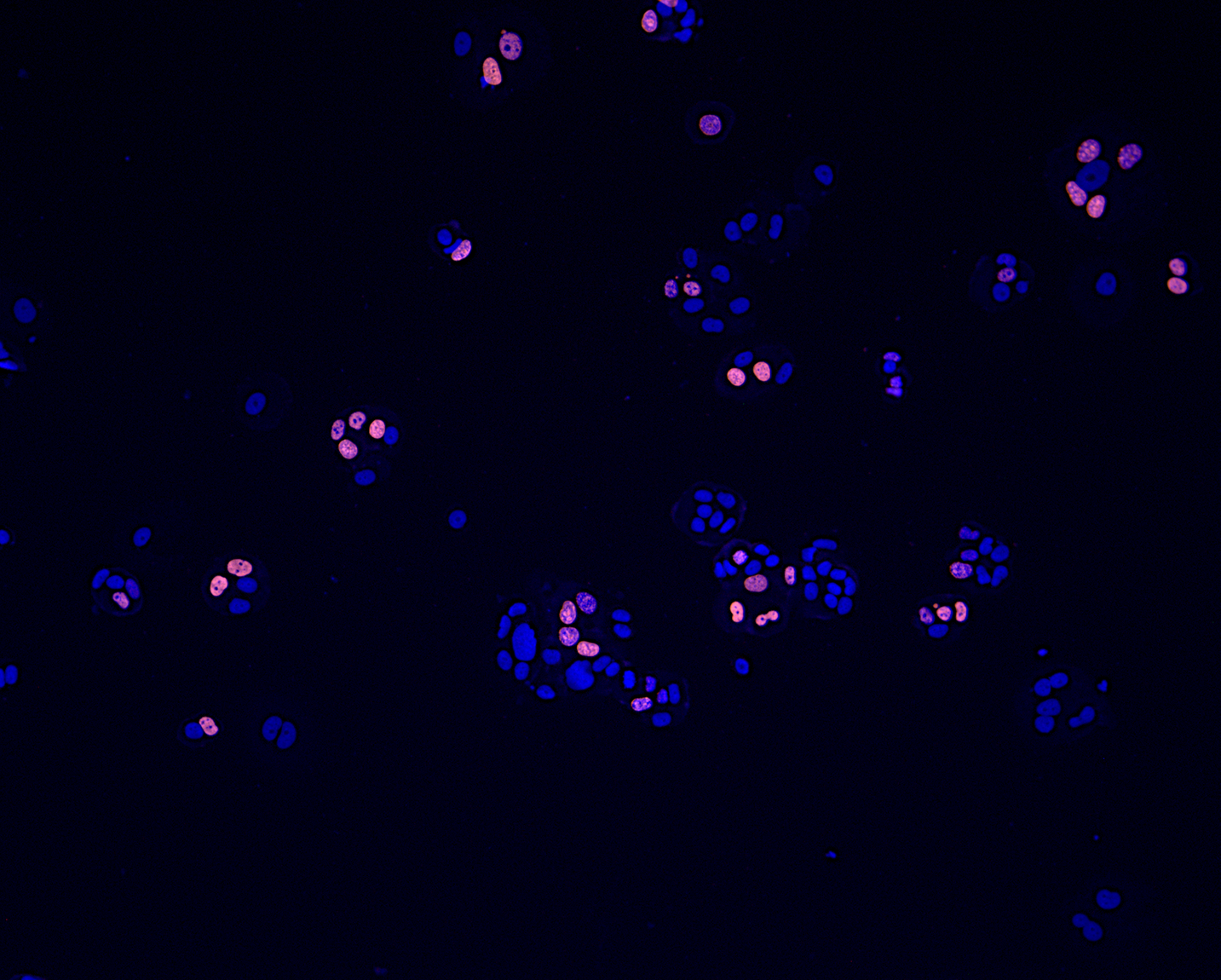

Supplement: Supplementary file 4 [file DataSheet9.ZIP › 拍摄-1314-添加通道-33-图像导出-38_c1+2.tif]

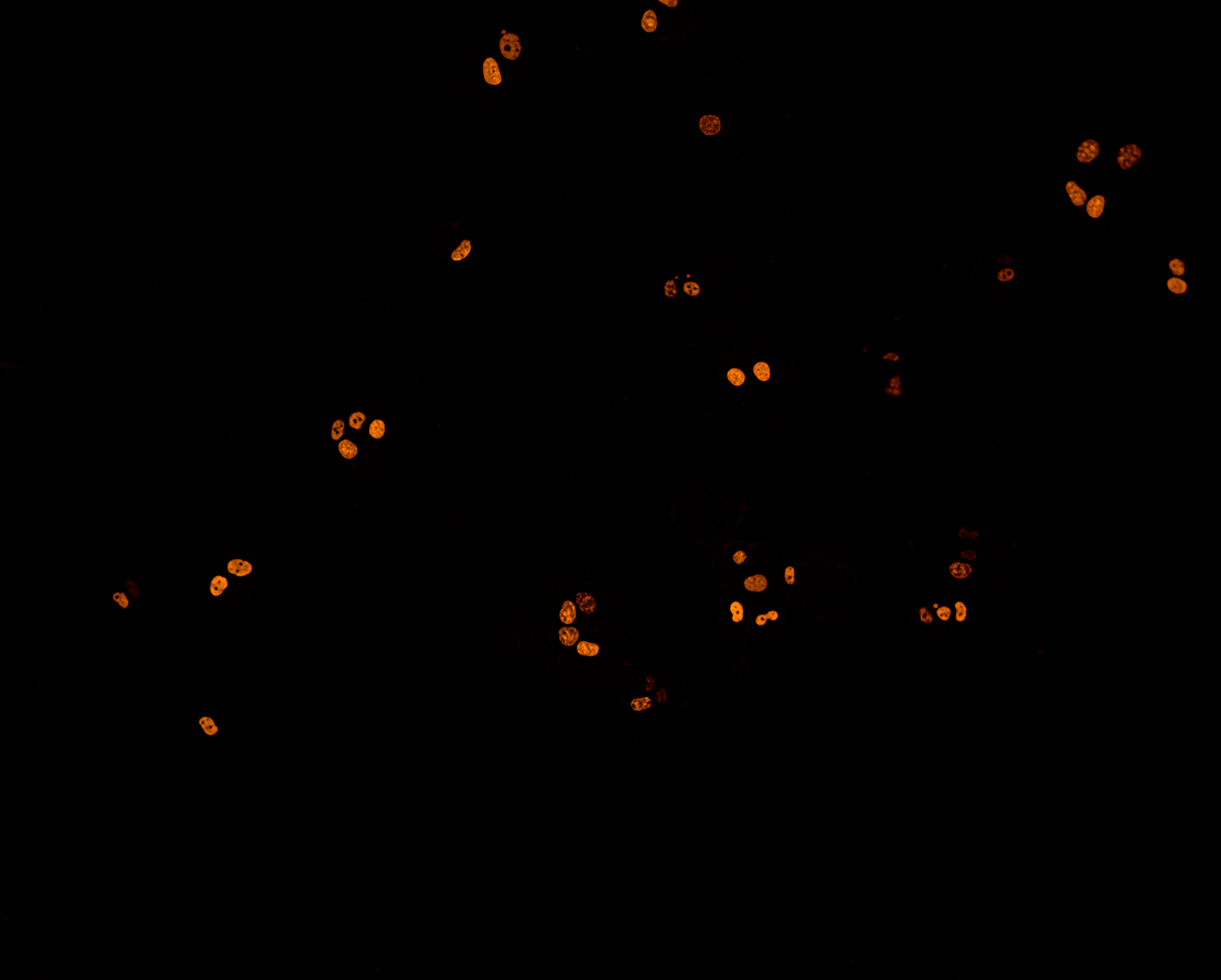

Supplement: Supplementary file 4 [file DataSheet9.ZIP › 拍摄-1315-图像导出-39.tif]

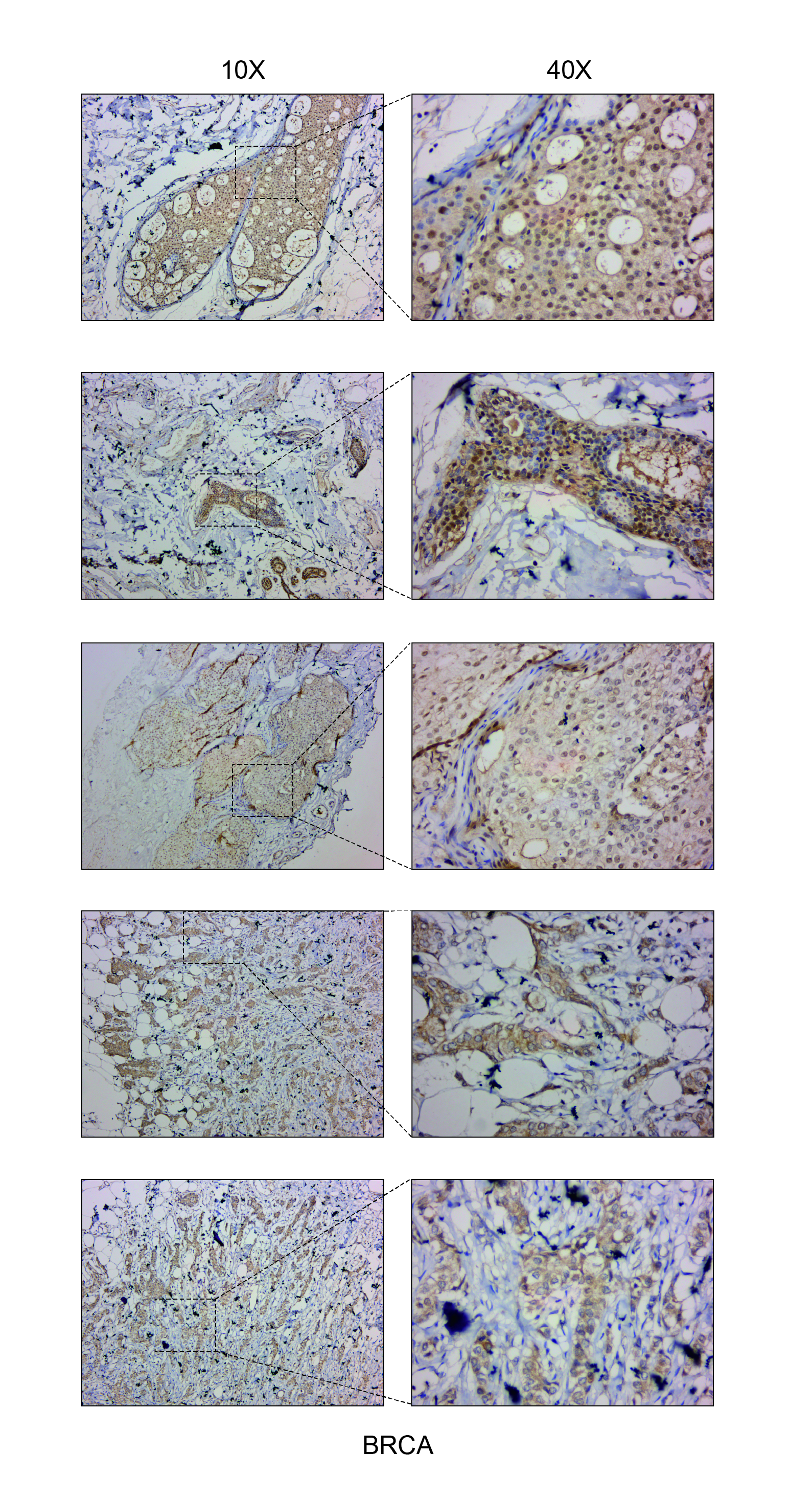

Supplement: Supplementary file 5 [file Image6.TIF]

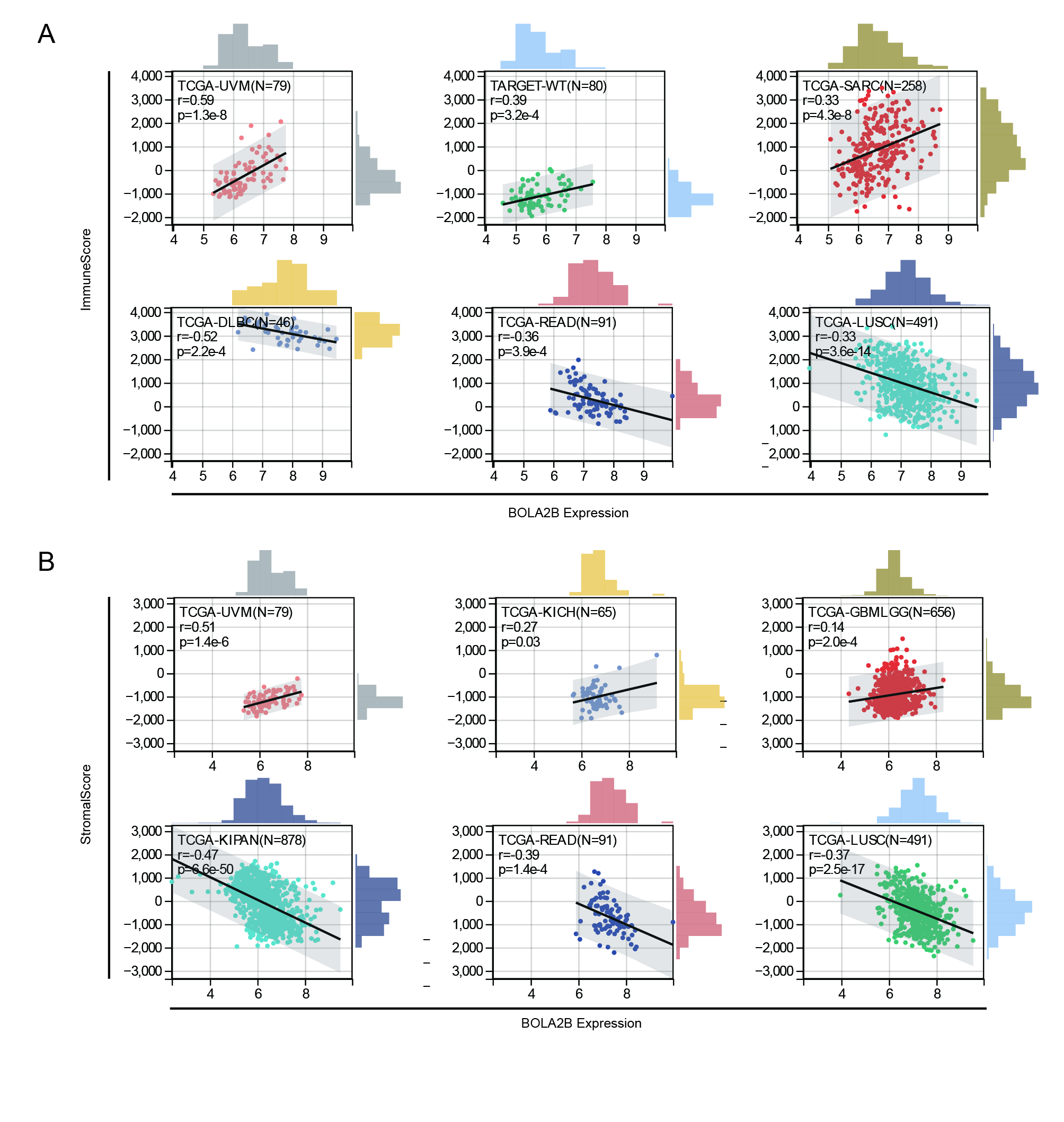

Supplement: Supplementary file 7 [file Image3.TIF]

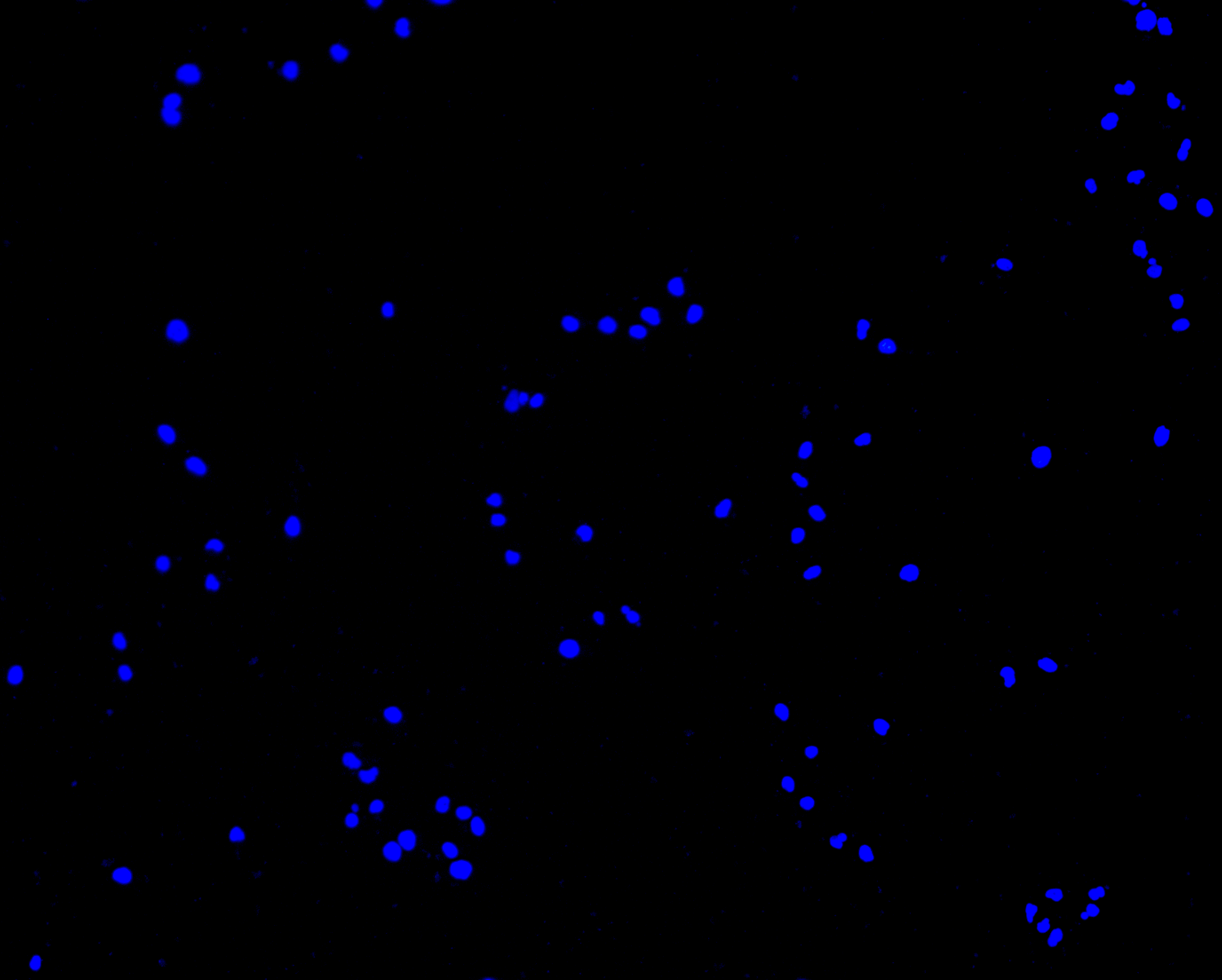

Supplement: Supplementary file 8 [file DataSheet4.ZIP › 拍摄-1179-图像导出-01.tif]

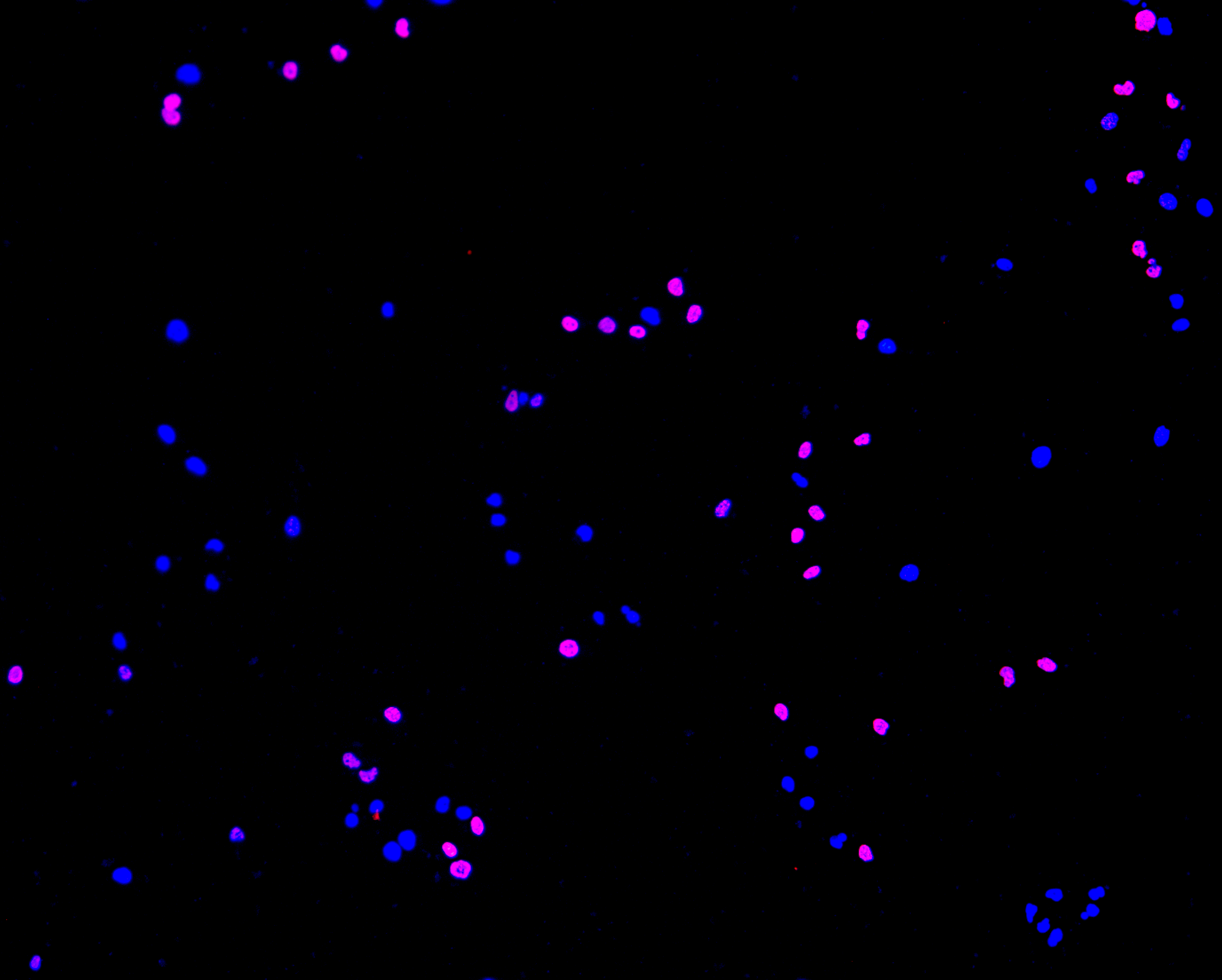

Supplement: Supplementary file 8 [file DataSheet4.ZIP › 拍摄-1179-添加通道-45-图像导出-02_c1+2.tif]

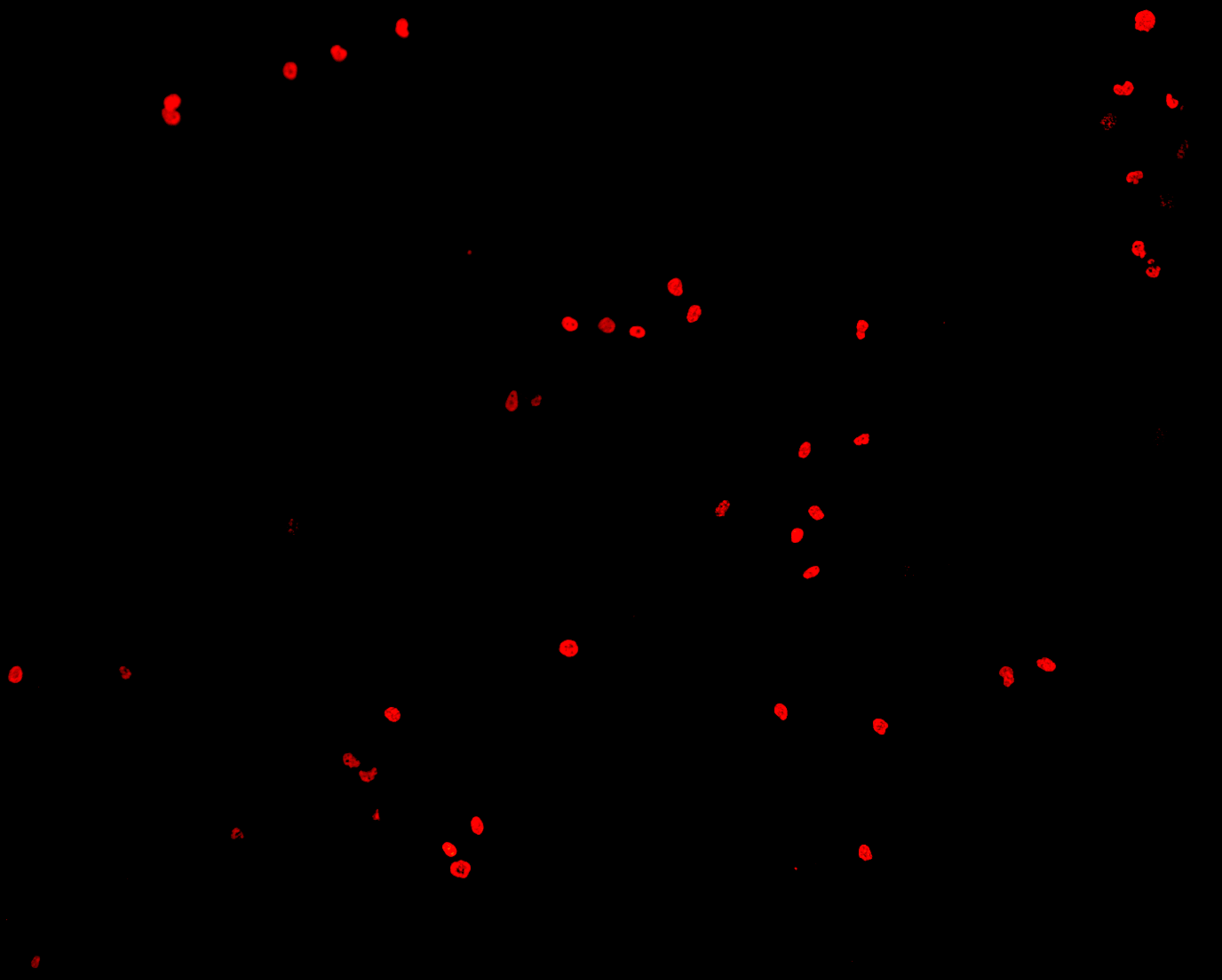

Supplement: Supplementary file 8 [file DataSheet4.ZIP › 拍摄-1180-图像导出-03.tif]

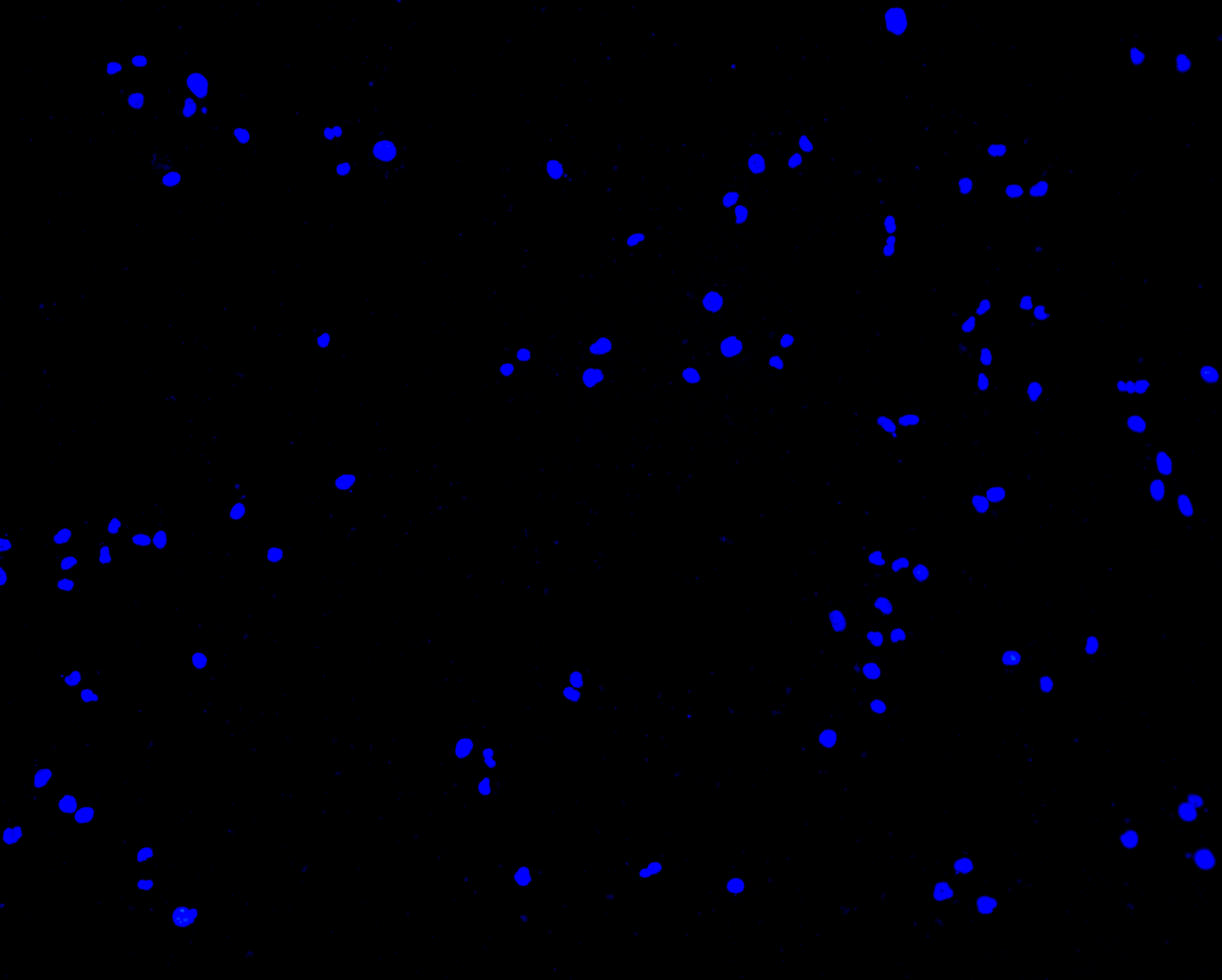

Supplement: Supplementary file 8 [file DataSheet4.ZIP › 拍摄-1181-图像导出-04.tif]

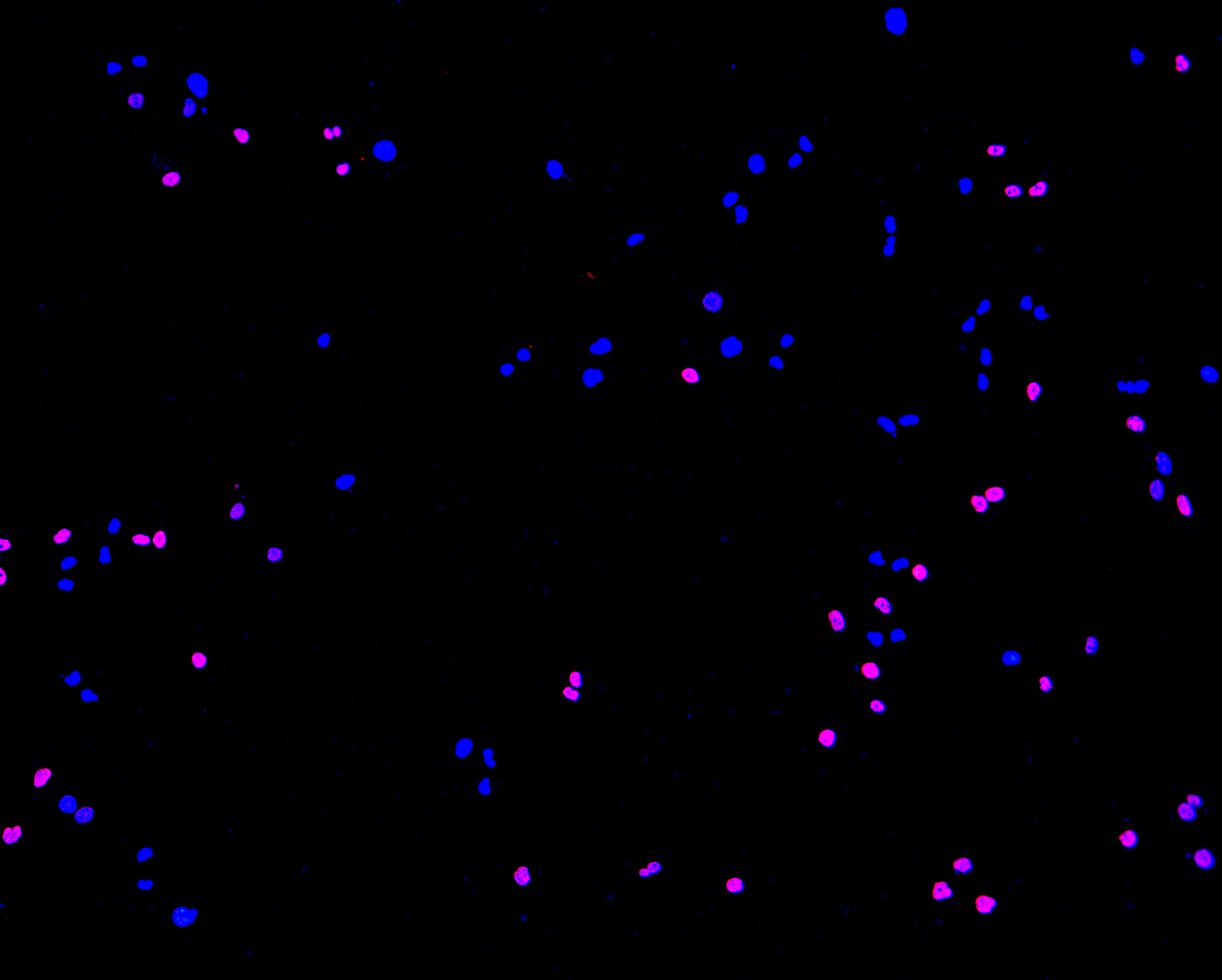

Supplement: Supplementary file 8 [file DataSheet4.ZIP › 拍摄-1181-添加通道-46-图像导出-05_c1+2.tif]

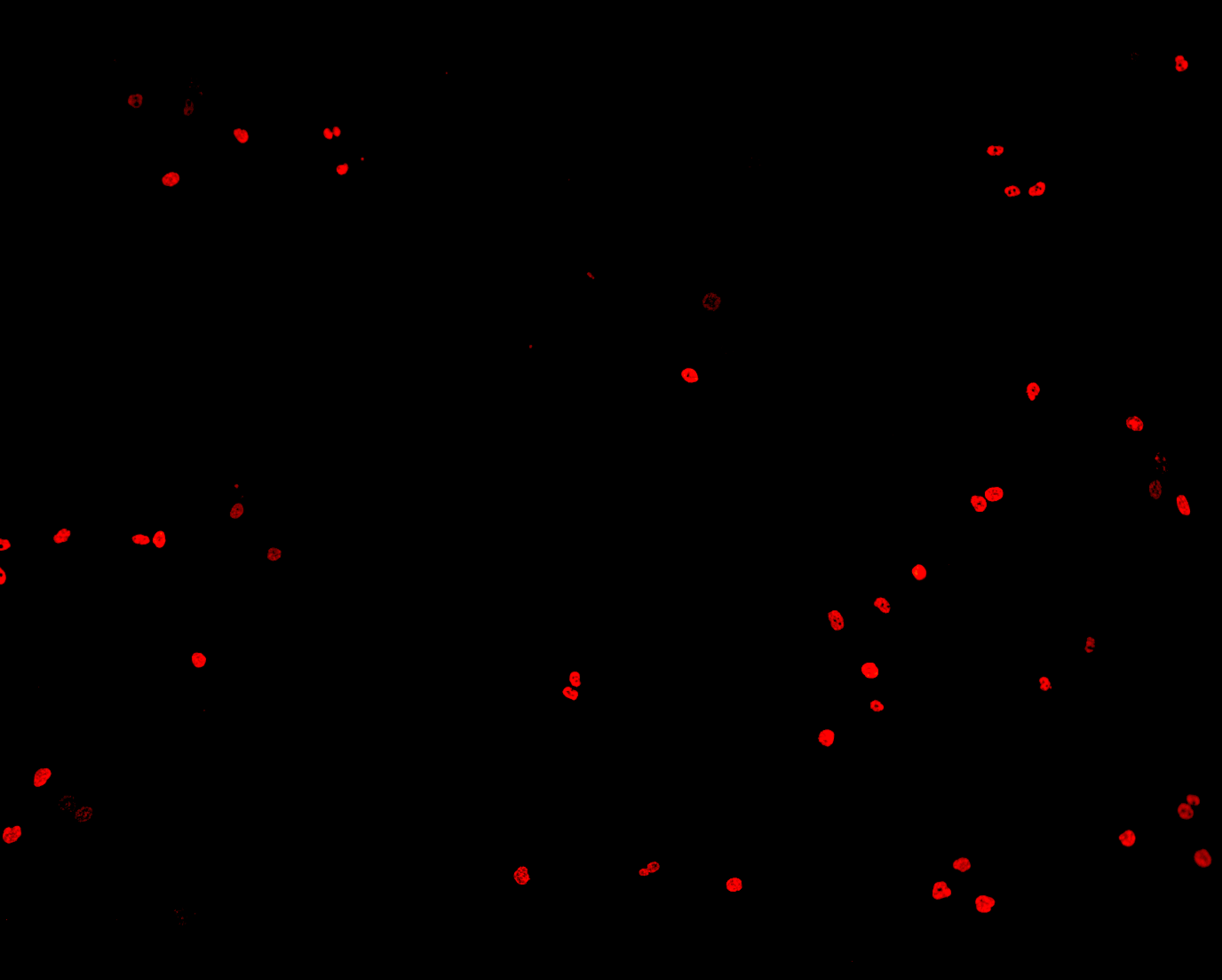

Supplement: Supplementary file 8 [file DataSheet4.ZIP › 拍摄-1182-图像导出-06.tif]

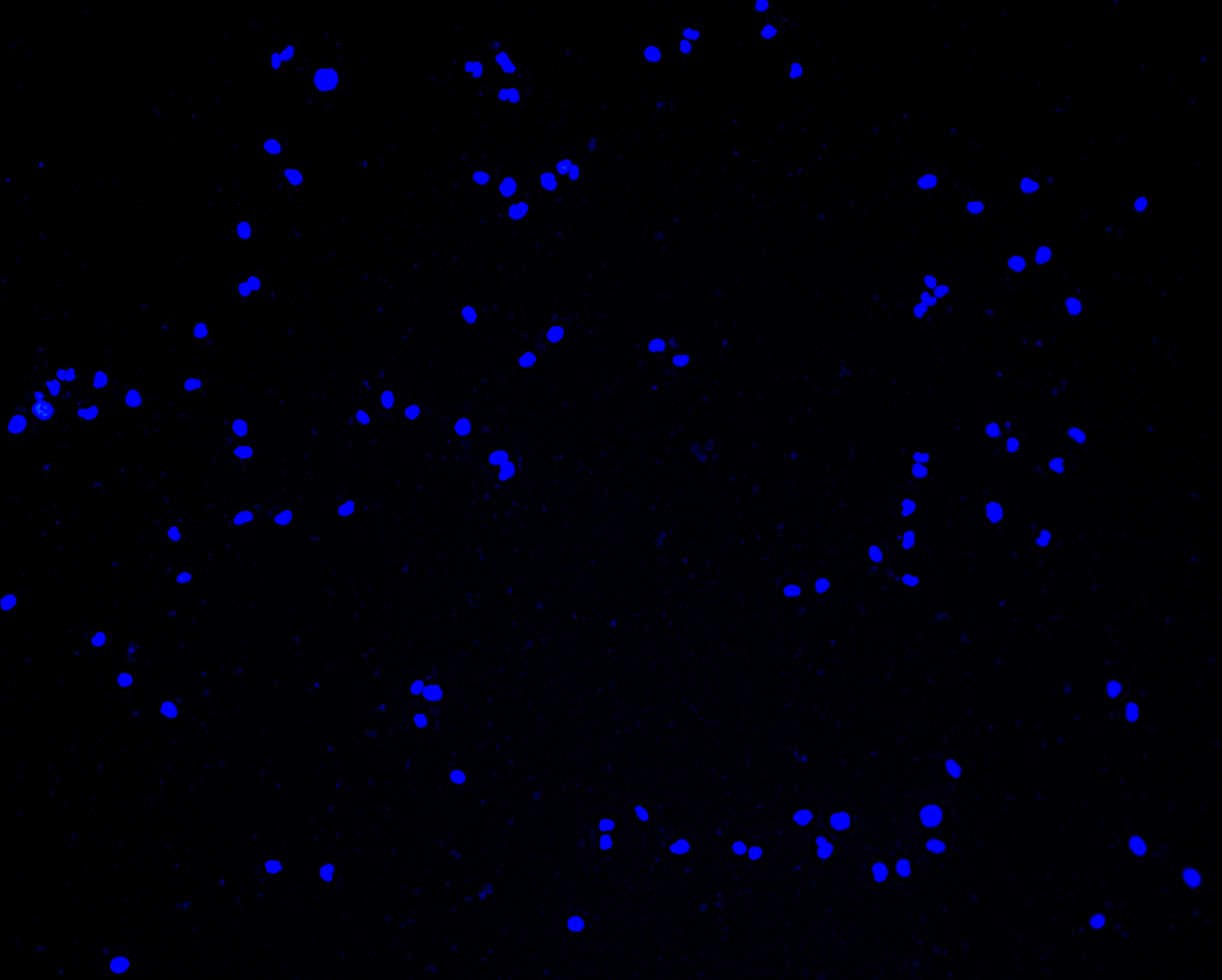

Supplement: Supplementary file 8 [file DataSheet4.ZIP › 拍摄-1189-图像导出-16.tif]

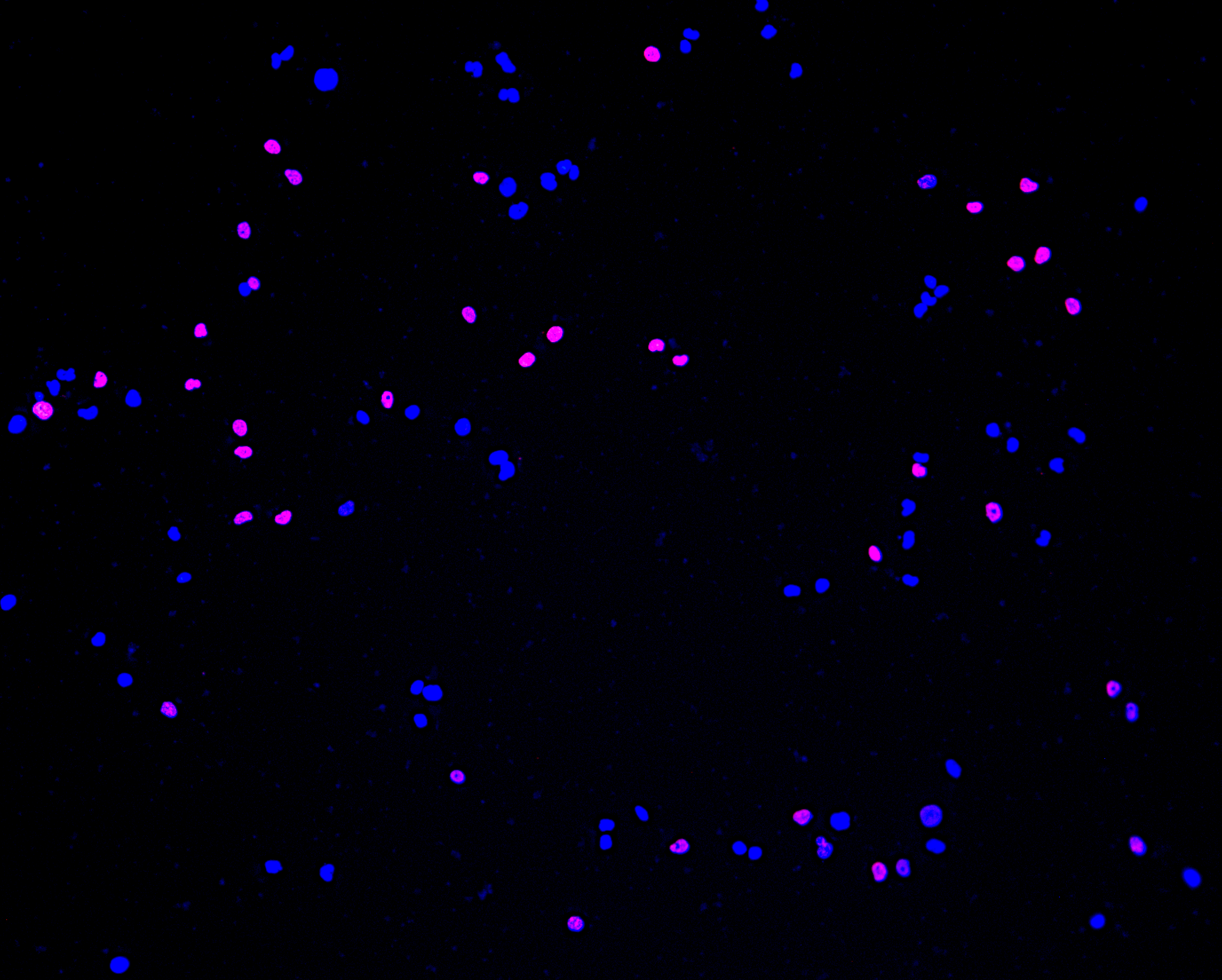

Supplement: Supplementary file 8 [file DataSheet4.ZIP › 拍摄-1189-添加通道-50-图像导出-17_c1+2.tif]

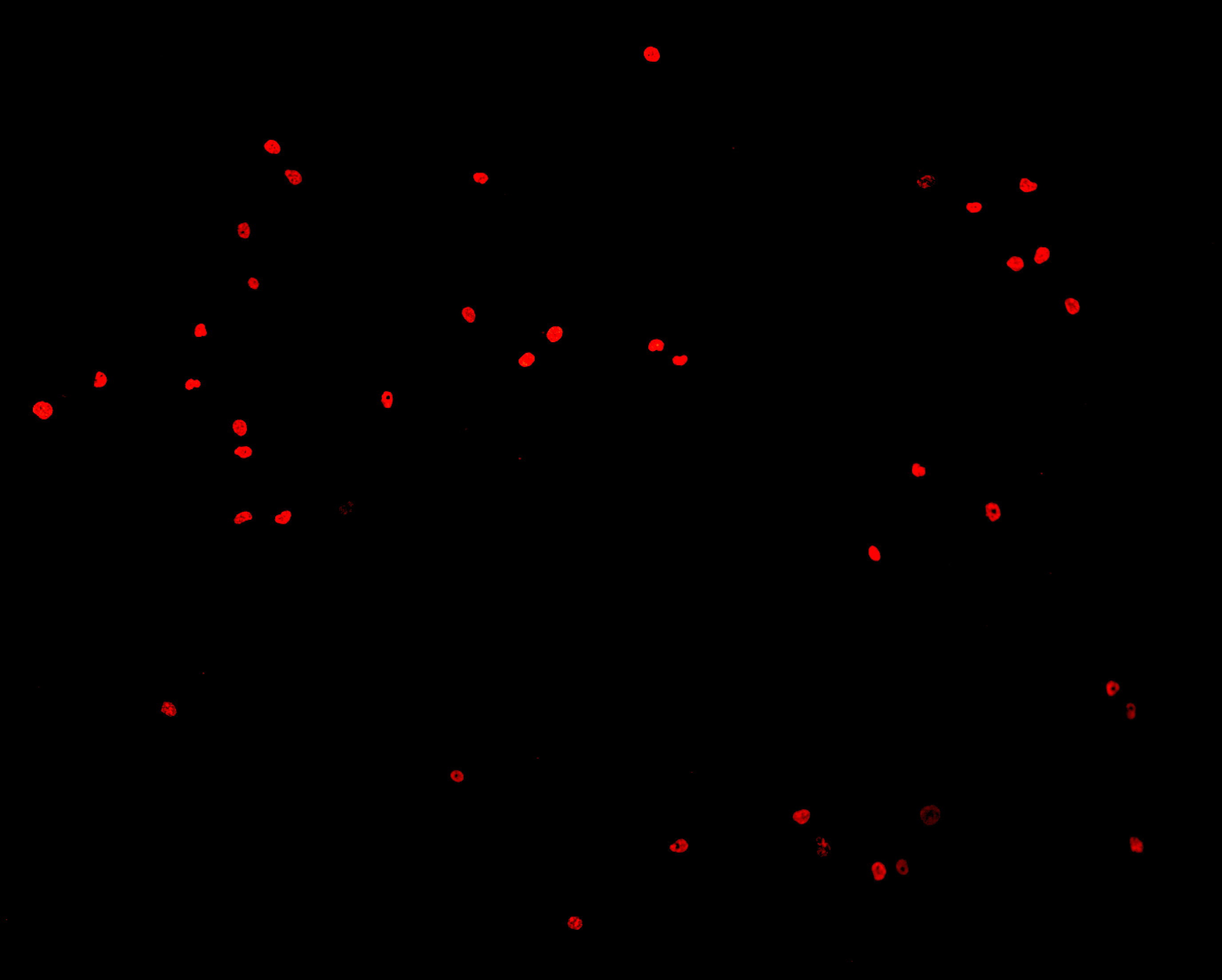

Supplement: Supplementary file 8 [file DataSheet4.ZIP › 拍摄-1190-图像导出-18.tif]

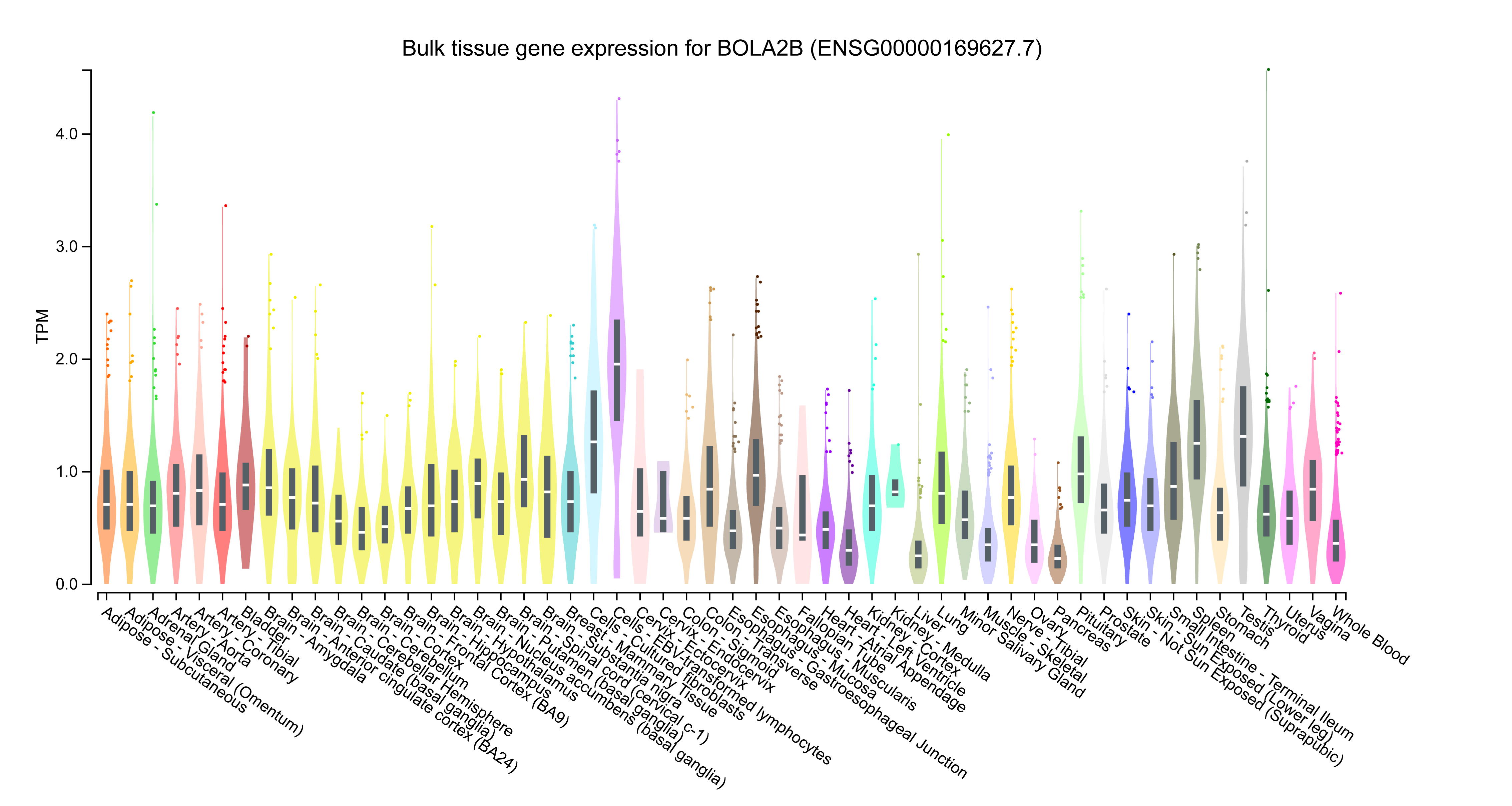

Supplement: Supplementary file 9 [file Image4.JPEG]

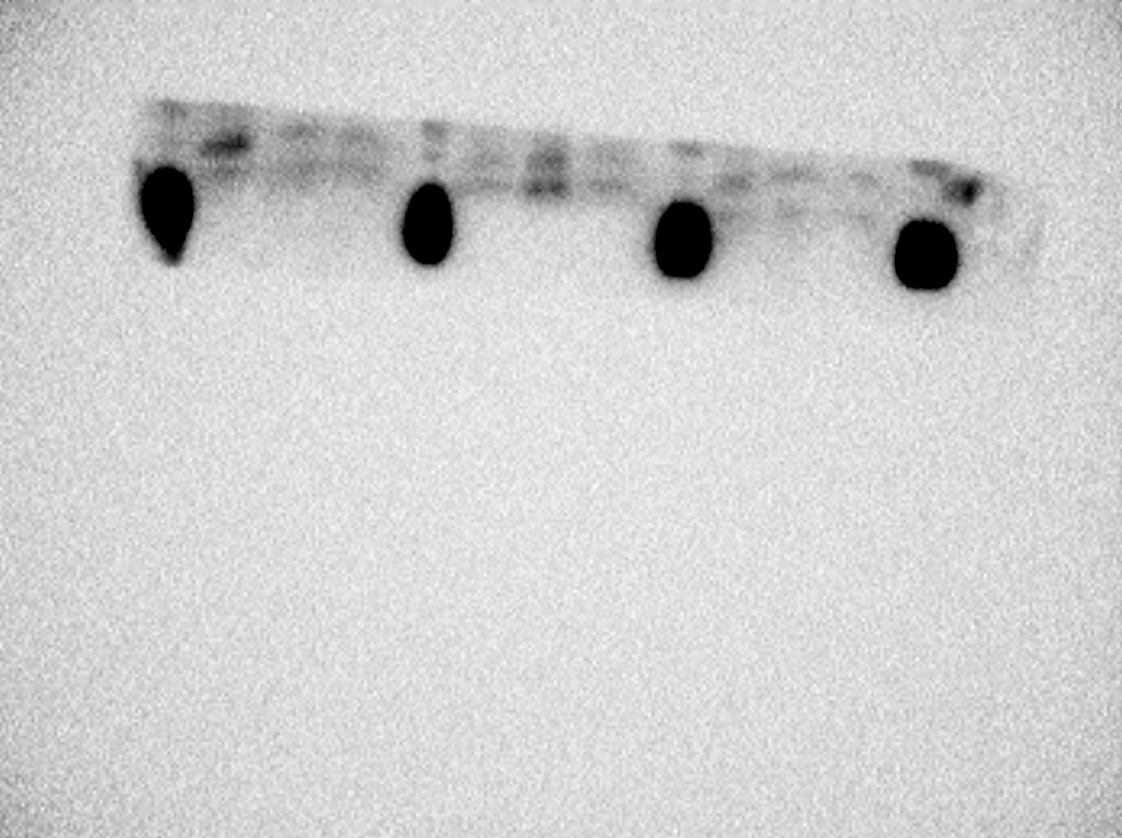

Supplement: Supplementary file 10 [file DataSheet1.ZIP › 231 BOLA2B.tif]

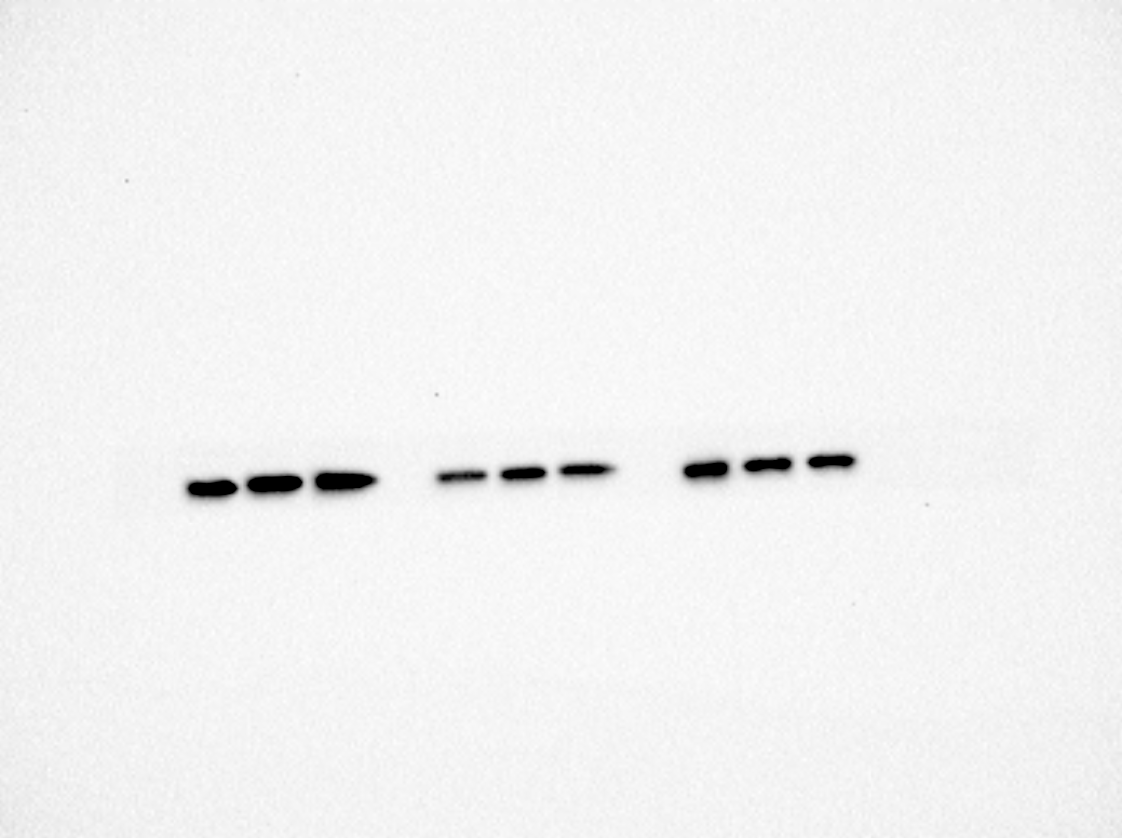

Supplement: Supplementary file 10 [file DataSheet1.ZIP › 231 TUBA.tif]

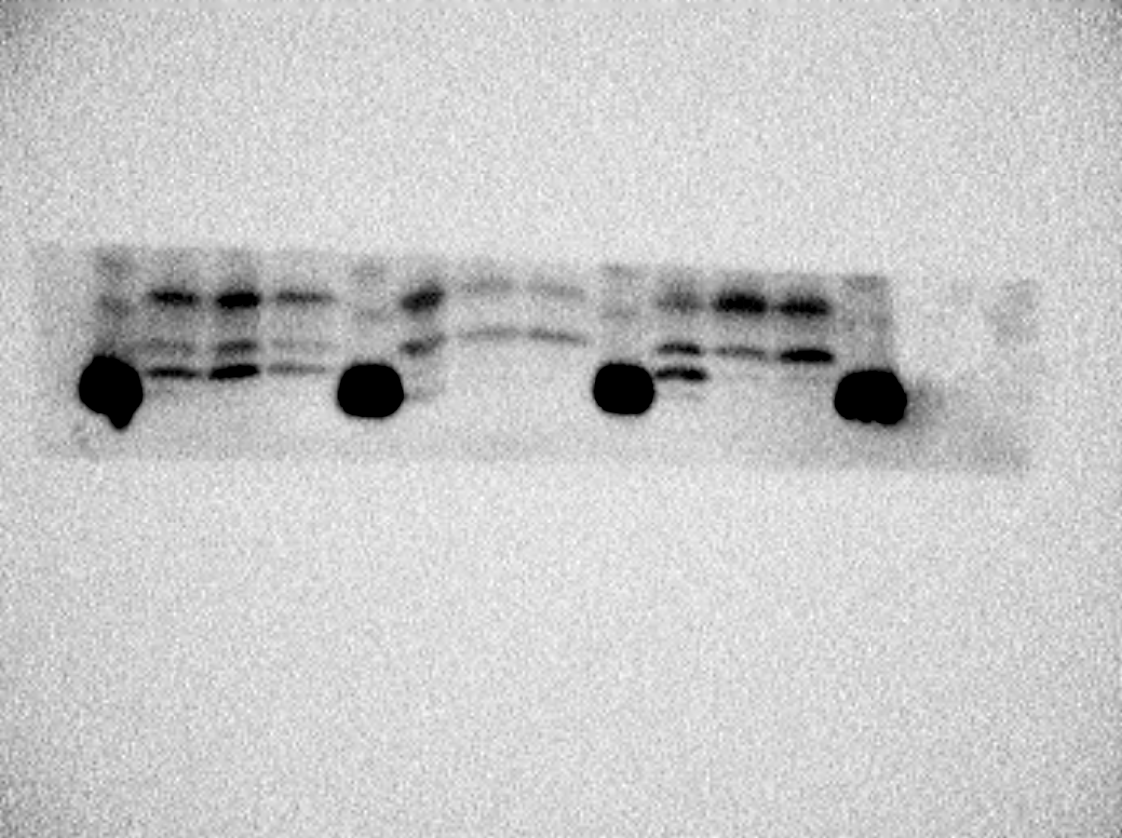

Supplement: Supplementary file 10 [file DataSheet1.ZIP › SKBR3 BOLA2B.tif]

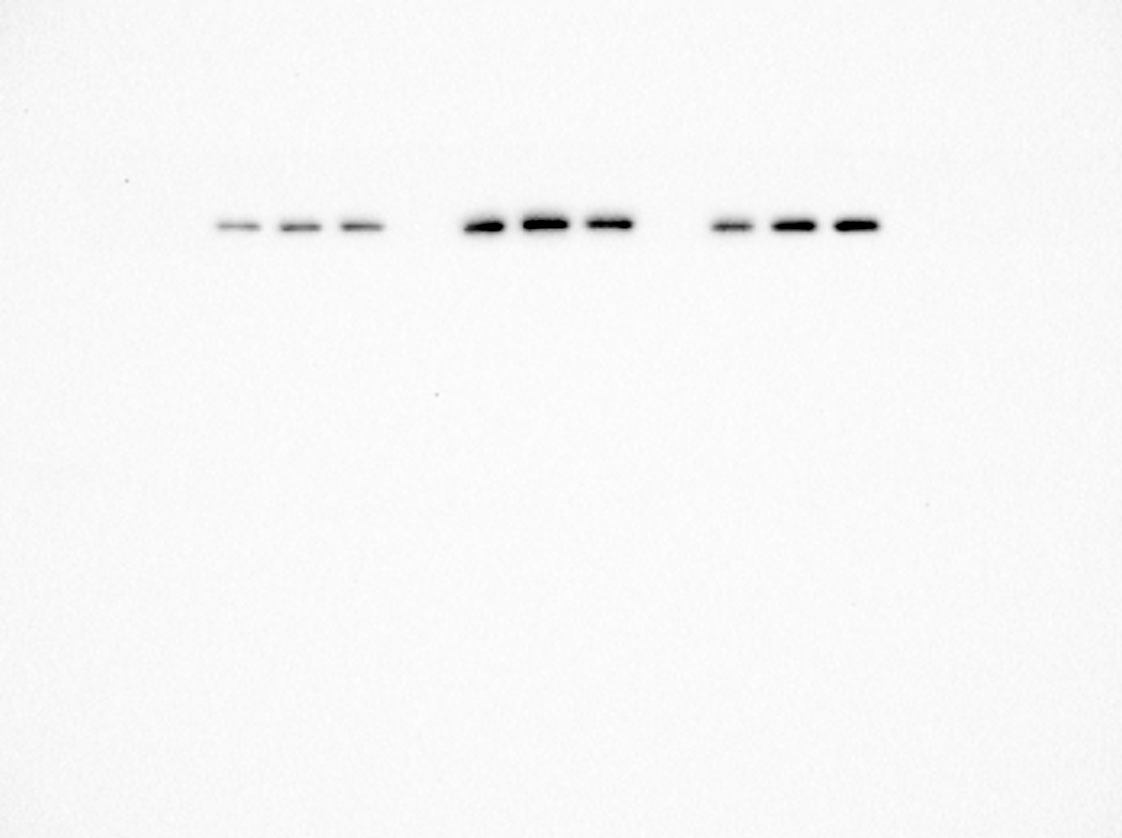

Supplement: Supplementary file 10 [file DataSheet1.ZIP › SKBR3 TUBA.tif]

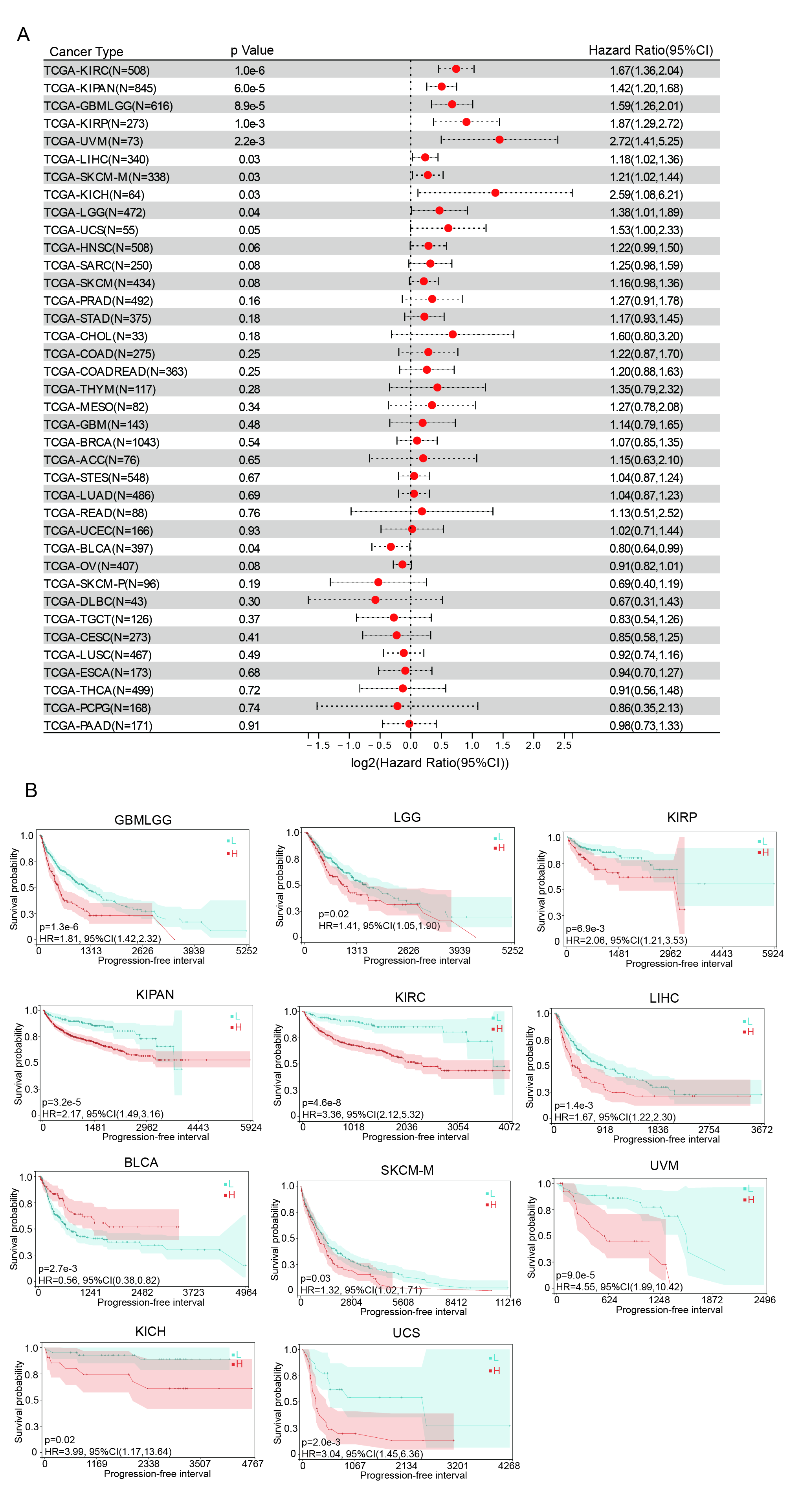

Supplement: Supplementary file 11 [file Image2.TIF]

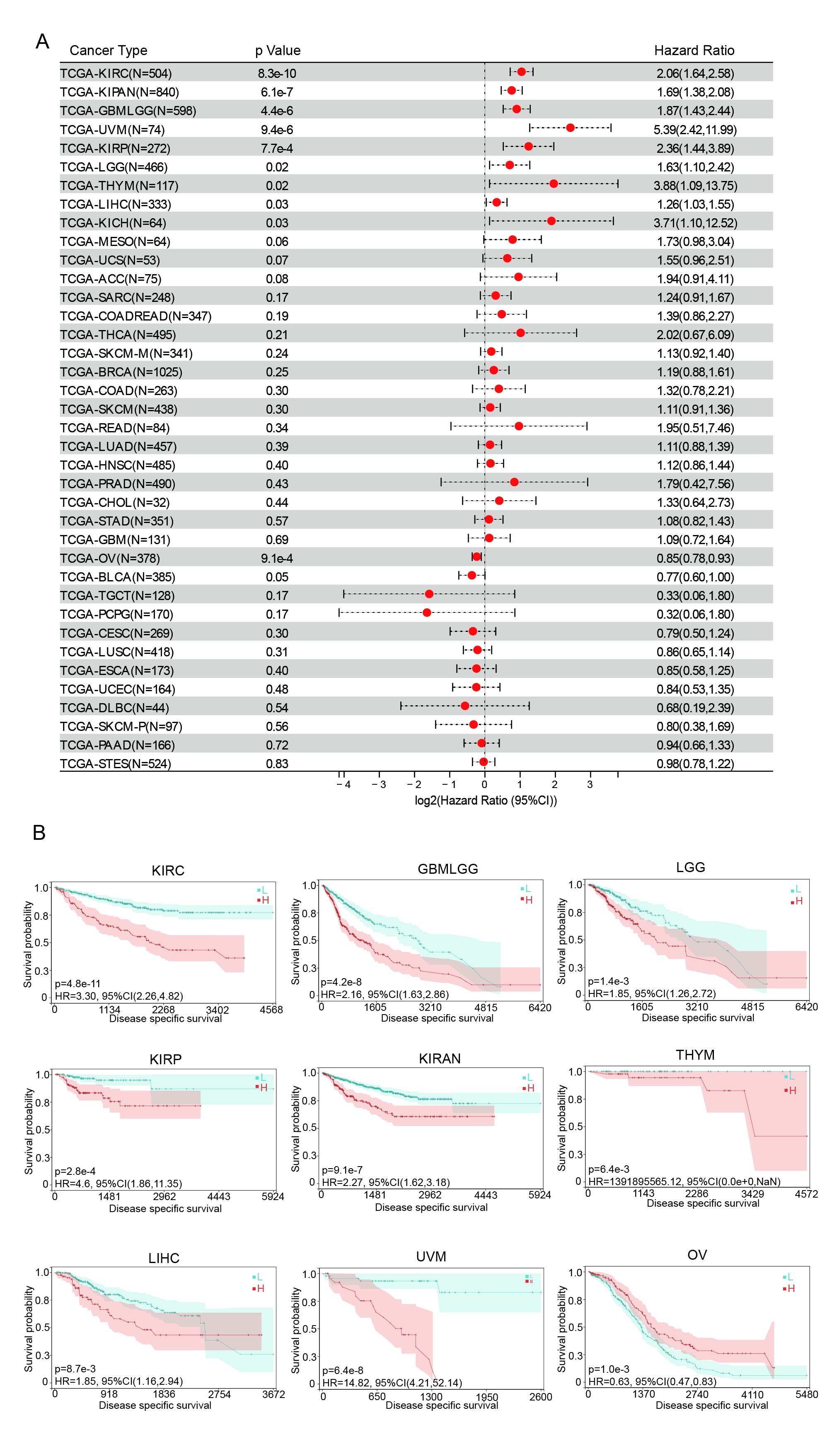

Supplement: Supplementary file 13 [file Image1.TIF]

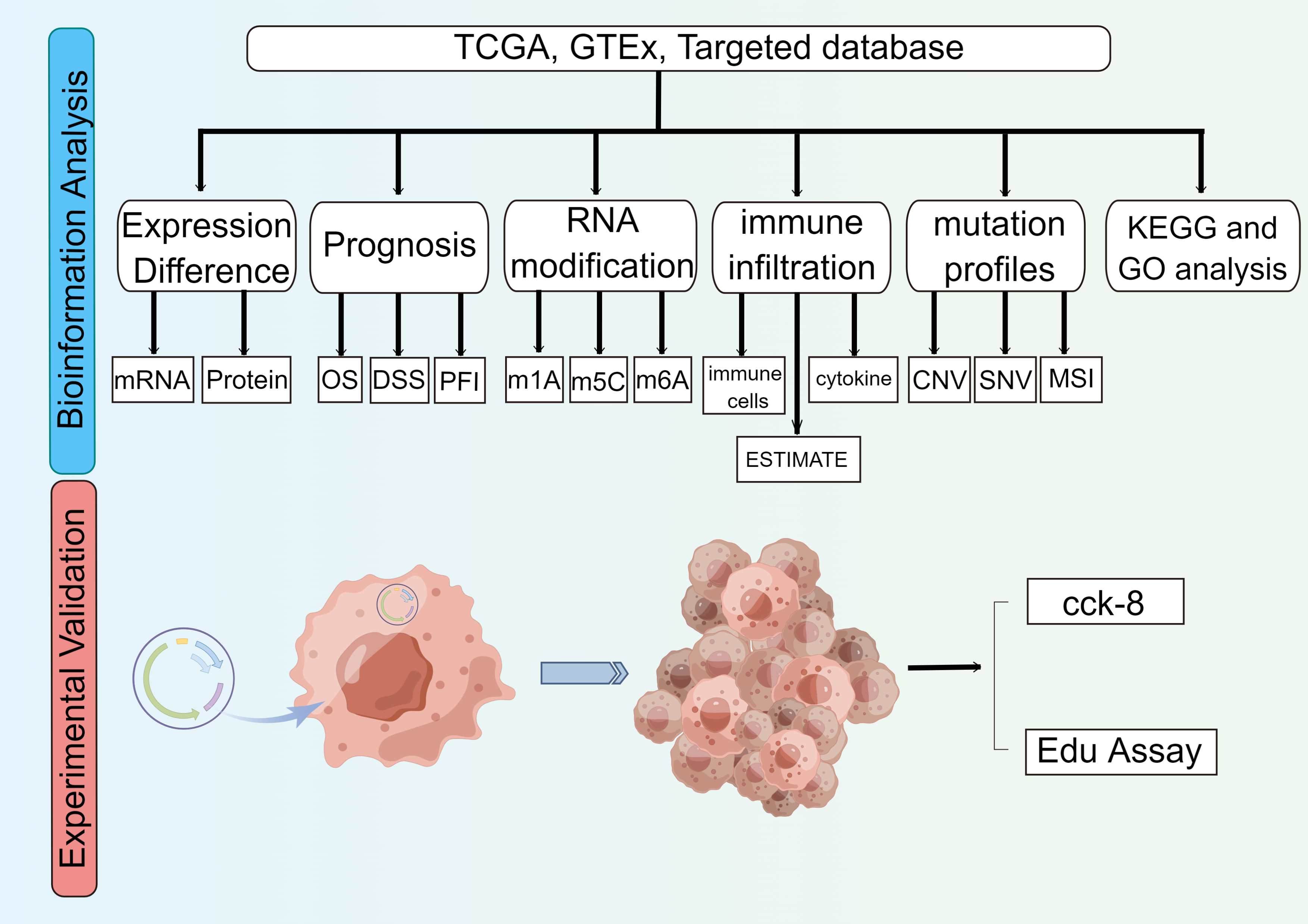

Supplement: Supplementary file 14 [file Image7.TIF]

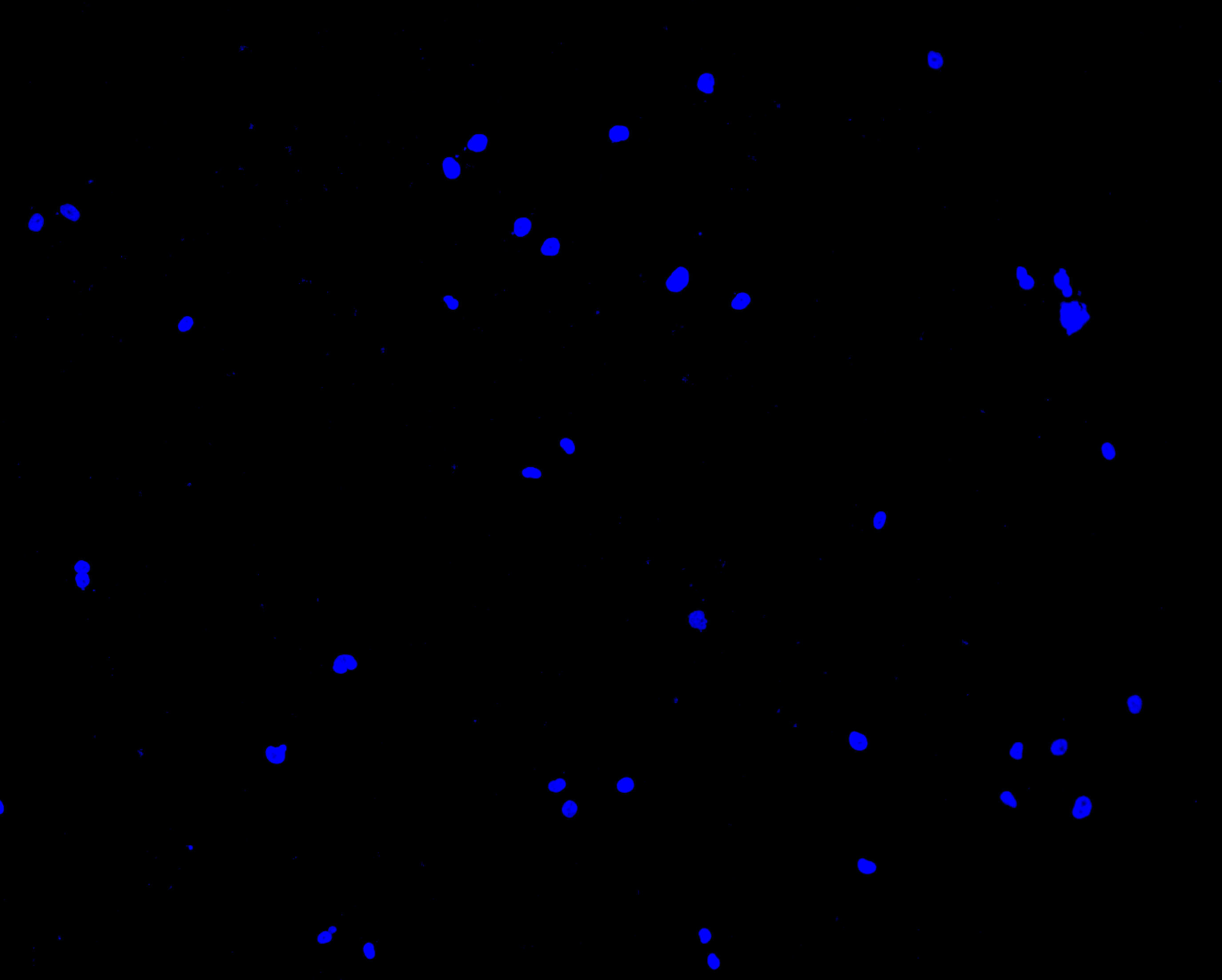

Supplement: Supplementary file 15 [file DataSheet6.ZIP › 拍摄-1235-图像导出-01.tif]

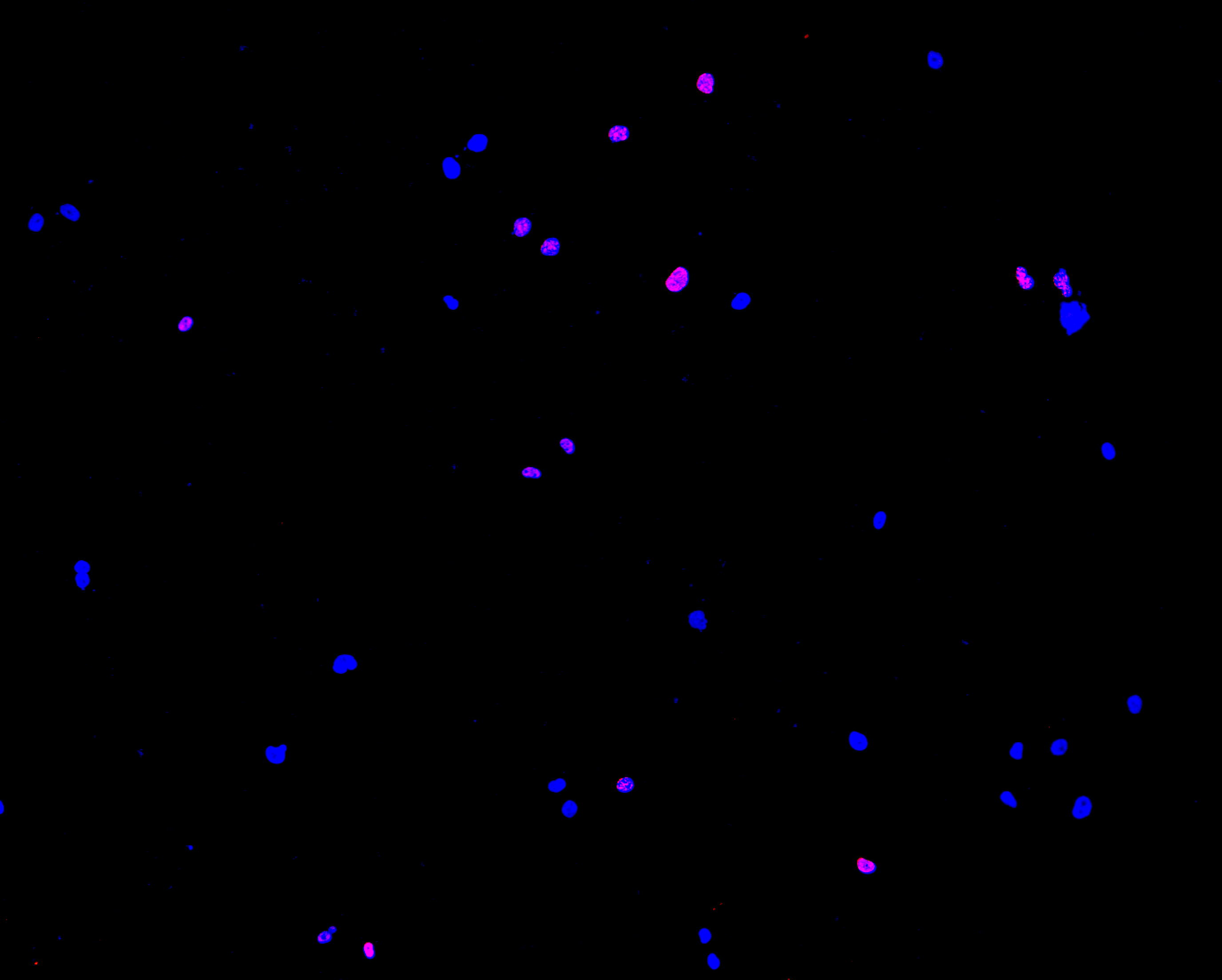

Supplement: Supplementary file 15 [file DataSheet6.ZIP › 拍摄-1235-添加通道-72-图像导出-02_c1+2.tif]

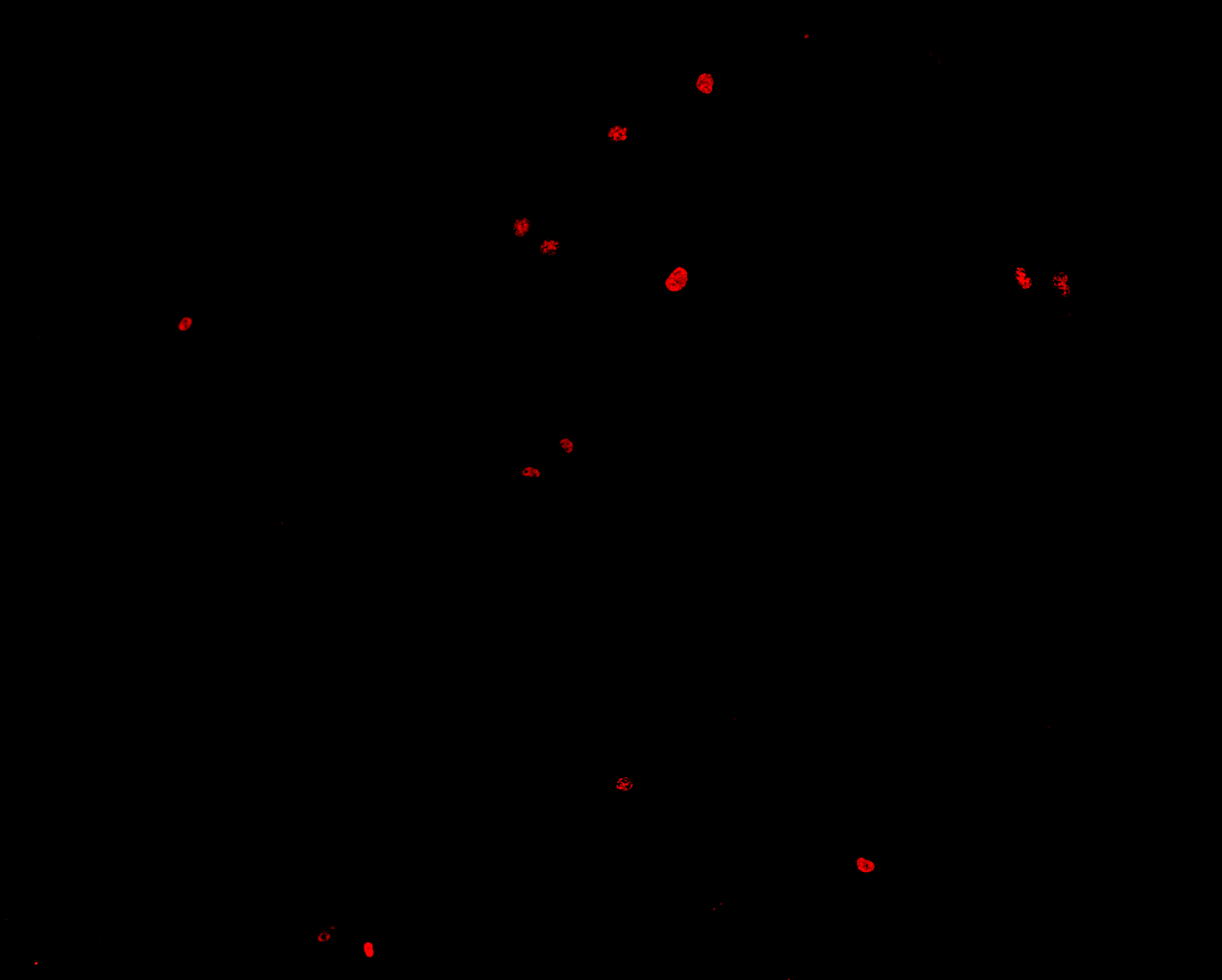

Supplement: Supplementary file 15 [file DataSheet6.ZIP › 拍摄-1236-图像导出-03.tif]

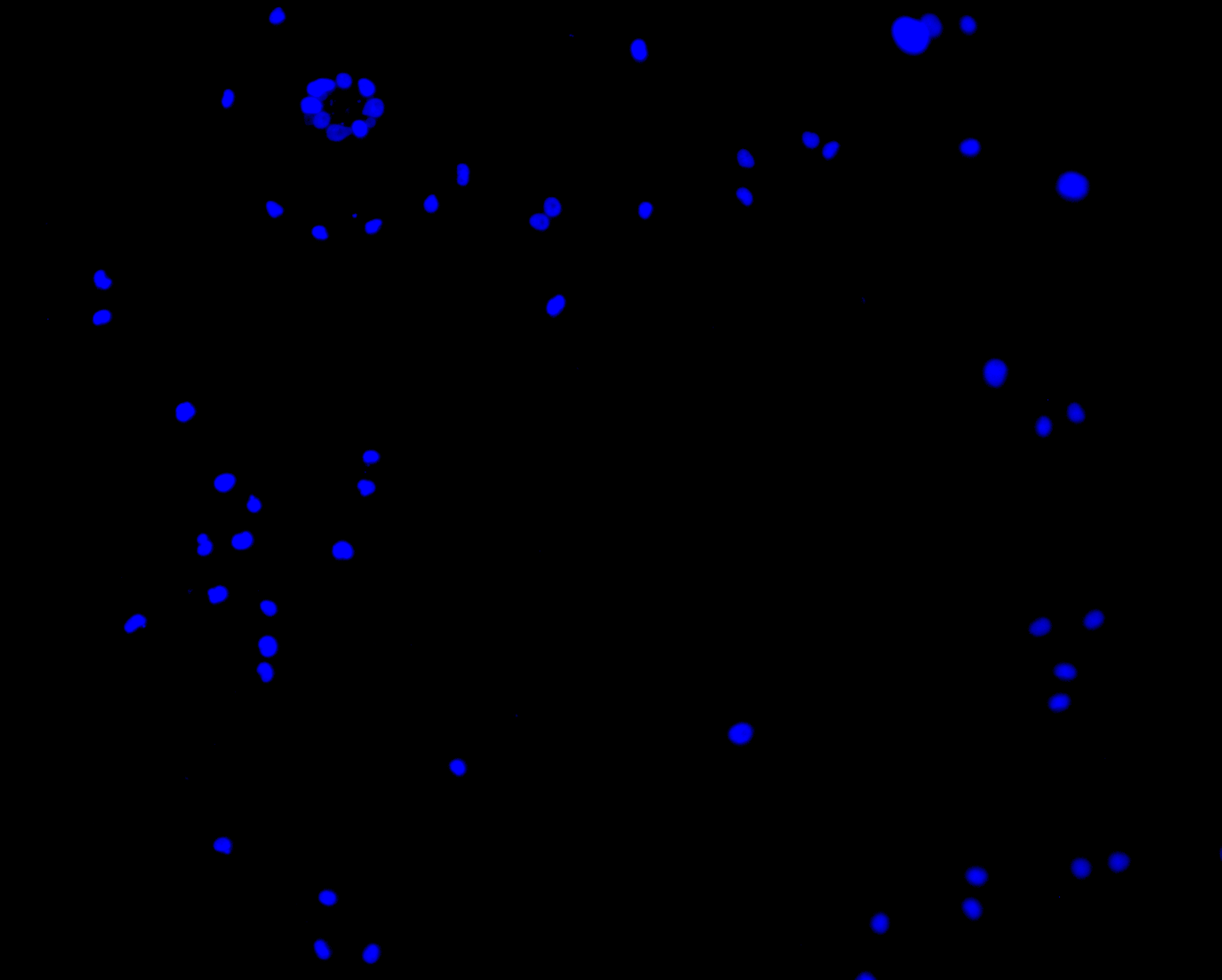

Supplement: Supplementary file 15 [file DataSheet6.ZIP › 拍摄-1239-图像导出-07.tif]

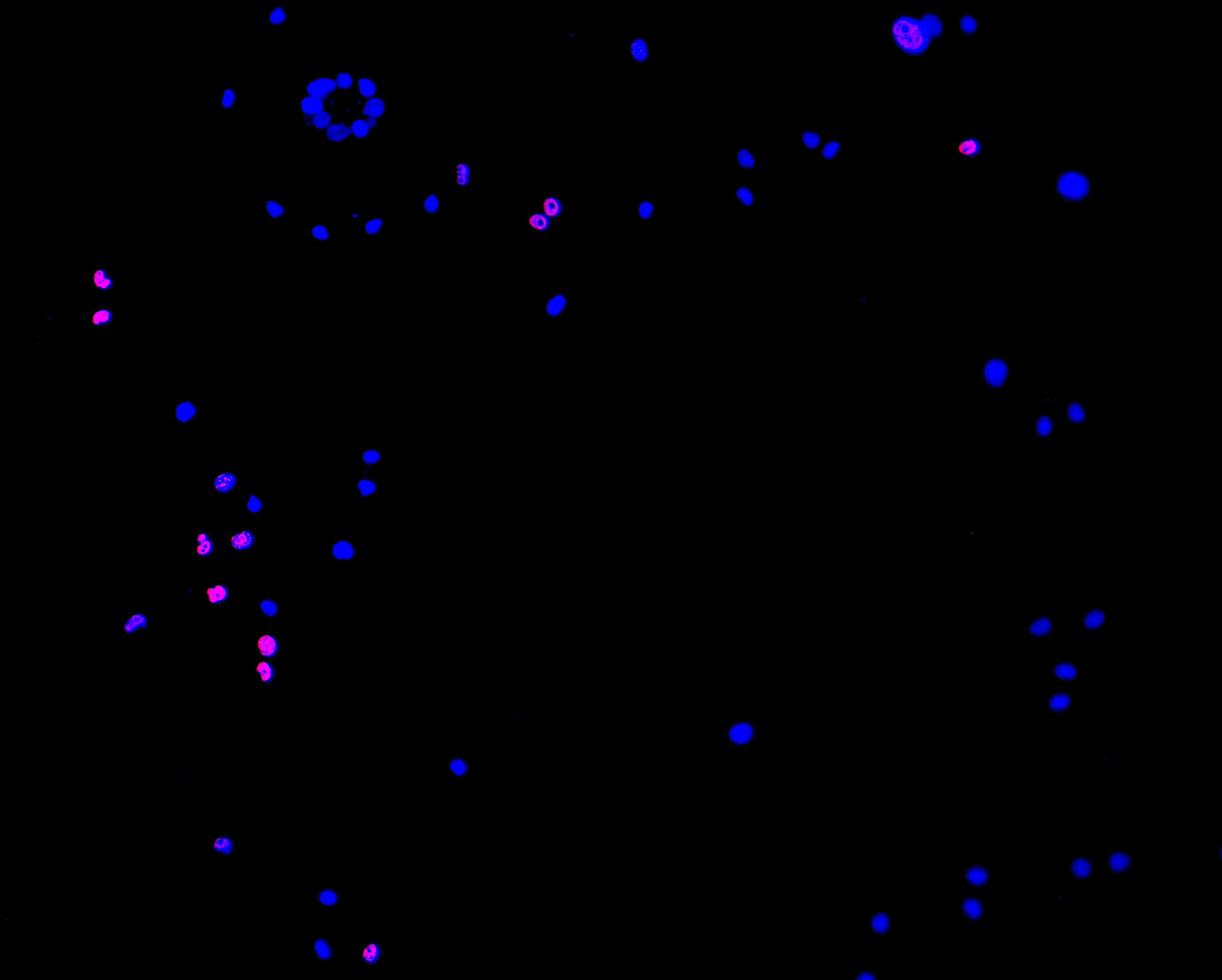

Supplement: Supplementary file 15 [file DataSheet6.ZIP › 拍摄-1239-添加通道-70-图像导出-08_c1+2.tif]

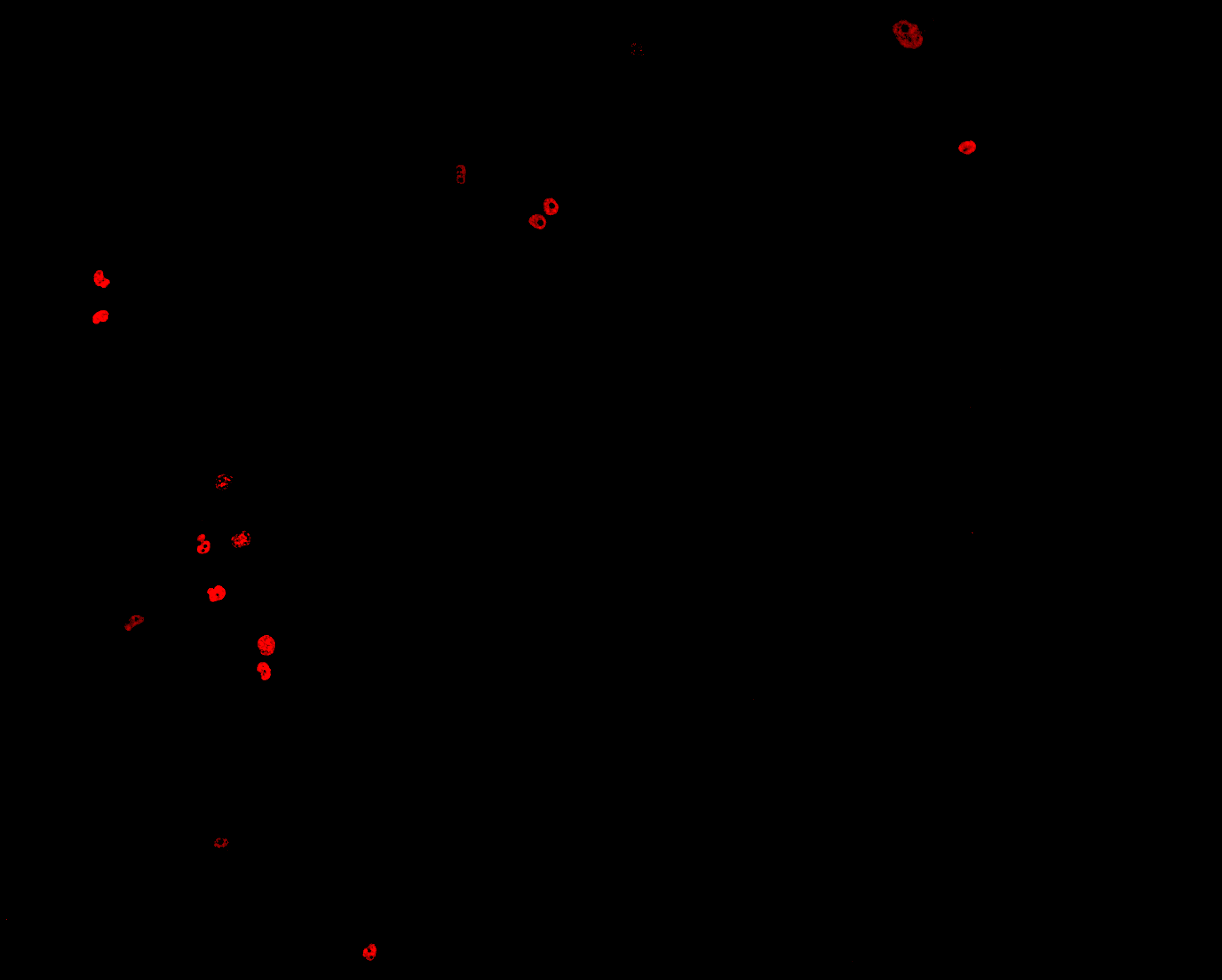

Supplement: Supplementary file 15 [file DataSheet6.ZIP › 拍摄-1240-图像导出-09.tif]

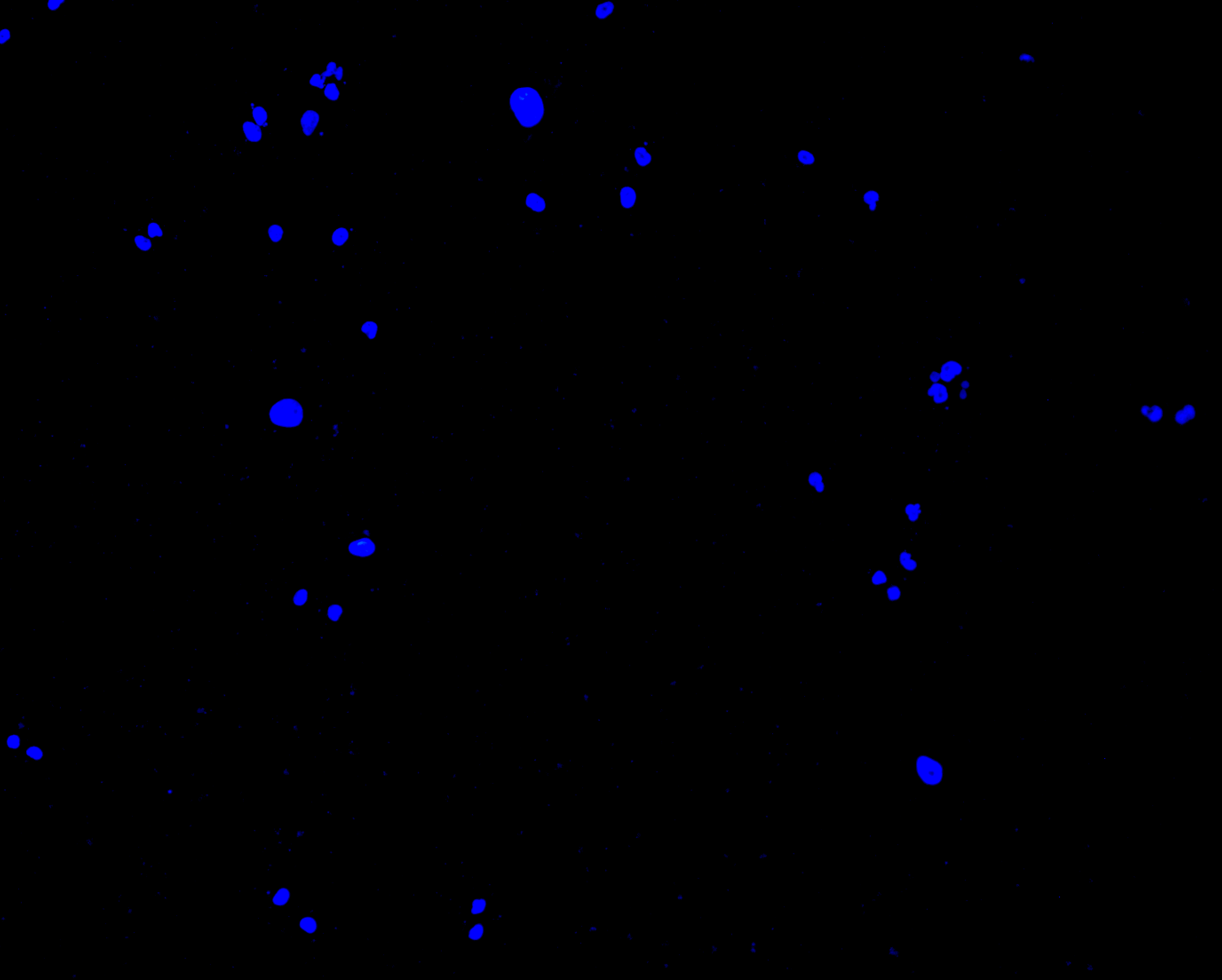

Supplement: Supplementary file 15 [file DataSheet6.ZIP › 拍摄-1243-图像导出-13.tif]

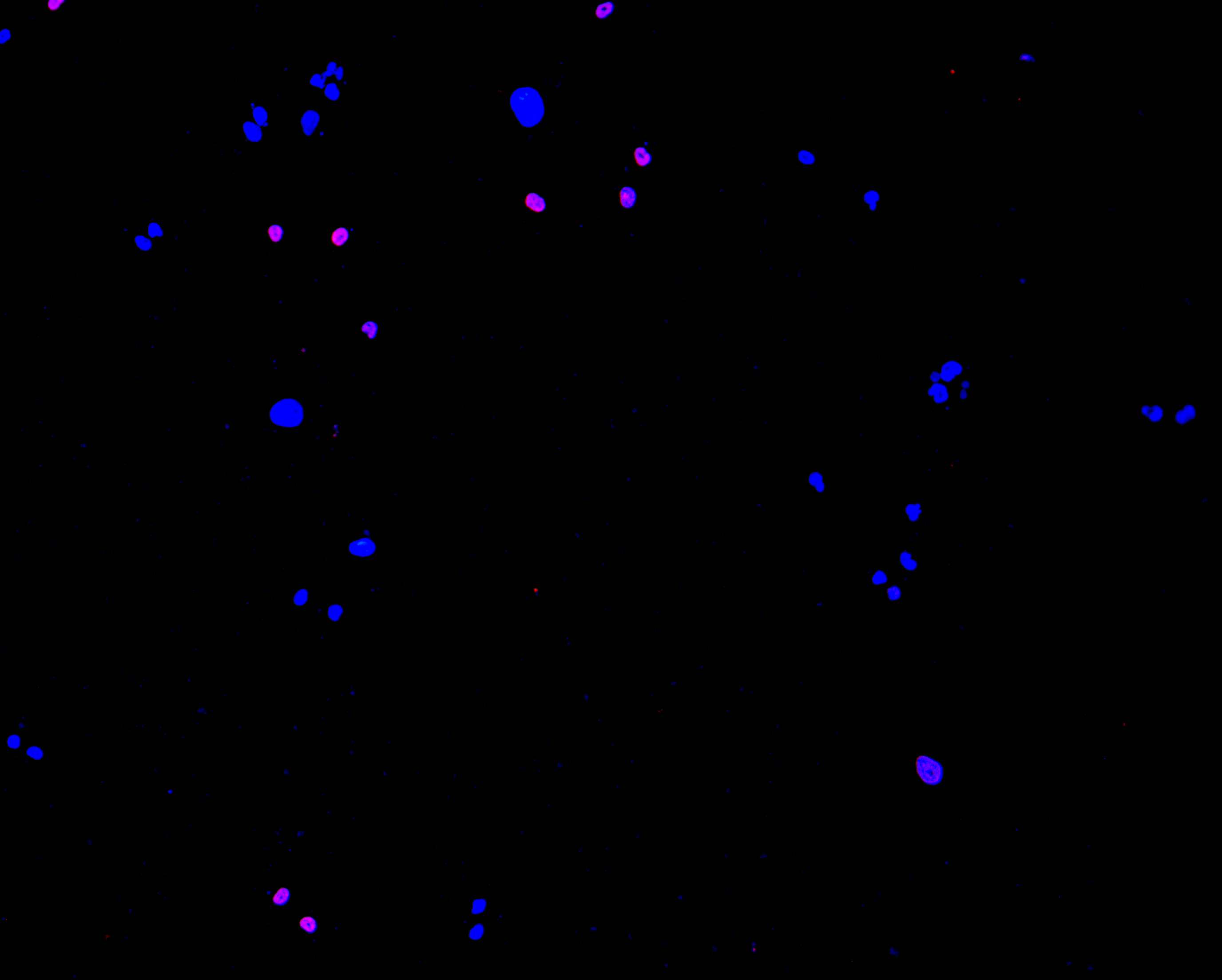

Supplement: Supplementary file 15 [file DataSheet6.ZIP › 拍摄-1243-添加通道-71-图像导出-14_c1+2.tif]

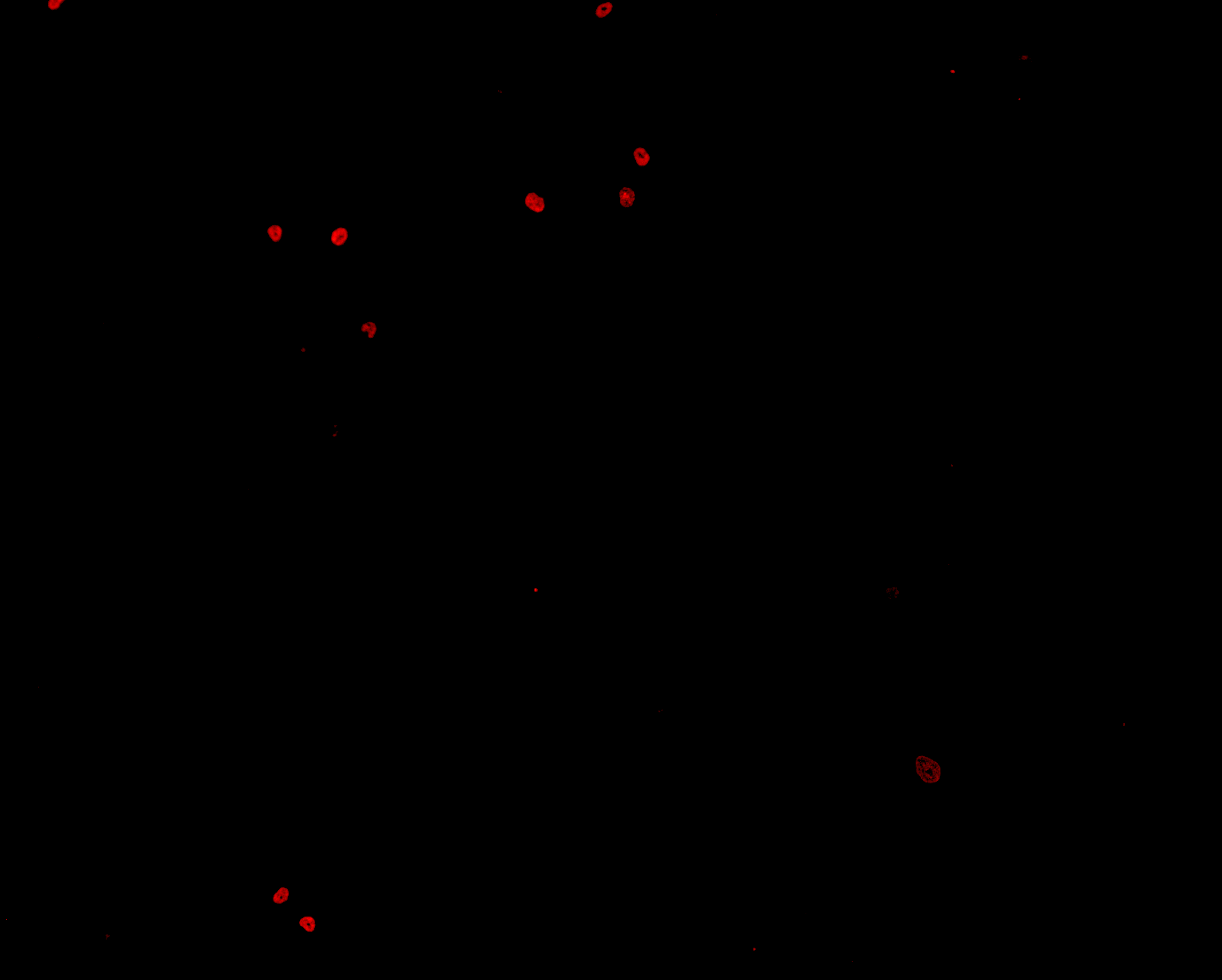

Supplement: Supplementary file 15 [file DataSheet6.ZIP › 拍摄-1244-图像导出-15.tif]

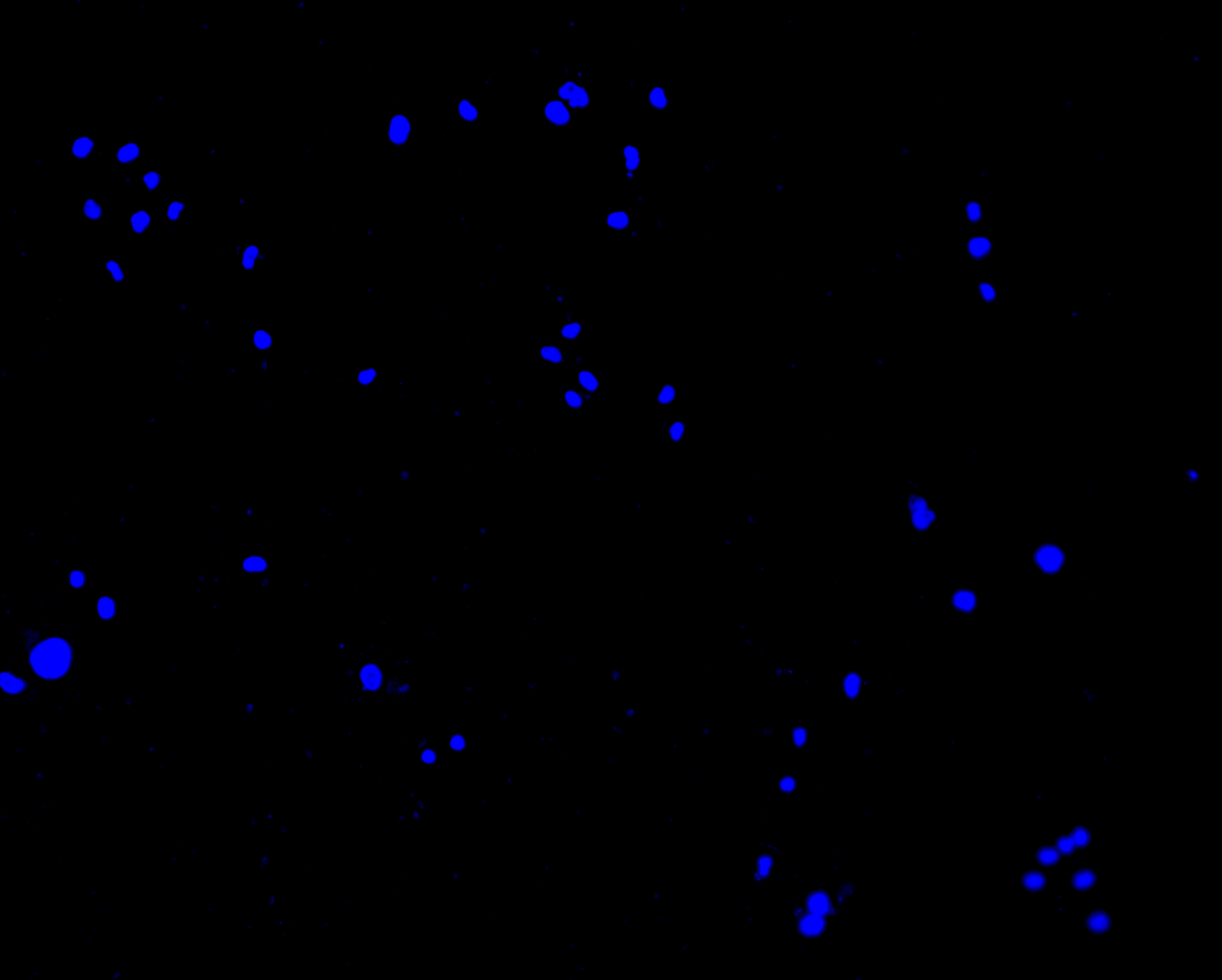

Supplement: Supplementary file 17 [file DataSheet5.ZIP › 拍摄-1217-图像导出-16.tif]

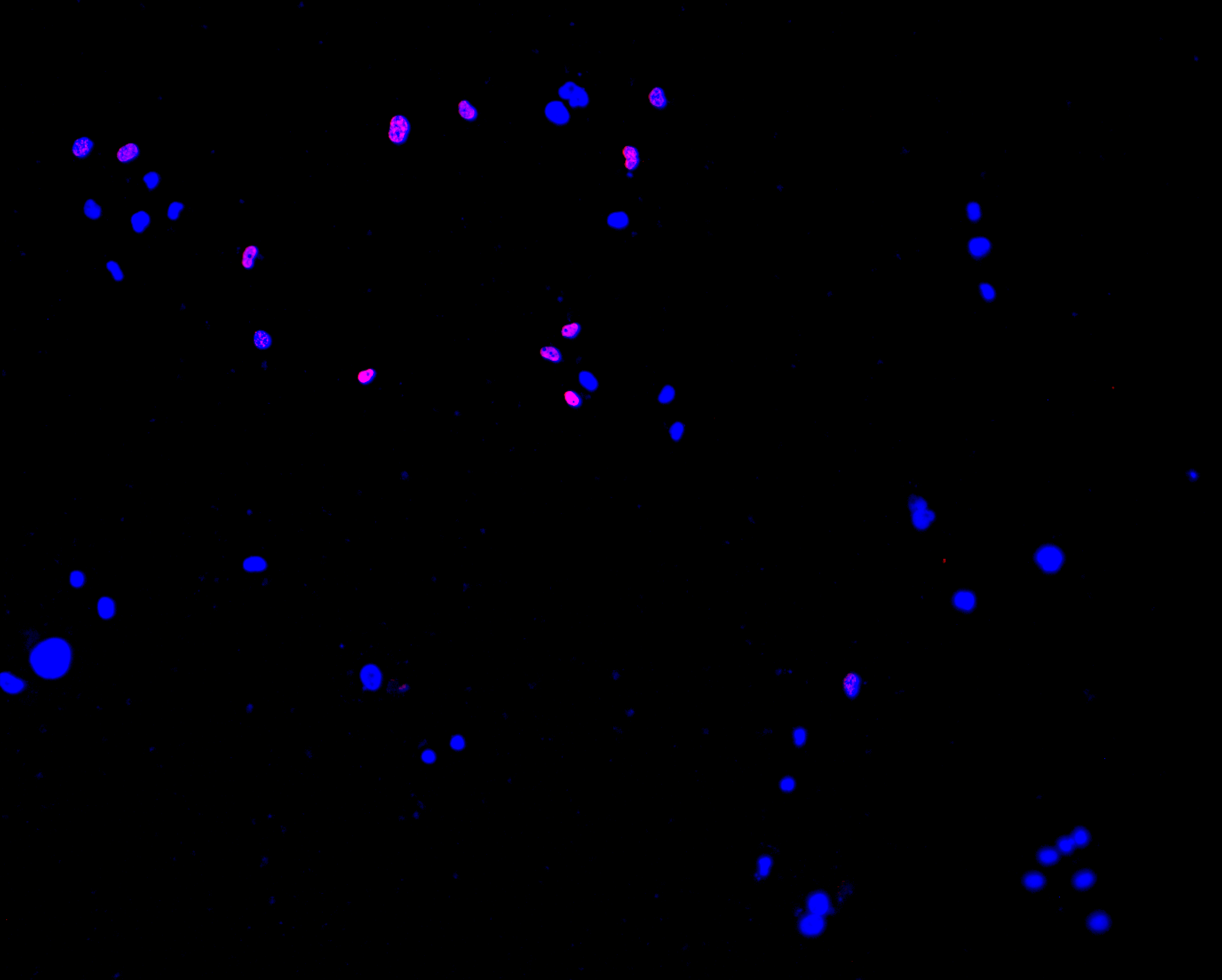

Supplement: Supplementary file 17 [file DataSheet5.ZIP › 拍摄-1217-添加通道-64-图像导出-17_c1+2.tif]

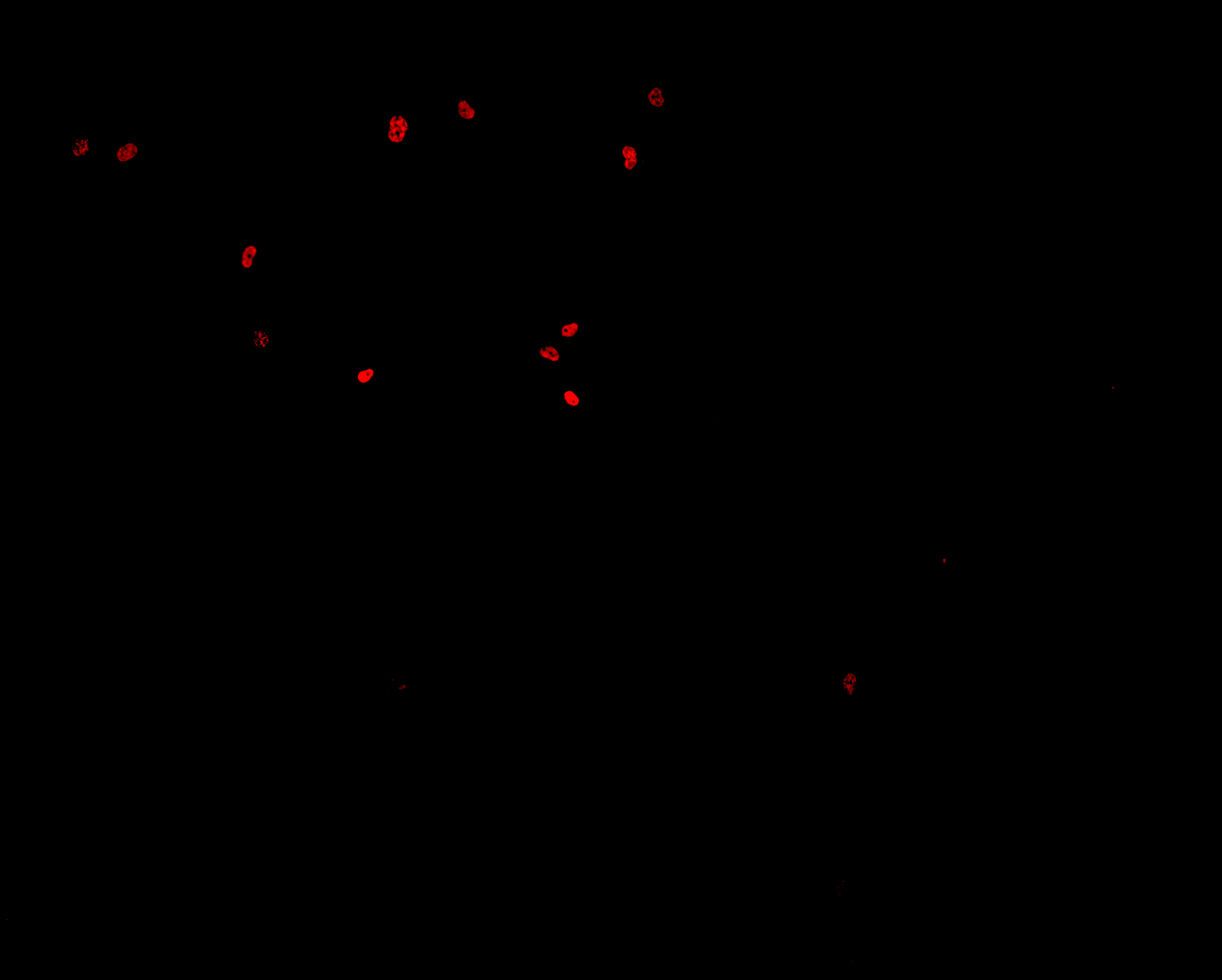

Supplement: Supplementary file 17 [file DataSheet5.ZIP › 拍摄-1218-图像导出-18.tif]

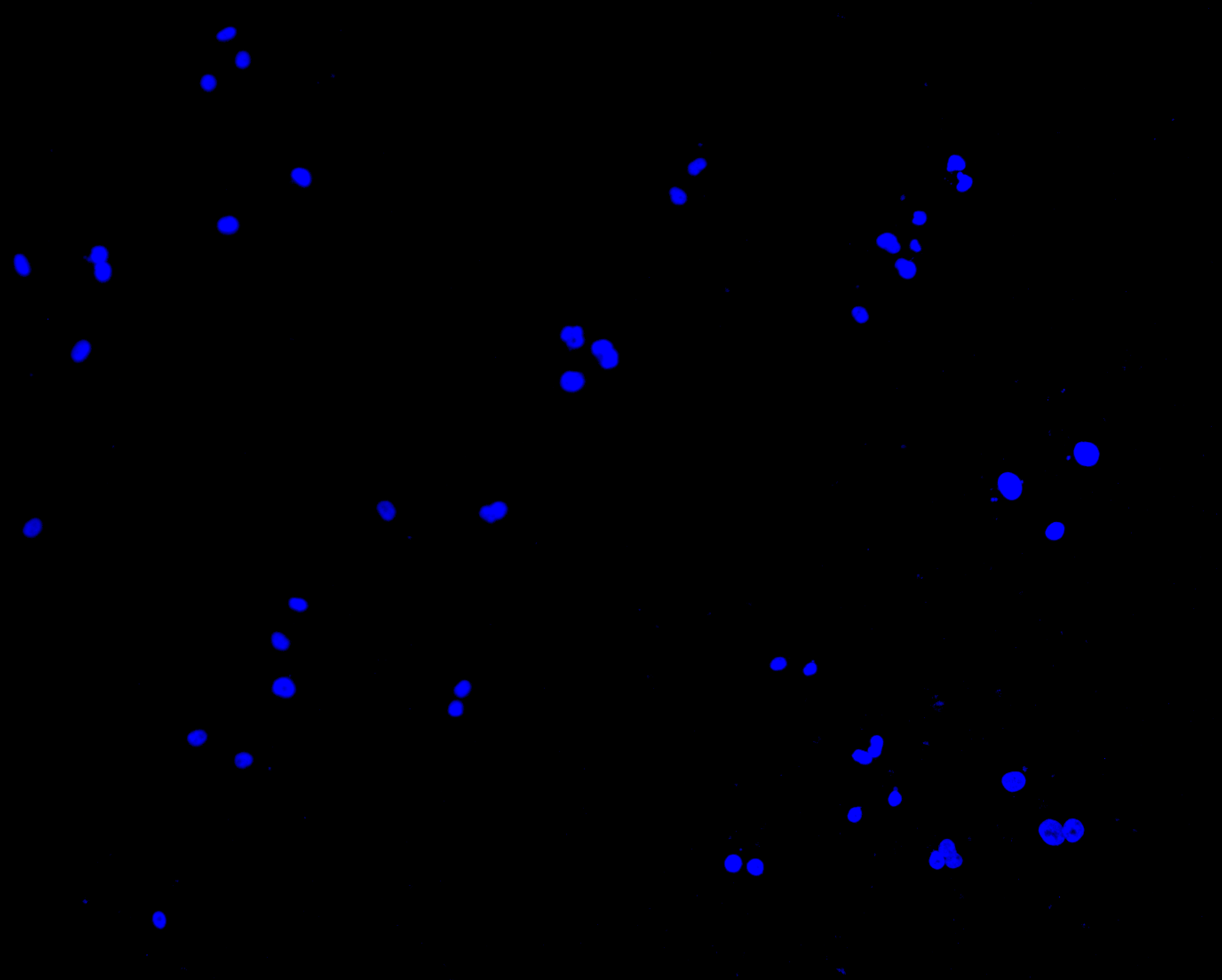

Supplement: Supplementary file 17 [file DataSheet5.ZIP › 拍摄-1229-图像导出-34.tif]

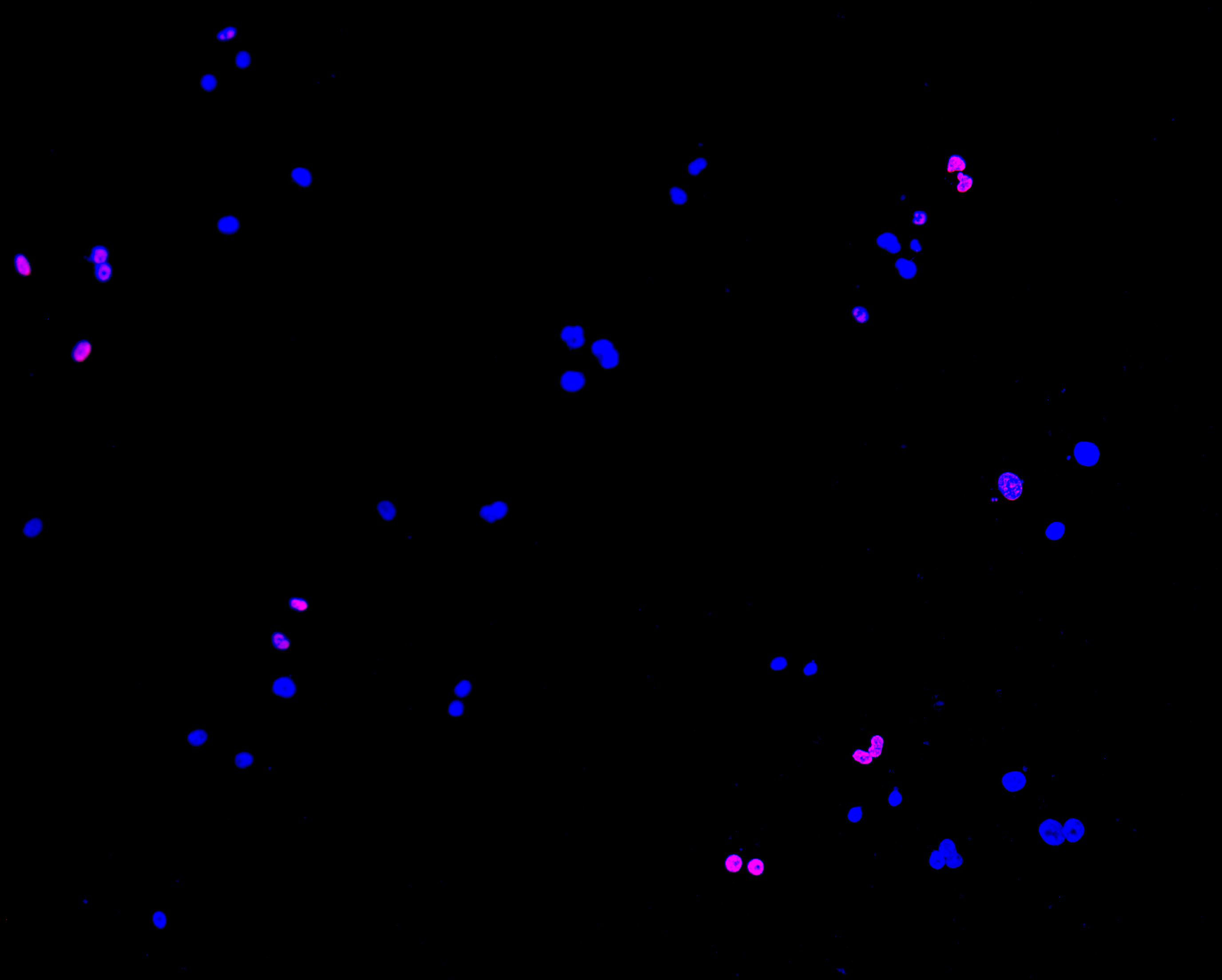

Supplement: Supplementary file 17 [file DataSheet5.ZIP › 拍摄-1229-添加通道-56-图像导出-35_c1+2.tif]

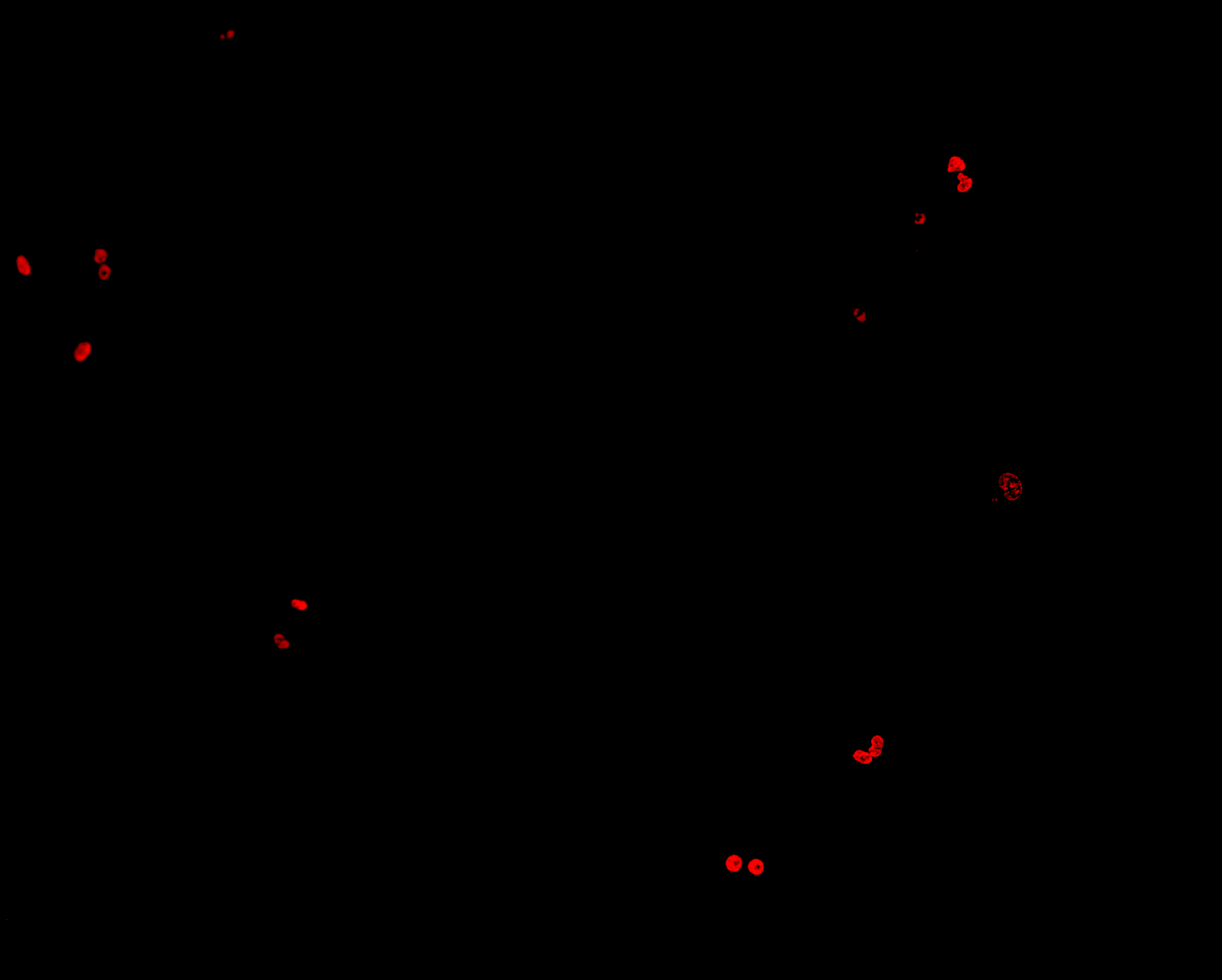

Supplement: Supplementary file 17 [file DataSheet5.ZIP › 拍摄-1230-图像导出-36.tif]

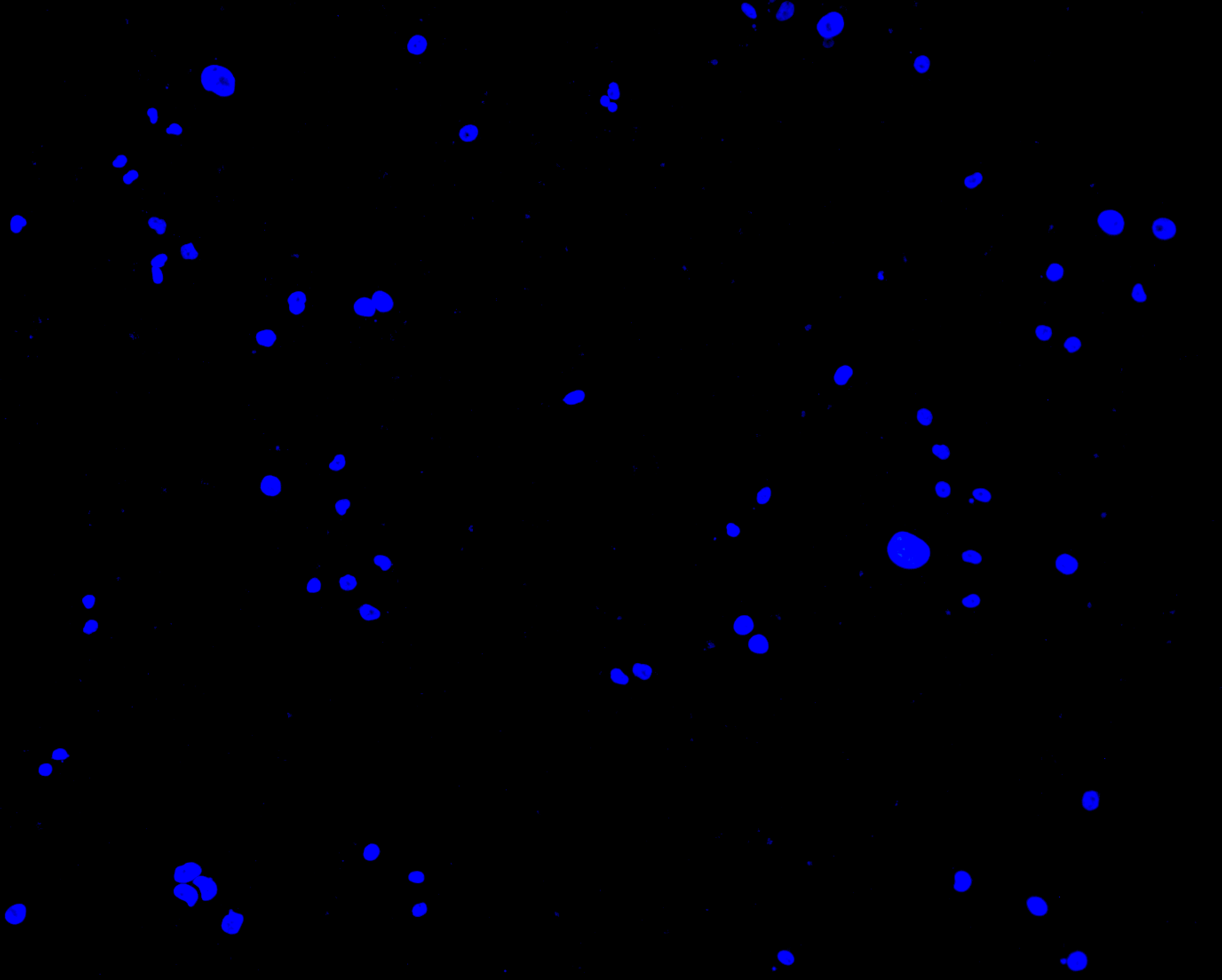

Supplement: Supplementary file 17 [file DataSheet5.ZIP › 拍摄-1231-图像导出-37.tif]

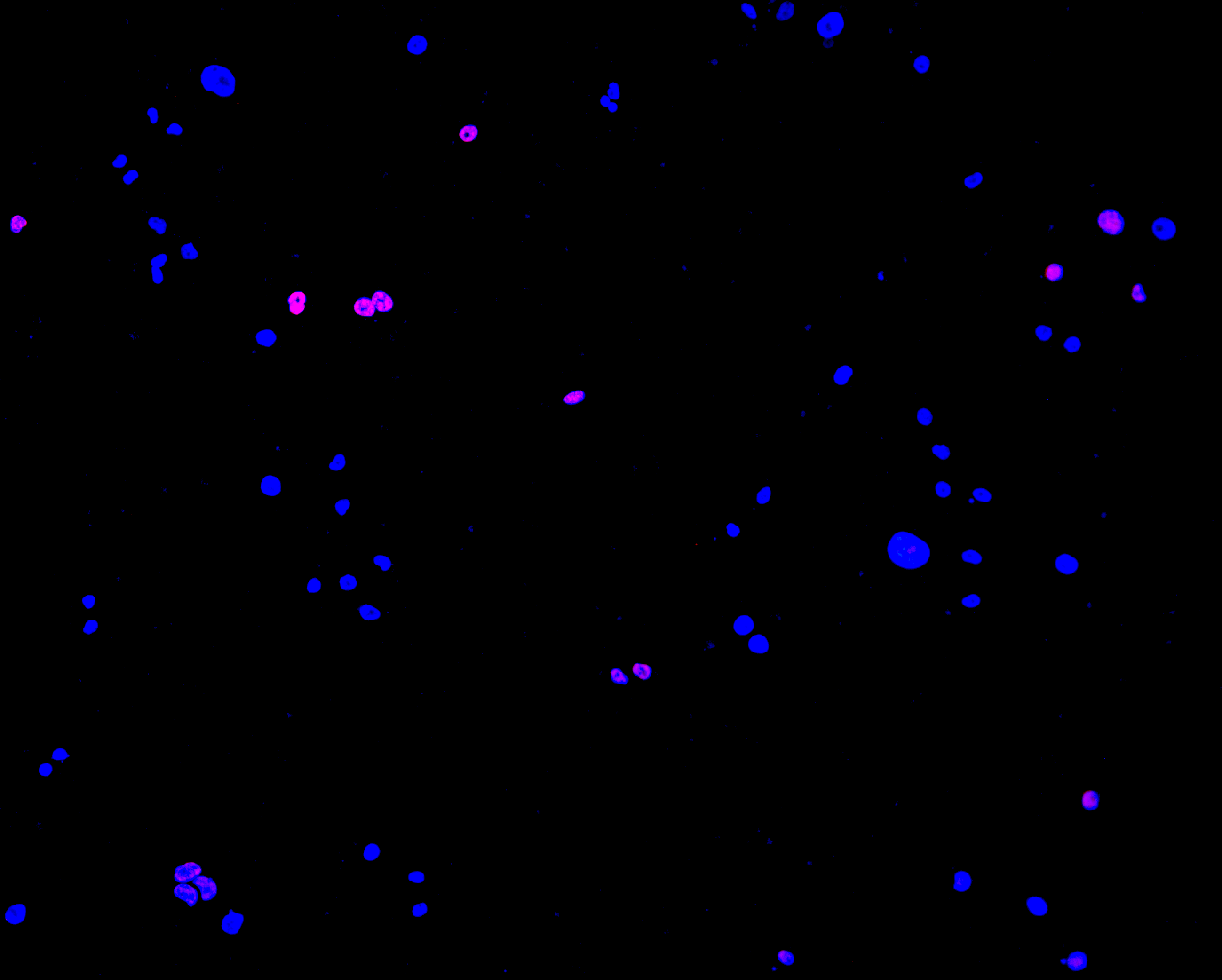

Supplement: Supplementary file 17 [file DataSheet5.ZIP › 拍摄-1231-添加通道-57-图像导出-38_c1+2.tif]

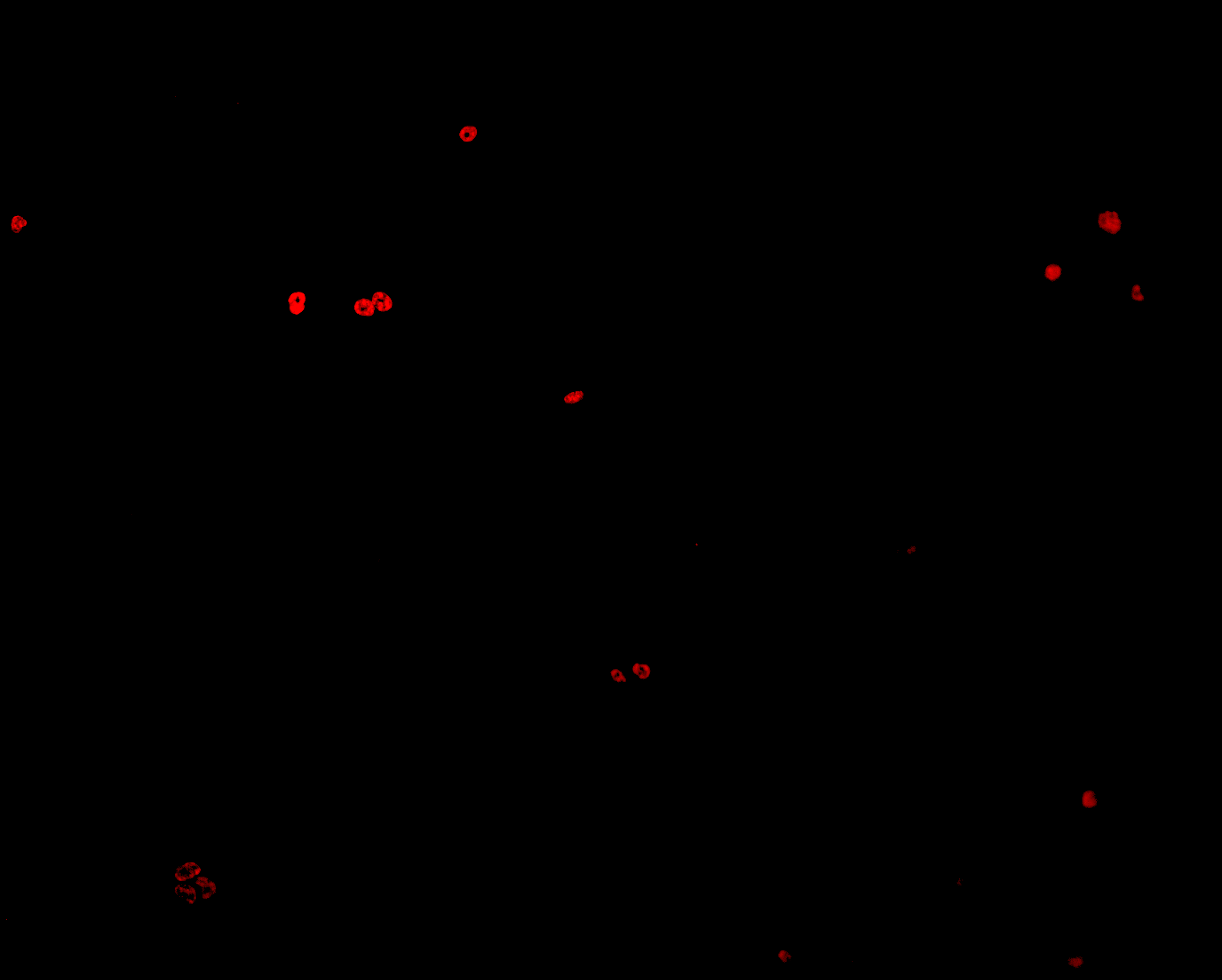

Supplement: Supplementary file 17 [file DataSheet5.ZIP › 拍摄-1232-图像导出-39.tif]

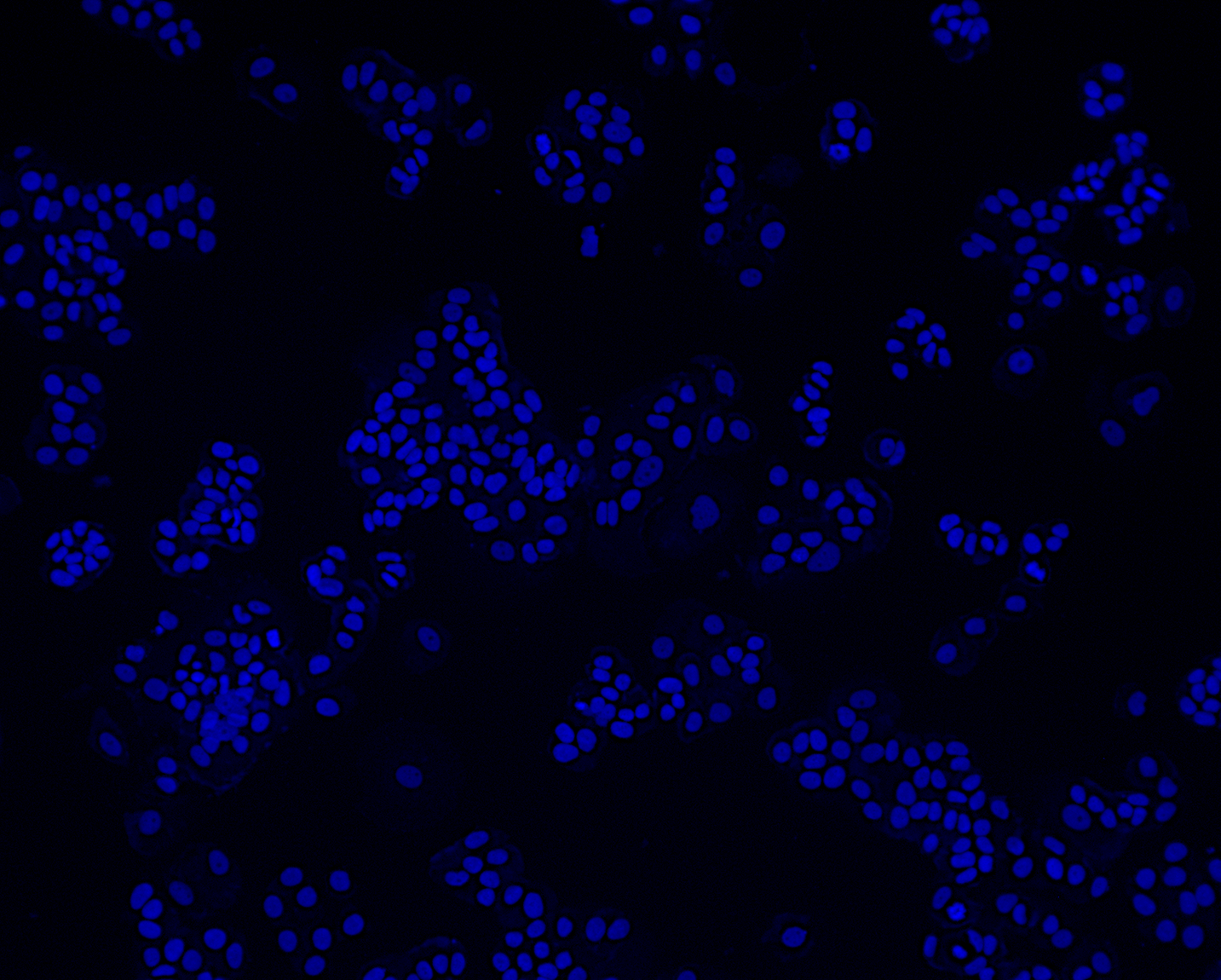

Supplement: Supplementary file 19 [file DataSheet7.ZIP › 拍摄-1282-图像导出-46.tif]

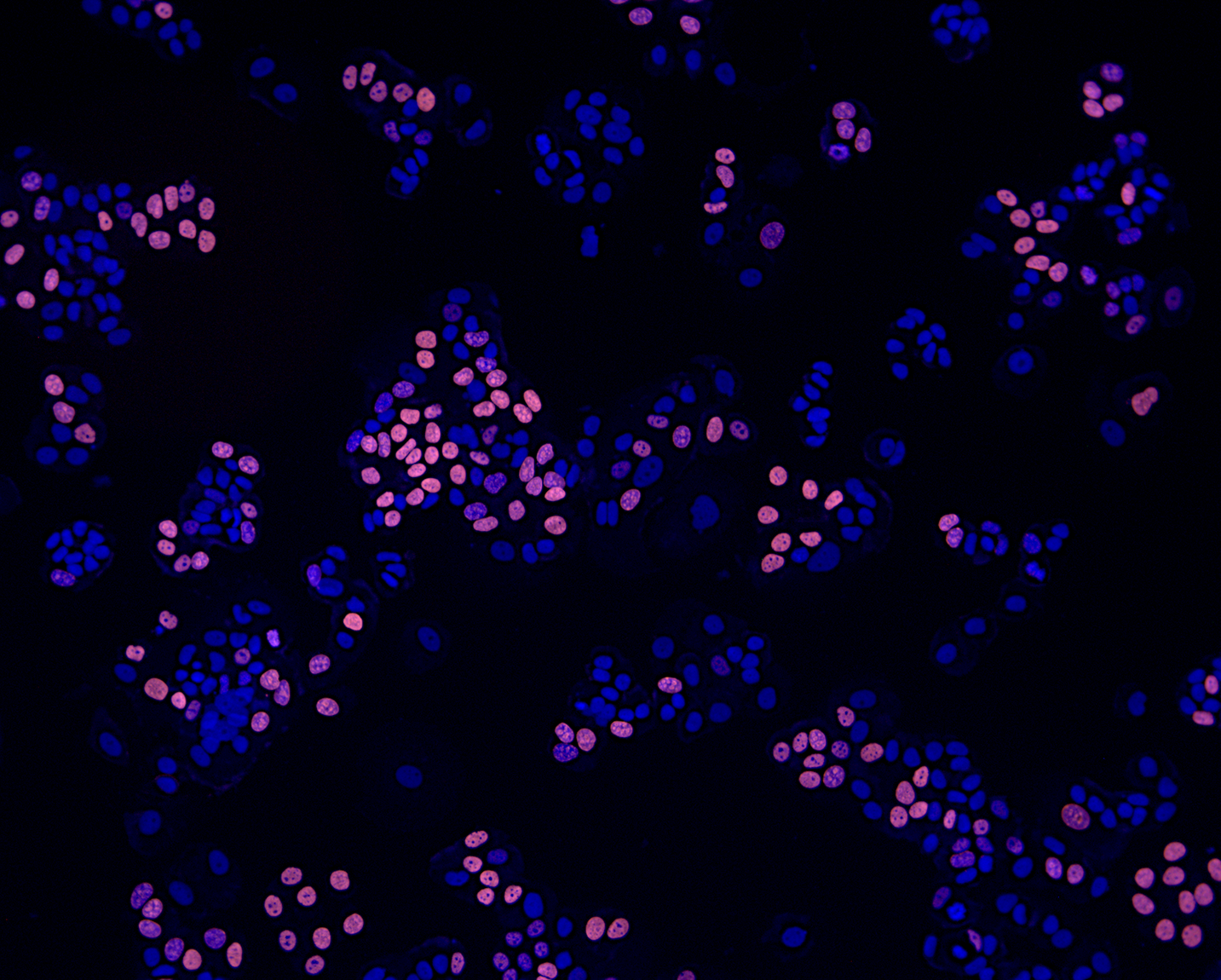

Supplement: Supplementary file 19 [file DataSheet7.ZIP › 拍摄-1282-添加通道-18-图像导出-47_c1+2.tif]

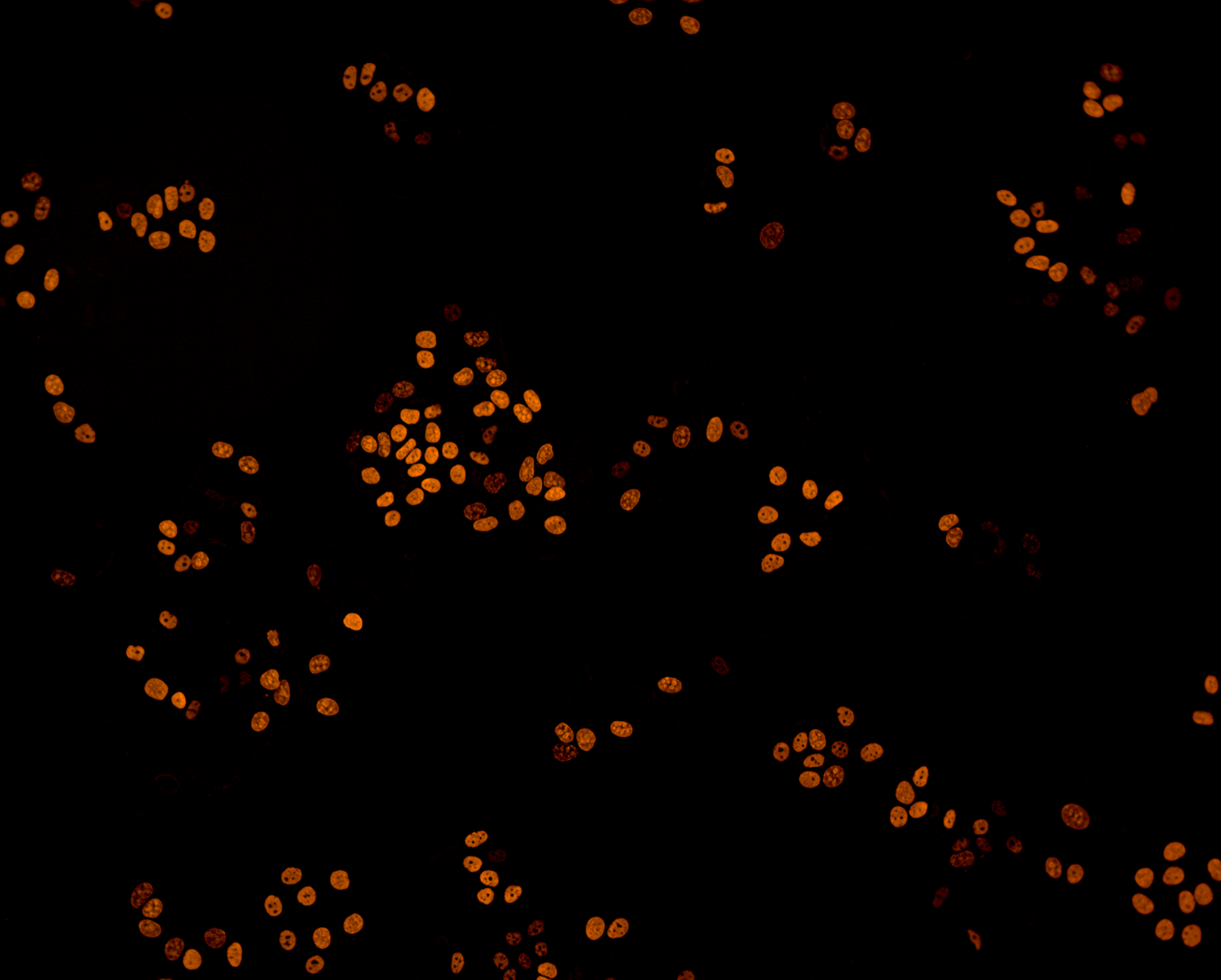

Supplement: Supplementary file 19 [file DataSheet7.ZIP › 拍摄-1283-图像导出-48.tif]

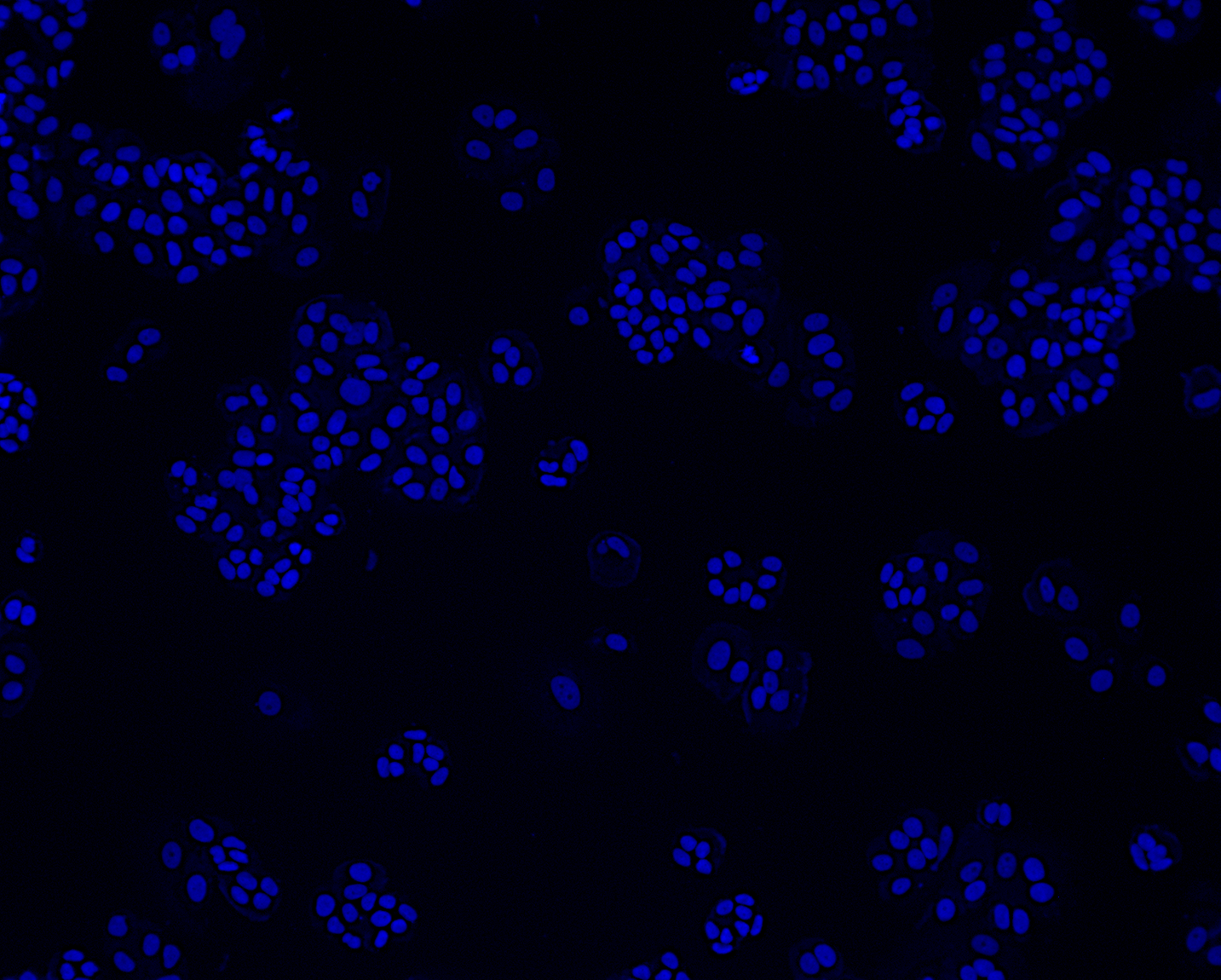

Supplement: Supplementary file 19 [file DataSheet7.ZIP › 拍摄-1284-图像导出-49.tif]

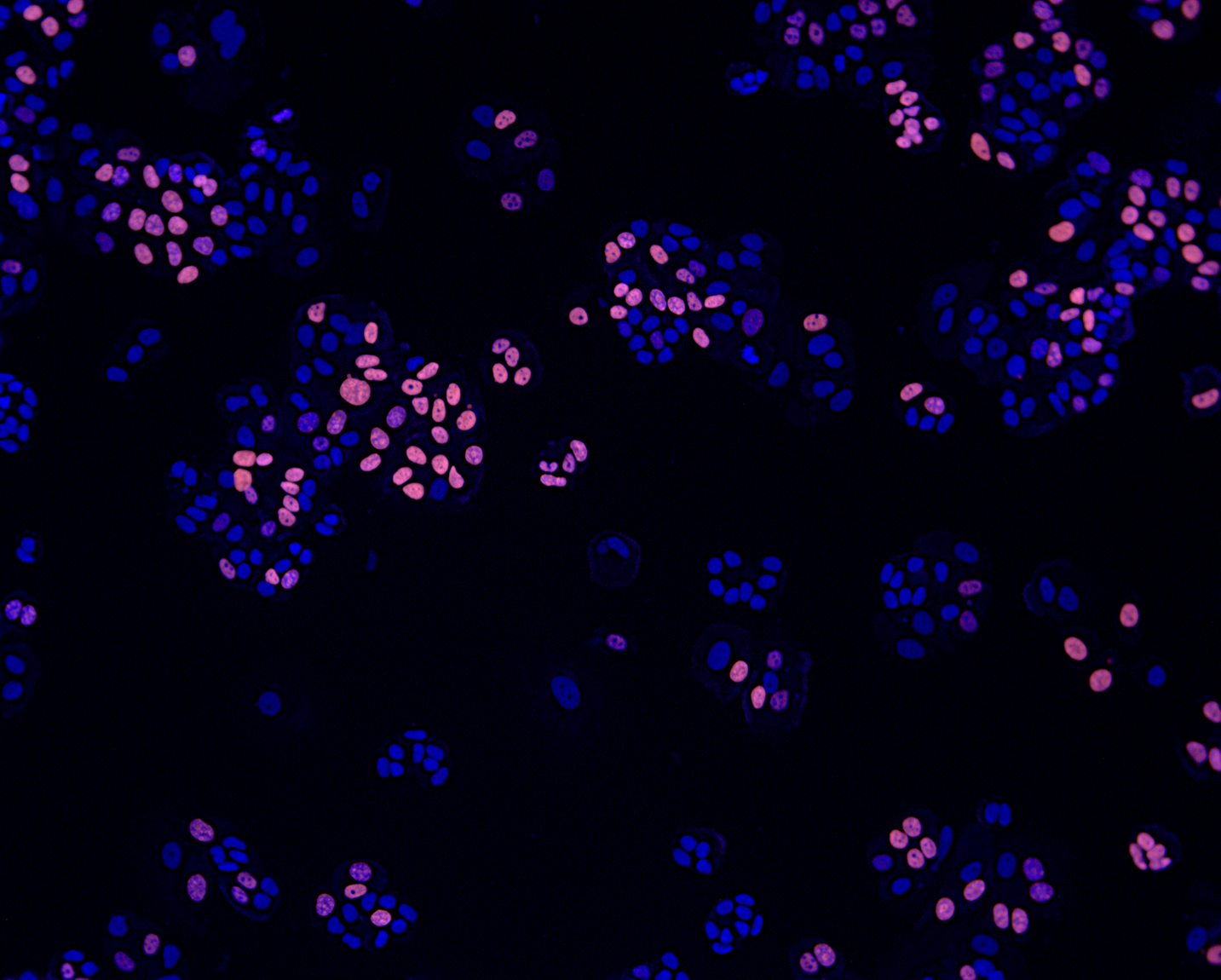

Supplement: Supplementary file 19 [file DataSheet7.ZIP › 拍摄-1284-添加通道-19-图像导出-50_c1+2.tif]

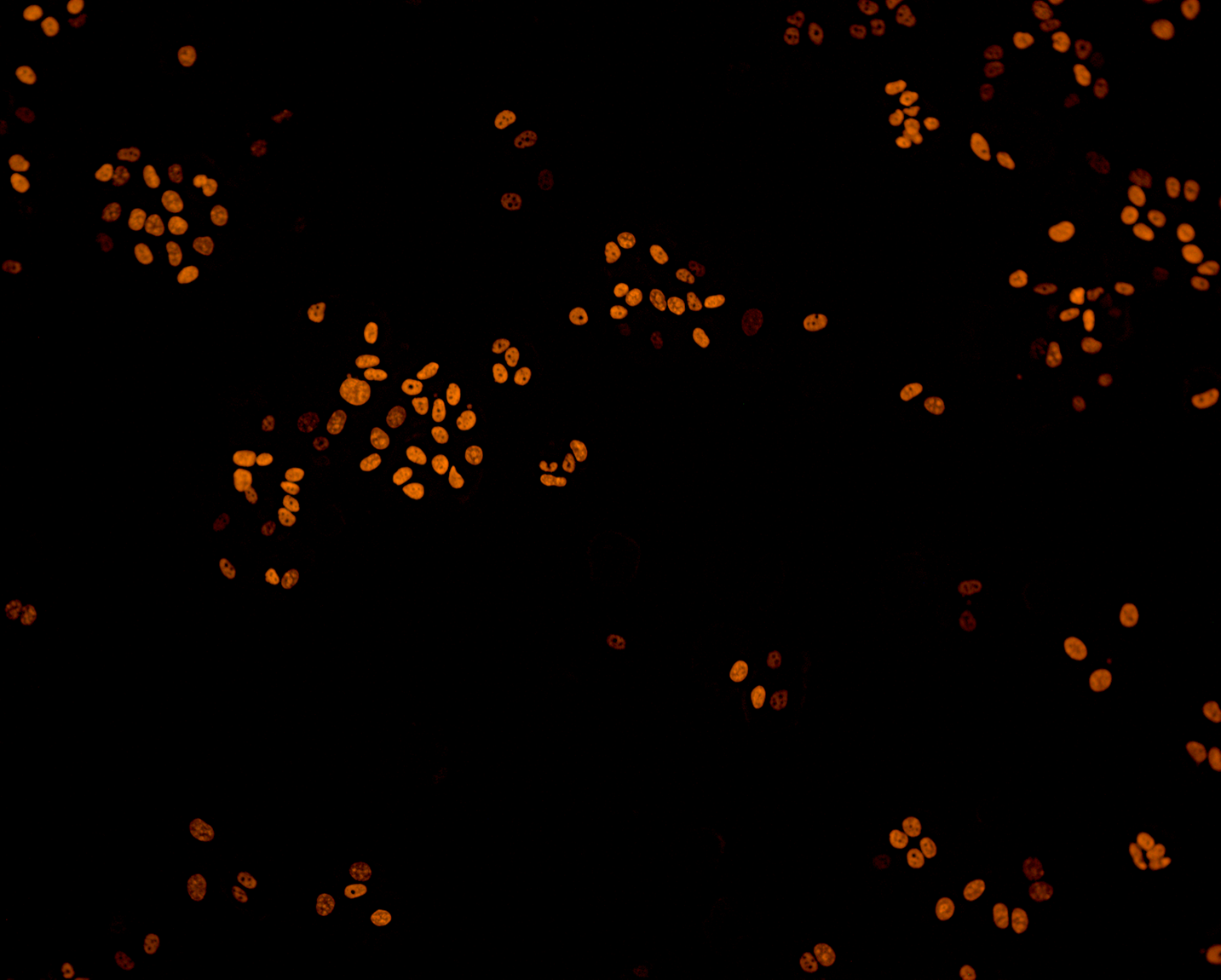

Supplement: Supplementary file 19 [file DataSheet7.ZIP › 拍摄-1285-图像导出-51.tif]

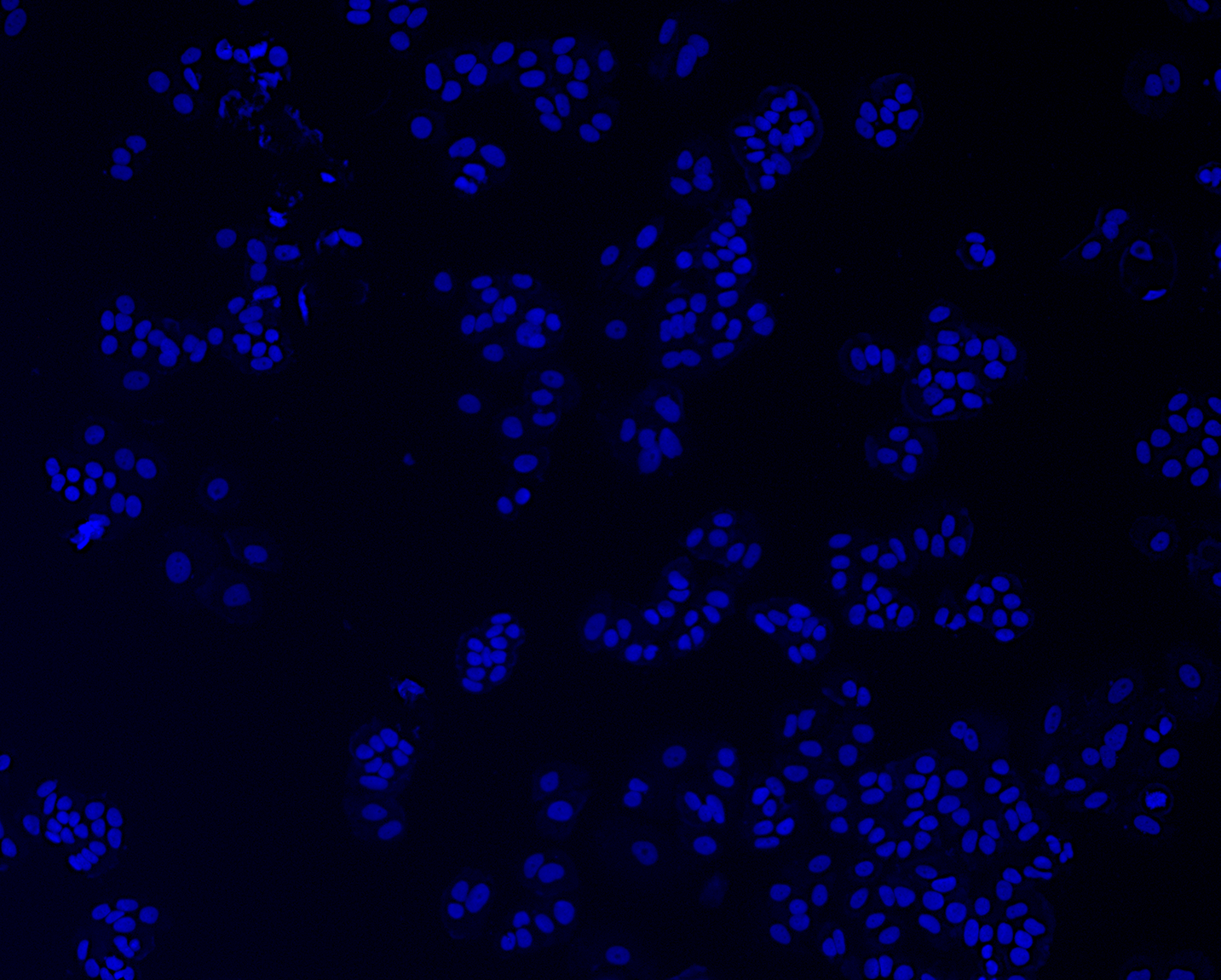

Supplement: Supplementary file 19 [file DataSheet7.ZIP › 拍摄-1286-图像导出-52.tif]

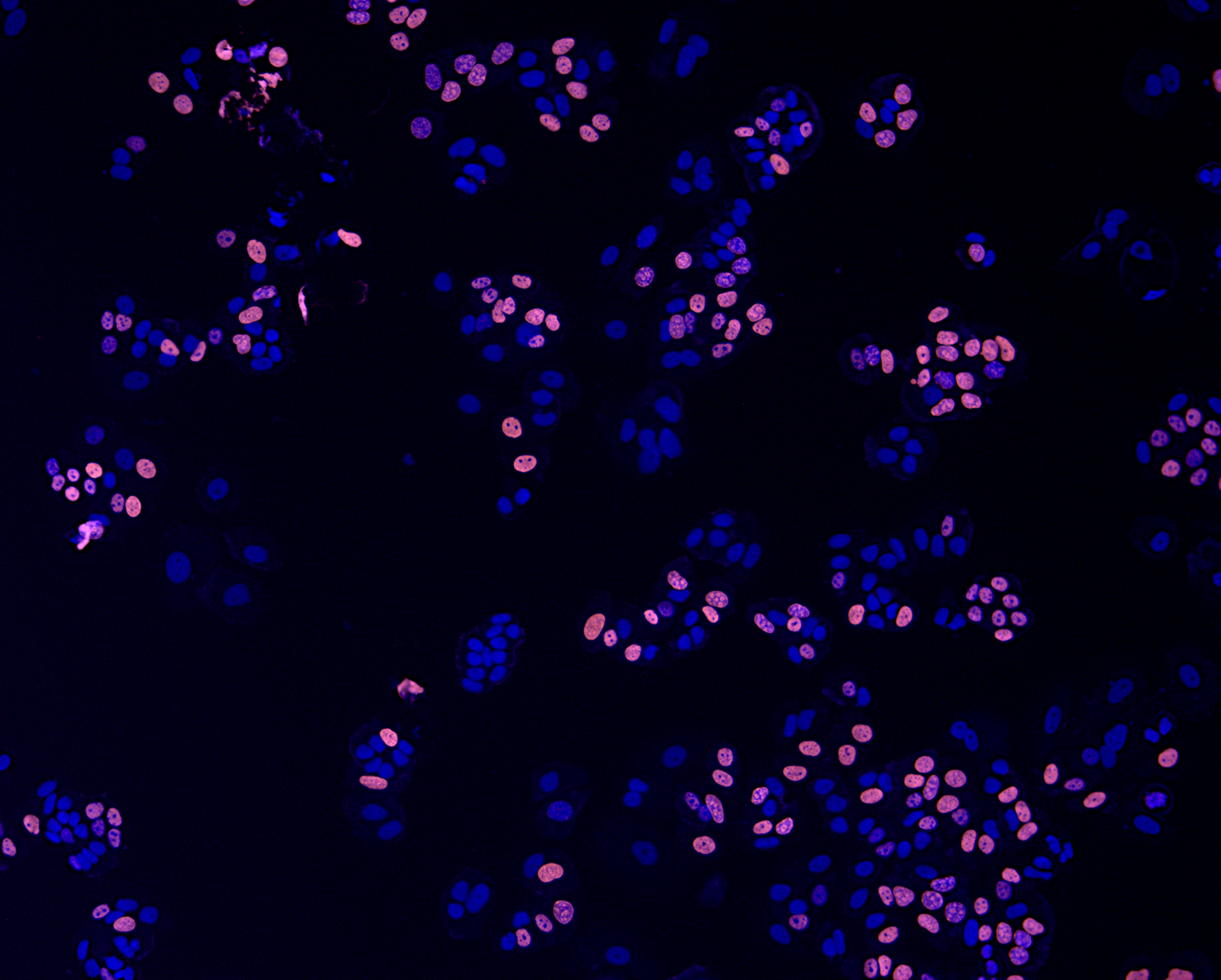

Supplement: Supplementary file 19 [file DataSheet7.ZIP › 拍摄-1286-添加通道-20-图像导出-53_c1+2.tif]

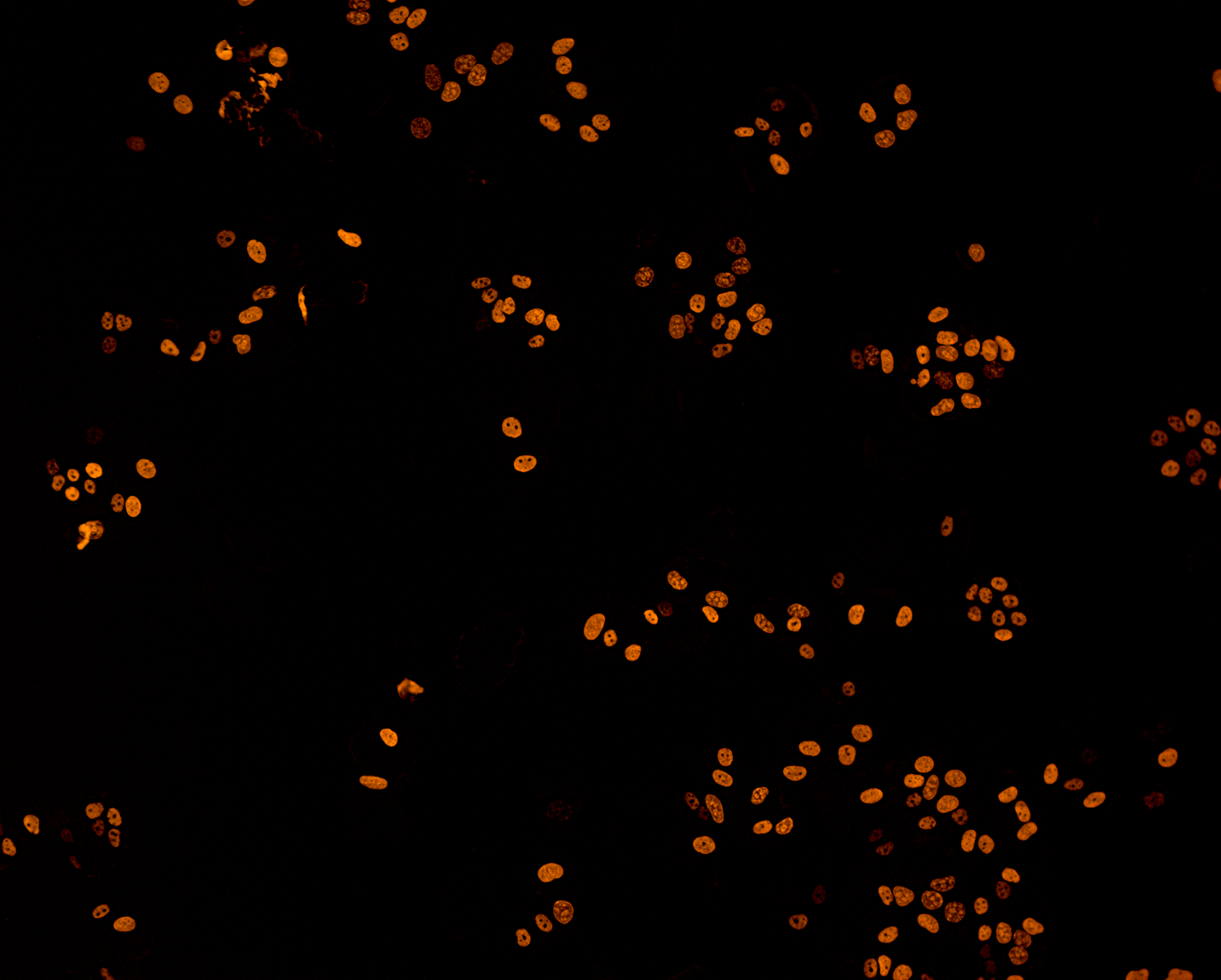

Supplement: Supplementary file 19 [file DataSheet7.ZIP › 拍摄-1287-图像导出-54.tif]
