# Supplementary material for: Integrative analysis of the role of BOLA2B in human pan-cancer
Source: Front Genet. 2023 Feb 27;14:1077126. doi: 10.3389/fgene.2023.1077126 (PMC10008965; doi:10.3389/fgene.2023.1077126)
Supplement: Supplementary file 6 [file Table1.DOCX]

Appendix

| Gene name |
| --- |
| BOLA2B |
| GRX3 |
| GRX4 |
| TET2 |
| NSUN5 |
| ALYREF |
| KIAA1429 |
| EDNRB |
| ENTPD1 |
| TLR4 |
| TP53 |
| TTN |
| MUC16 |
| AFT1 |
| AFT2 |
| mreB |
| ydaM |
| yhjH |
| ydiV |
| yliE |
| yahA |
|  |
