# Supplementary material for: Integrative analysis of the role of BOLA2B in human pan-cancer
Source: Front Genet. 2023 Feb 27;14:1077126. doi: 10.3389/fgene.2023.1077126 (PMC10008965; doi:10.3389/fgene.2023.1077126)
Supplement: Supplementary file 18 [file DataSheet10.PDF]

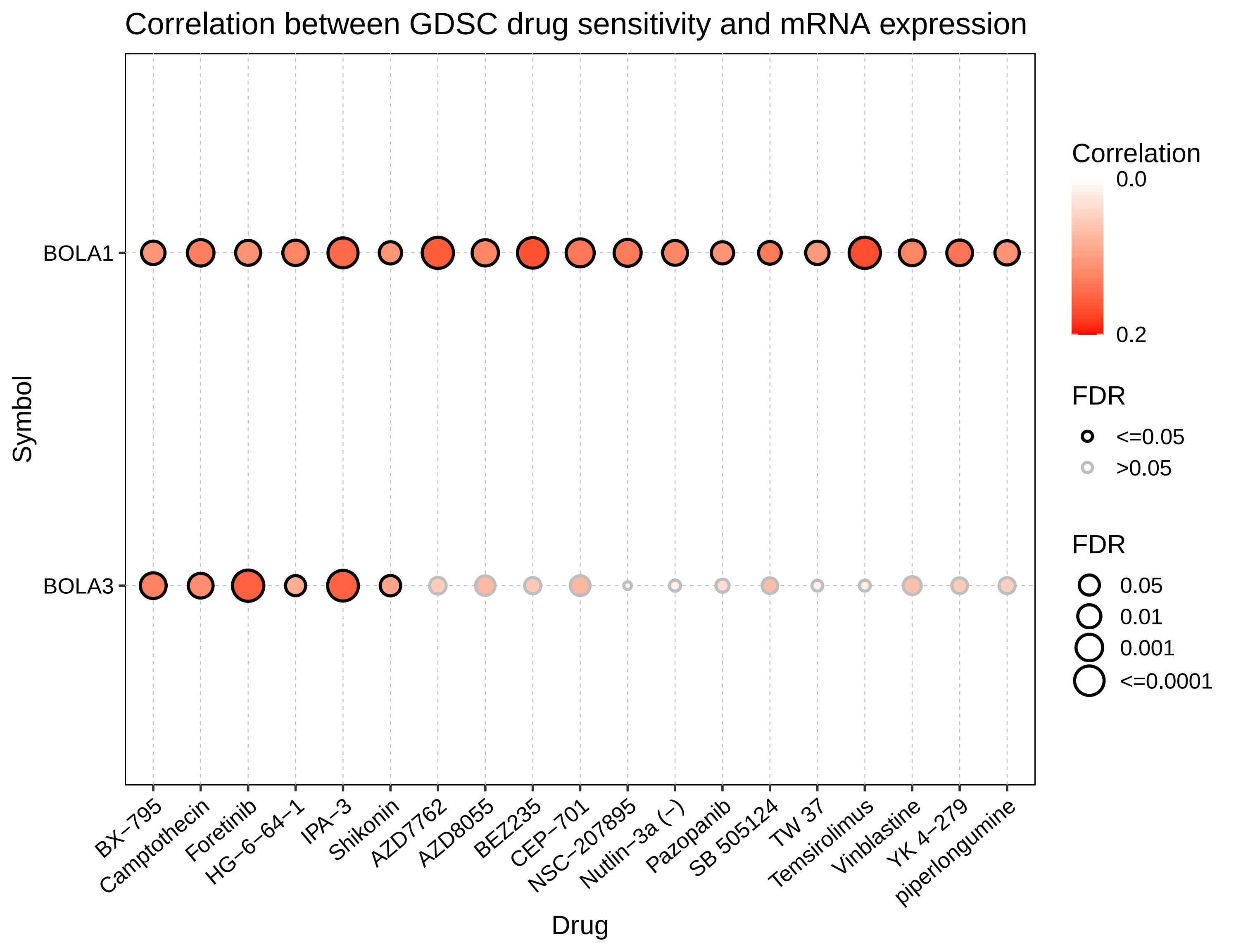

Correlation between GDSC drug sensitivity and mRNA expression
Symbol
BOLA1
BOLA3
Drug
BX−795
Camptothecin
Foretinib
HG−6−64−1
IPA−3
Shikonin
AZD7762
AZD8055
BEZ235
CEP−701
NSC−207895
Nutlin−3a (−)
Pazopanib
SB 505124
TW 37
Temsirolimus
Vinblastine
YK 4−279
piperlongumine
Correlation
0.0
0.2
FDR
<=0.05
>0.05
FDR
0.05
0.01
0.001
<=0.0001
